# Context-dependent DNA polymerization effects can masquerade as DNA modification signals

**DOI:** 10.1186/s12864-022-08471-2

**Published:** 2022-03-31

**Authors:** Yusuke Takahashi, Massa Shoura, Andrew Fire, Shinichi Morishita

**Affiliations:** 1 Department of Computational Biology and Medical Sciences, Graduate School of Frontier Sciences, The University of Tokyo, Tokyo, Japan; 2 Departments of Pathology and Genetics, School of Medicine, Stanford University, Stanford, CA USA

**Keywords:** DNA polymerization, DNA modification, Non-B DNA, Whole genome amplification, Single-molecule real-time (SMRT) sequencing, DNA N6-methyladenine

## Abstract

**Background:**

Single molecule measurements of DNA polymerization kinetics provide a sensitive means to detect both secondary structures in DNA and deviations from primary chemical structure as a result of modified bases. In one approach to such analysis, deviations can be inferred by monitoring the behavior of DNA polymerase using single-molecule, real-time sequencing with zero-mode waveguide. This approach uses a Single Molecule Real Time (SMRT)-sequencing measurement of time between fluorescence pulse signals from consecutive nucleosides incorporated during DNA replication, called the interpulse duration (IPD).

**Results:**

In this paper we present an analysis of loci with high IPDs in two genomes, a bacterial genome (*E. coli*) and a eukaryotic genome (*C. elegans*). To distinguish the potential effects of DNA modification on DNA polymerization speed, we paired an analysis of native genomic DNA with whole-genome amplified (WGA) material in which DNA modifications were effectively removed. Adenine modification sites for *E. coli* are known and we observed the expected IPD shifts at these sites in the native but not WGA samples. For *C. elegans*, such differences were not observed. Instead, we found a number of novel sequence contexts where IPDs were raised relative to the average IPDs for each of the four nucleotides, but for which the raised IPD was present in both native and WGA samples.

**Conclusion:**

The latter results argue strongly against DNA modification as the underlying driver for high IPD segments for *C. elegans*, and provide a framework for separating effects of DNA modification from context-dependent DNA polymerase kinetic patterns inherent in underlying DNA sequence for a complex eukaryotic genome.

**Supplementary Information:**

The online version contains supplementary material available at 10.1186/s12864-022-08471-2.

## Background

**Fig. 1:**
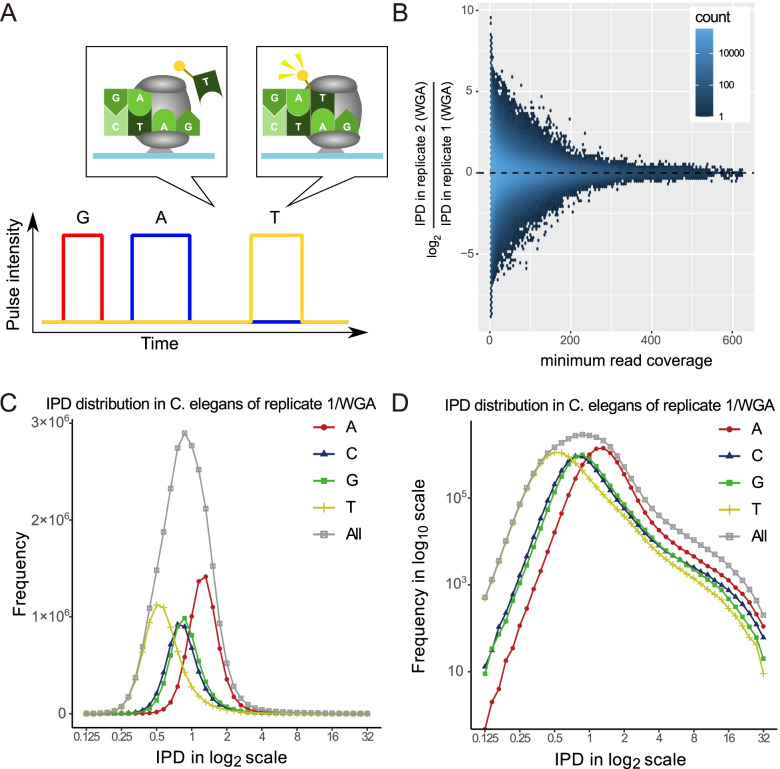
Interpulse duration (IPD) distributions from SMRT sequencing of *C. elegans* DNA samples. **A** The figure illustrates how a zero-mode waveguide monitor fluorescence signals from labeled nucleotides incorporated during DNA replication of the single-strand template of 5′-GATC-3′. The interpulse duration (IPD), the time between pulse signals from consecutive nucleosides, is useful in detecting methylated or damaged nucleotides in bacterial genomes because of slower incorporation of nucleotides by DNA polymerase. **B** Hexbin plot of the logarithmic scale ratio of IPD in replicate 2/WGA to IPD in replicate 1/WGA at each base for the minimum read coverage shown in the x-axis in each strand of the WGA samples. The IPDs fluctuate remarkably between the two WGA biological replicates when the minimum read coverage is low. **C** The frequency distribution of the IPDs in the x-axis (in log2 scale) of all bases and of individual four bases. **D** The y-axis of Fig. **C** is represented in log_10_ scale to highlight the frequencies of high IPDs ≥2

DNA polymerization kinetics on a single molecule level provide a window on both chemical modification of bases and sequence contexts that form tertiary structures, including hairpin loops and G-quadruplexes, which have been reported to cause DNA instability and alter gene transcription [1–4]. To measure DNA polymerization speed, single-molecule, real-time (SMRT) sequencing has been widely used through the use of a zero-mode waveguide (ZMW) to detect fluorescence signals from labeled nucleotides incorporated during DNA replication [5, 6–8]. When monitoring DNA polymerization speed at single nucleotide resolution, it is useful to measure the interpulse duration (IPD, Fig. 1A), which is the time between pulse signals from consecutive nucleosides. Although the DNA polymerases used in SMRT sequencing are not native but are optimized for better sequencing [9], SMRT sequencing data can be used to assess the effects of sequence contexts on the function of DNA polymerases.

Another relevant factor that can interfere with DNA polymerase is DNA methylation, a fundamental biological process that plays a crucial role in the restriction-modification (RM) system in bacteria [10], suppresses the transposition of transposable elements, and regulates gene expression in various eukaryotes [11].

*Caenorhabditis elegans* provides a useful pilot in which there is a value in distinguishing between covalent and sequence-based effects on DNA polymerization speed. *C. elegans* has been reported to lack DNA methylation on cytosines [12] and characterized DNA methyltransferase (*dnmt*) loci [13] and it had been suggested that DNA methylation may not occur in *C. elegans*, and that histone modifications may be responsible for regulating chromatin structure [14] and silencing repetitive transgenes [15] in *C. elegans*.

In a recent article, Greer et al. [16] reported observations suggesting the presence of DNA N6-methyladenine in the *C. elegans* genome, inferring this (amongst other methodologies) from SMRT sequencing. Potential modifications in the case of that publication were inferred based on the fact that methylated or damaged nucleotides tend to exhibit longer IPD than unmodified nucleotides in a negative control, due to the slower incorporation of nucleosides by DNA polymerase [17, 18]. The ratio, called the IPD ratio, was observed to become significantly higher in reading N6-adenine methylated bases in bacteria [19, 20]. Applying similar approaches, N6-methyladenine modifications have been suggested in a number of other multicellular eukaryotes [21], including *Chlamydomonas reinhardtii* [22, 23], *Drosophila melanogaster* [24], *Mus musculus* [25, 26], *Danio rerio*, *Sus scrofa* [27], *Xenopus laevis* [28], fungi [29], *Oryza sativa* [30], *Homo sapiens* [31], and *Bombyx mori* [32]. Despite the paper [16] from 2015, the presence of modifications in *C. elegans* remains undetermined; in particular, a subsequent paper including some of the original authors on the 2015 contribution [33] indicated that some or all of the *C. elegans* N6-adenine methylation may have resulted from non-*C. elegans* sources. In [33], the authors report that using UHPLC-ms/ms they find “low to undetectable levels of 4mC and 6mA in genomes of representative worms, insects, amphibians, birds, rodents and primates under normal growth conditions,” implying that N6-A methylation is not a general feature of eukaryotic genomes. In considering the SMRT data, a challenge has been that no negative control data (SMRT sequence profiles from DNA without methylation) were available; previous studies (e.g., [16]) had inferred expected IPD ratios for comparison from a computationally predicted training dataset from several bacteria [34, 35]. Given that the more recent work on *C. elegans* failed to observe consistent m6A signals from mass spectrometry [33], the presence of this modification remains to be assessed.

In this study, we used SMRT sequencing to collect data from *C. elegans* native DNA and to compare this with negative control data from whole-genome amplified (WGA) *C. elegans* samples that were free of DNA methylation. While we observe no evidence for methylated sites in the *C. elegans* genome (i.e., no substantial differences between WGA and unamplified DNA), we found clear differences between the observed IPDs and the IPDs predicted computationally by standard models. This work uncovers a number of novel sequence motif contexts with intrinsically high IPDs, indicating a family of sequences exhibiting slower incorporation of nucleosides by DNA polymerase.

## Results

### Whole-genome amplification as a negative control for DNA methylation

For our analyses, we used four samples from *C. elegans* strain VC2010. Two samples were native replicates (denoted by replicate 1 and 2/native), while the other two samples were WGA replicates (denoted by replicate 1 and 2/WGA) and served as negative control samples, as they were presumed and later demonstrated (see below) to be essentially free of DNA methylation. All four samples were subjected to SMRT sequencing using the PacBio Sequel system (v2.1 chemistry) (see Table 1). Resulting reads were mapped to the reference genomes of *C. elegans* (ce11) and *E. coli*. The ratio of confounding read alignments with both *C. elegans* and *E. coli* genomes to all high mapping quality read alignments is smaller than 0.01% in all the samples (Supplementary Table 1), and hence the effects of confounding alignments are negligible (the rare confounding reads appear to exhibit artificial junctions; see examples in Supplementary Fig. 1A-B).

**Table 1:**
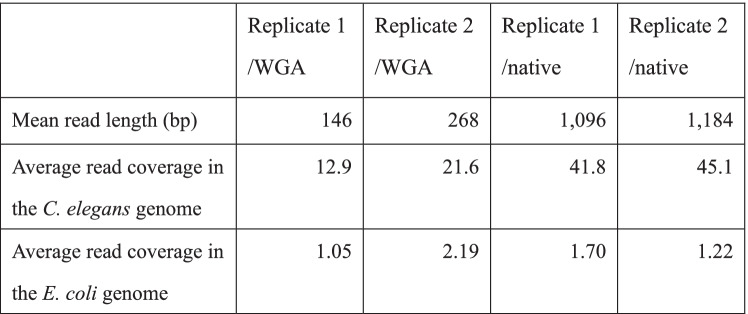
Mean read length and average read coverage per strand in each sample

For bacteria, SMRT sequencing has been widely used to identify methylated or damaged nucleotides of specific sequence motifs that exhibit slower incorporation of nucleosides by DNA polymerase and are likely to have higher IPDs. For example, for *E. coli* strains of the B class, N6-adenine methylation is found at the 2nd nucleotide of GATC, at the 5th in ATGCAT, at the 3rd in TGANNNNNNNNTGCA, and at the 4th in the reverse complement of the former motif. We compared IPDs at the adenines in the four motifs between the methylation-free WGA and native samples, and observed that the IPDs in the native samples were substantially larger than those in the WGA samples (Supplementary Fig. 2A and Supplementary Table 2); the increase in the adenine of GATC was particularly prevalent (213 GATC sites, 8.57-fold increase, and *p* < 10^− 99^ in the pair of replicate 2). We then examined the IPDs of adenines at the 2nd nucleotide of all 4-mers in the native and WGA replicate samples separately. We confirmed that only GATC had a significantly high average IPD in the native replicates (Supplementary Fig. 2B). This observation serves as a positive control for the SMRT sequencing method of detecting N6-methyladenine in *E. coli*. Amplification removes the increased IPDs observed at these sites. This result both confirms the connection between the increased IPD and DNA modification and indicates that our WGA procedure results in a DNA population where modifications have effectively been diluted through multiple rounds of amplification with unmodified nucleotides.

Comparing IPDs between native and WGA samples in the *C. elegans* genome, we found that a fraction of bases had distinct IPDs (two-dimensional plots of all IPD pairs in native and WGA samples are shown in Supplementary Fig. 3A and B). These differences are not due to DNA methylation effects on IPDs, since they are also observed in IPD values in the two WGA samples that lack DNA methylation (Fig. 1B and Supplementary Fig. 3C-E). Considering such variations as kinetic effects of DNA sequence in SMRT sequencing, we moved on to characterize the intrinsic effects of the DNA sequence on IPD kinetics using the WGA samples.

### IPDs of individual sites and sequence motifs in *C. elegans* genomic and amplified DNA

We first examined the frequency distribution of the IPDs of individual sites that were sufficiently covered by > 25 reads in the *C. elegans* genome of the replicate 1/WGA sample. The IPD distributions differed remarkably between the four bases; namely, the average IPDs of adenines, cytosines, guanines, and thymines were 1.38, 0.95, 1.00, and 0.65, respectively (Fig. 1C-D, Supplementary Table 3). Similar averages were observed in the *C. elegans* and *E. coli* genomes of the replicate 1/native and replicate 2 (WGA and native) samples (Supplementary Figures Table 3).

**Fig. 2:**
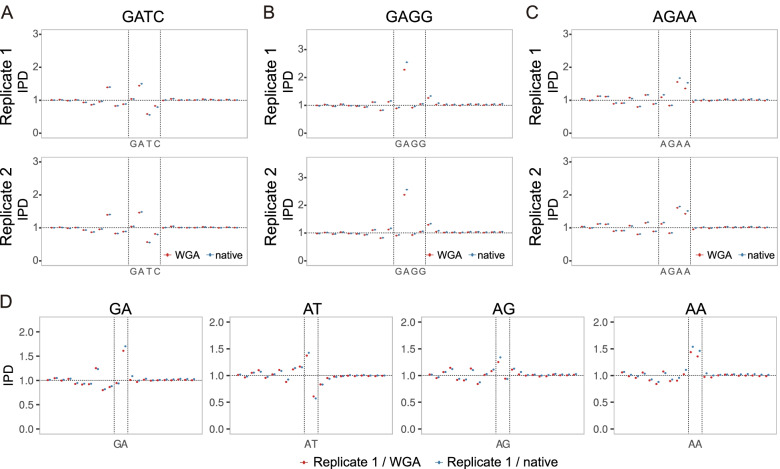
Consistency of IPDs between WGA and native samples for short motifs. **A-C** Concordance between the IPDs in the WGA (colored red) and native (blue) samples (replicate 1 in the 1st row, replicate 2 in the 2nd row). The charts show the IPD distributions represented by an error bar plot in the motifs and their surrounding 10 nucleotides in the x-axis. Nearly identical IPD distributions are obtained from the two biological replicates (0.99 < Pearson’s correlation coefficient; Supplementary Fig. 2C). Figure **A** displays GATC, where N6-methyladenine is prevalent in *E. coli*. Figure **B** and **C** show motifs that were reported to have N6-methyladenine in *C. elegans*. **D** IPD distributions in the two WGA replicates around the four 2-mer motifs (GA, AT, AG, and AA) that have adenines and occur in the 4-mer motifs, GATC, GAGG, and AGAA in Figure **A-C**. The A’s average IPD in GA are higher than those of the other three 2-mer motifs, and almost concord with the average IPDs of A in GATC and AGAA, but is much smaller than the A’s average IPD in GAGG

We then investigated the IPDs of several known motifs. The first motif investigated, provides a simple comparison to the bacterial DNA. The tetranucleotide GATC, which is known to be modified with N6-methyladenine in *E. coli,* had no indication of such modification in *C. elegans* [36]. Figure 2A and Table 2 show that the average IPDs of adenines are effectively identical (and not increased) between the WGA and native samples, although a slight sequence-specific increase of ~ 1.03-fold is observed in both the WGA and native samples. Figure 2B, C and Table 2 show GAGG and AGAA that were reported to have N6-methyladenine in *C. elegans* [16]. Although a slightly higher average IPD of the adenine in GAGG was measured in the native samples relative to WGA samples, the differences are not significant and are much smaller than the expected several fold difference for true methylation. Thus, the presence of N6-methyladenine in GAGG is questionable. The IPDs of the adenines in AGAA are also consistent between both of the WGA and native samples. Concludingly, in the light of the concordance between the WGA and native samples, DNA methylation in the three motifs is either absent or in very low abundance.

**Table 2:**
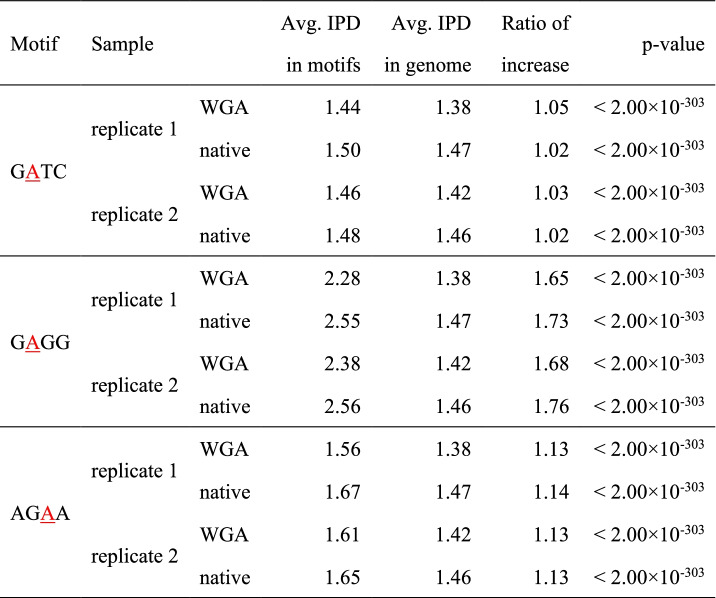
Concordance between the IPDs in the WGA and native samples. The table shows the statistics of the focal nucleotide with the maximum IPD (underlined and colored red) in each motif; namely, the average IPD of the focal nucleotide in all motif occurrences, the average IPD in the entire *C. elegans* genome, and the ratio of increase, the ratio of the average IPD in motif occurrences to that in the genome. The significance of the ratio of increase is confirmed by comparing the frequency distributions of the IPDs using Wilcoxon’s ranksum test (*p*-values shown in the last columns)

Of note, the three 4-mer motifs (GATC, GAGG, and AGAA) have the highest average IPD at the adenine in GA (Fig. 2, Supplementary Fig. 2C), motivating us to analyze the IPD distributions surrounding GA and the other 2-mers. We found that A in GA had the highest average IPD among adenines in all 2-mers (Supplementary Fig. 5), suggesting the simple hypothesis that the IPD distributions around 2-mers explain those around sequence motifs longer than 2-mers. The A’s average IPD in GA is almost the same as the average IPDs of A in GATC and AGAA; however, it is much smaller than the average IPD in GAGG (Fig. 2), denying the simple hypothesis. Thus, it is intriguing to understand what types of longer motifs remarkably affect DNA polymerization speed.

### Motifs with extreme IPDs show context-dependent DNA polymerization speed

**Fig. 3:**
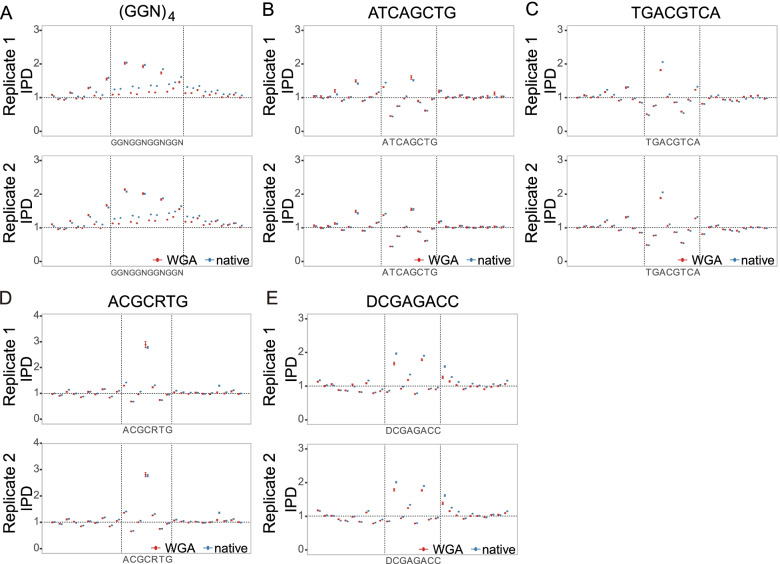
Consistency of IPDs between WGA and native samples for extended motifs. Similarly to Fig. 2, Figure **A-E** show motifs that have one or more bases with extremely high IPD. Figure **A**, **B**, and **C** show motif examples in non-B DNA. (GGN)4 in **A** may form G-quadruplexes that are associated with polymerization slowdown. AT(CAG)(CTG) in **B** and (TGAC)(GTCA) in **C** have quasi-palindromes that are pairs of reverse-complementary sequences in parentheses and might induce cruciform DNA structures associated with polymerization acceleration. Figure **D** and **E** show motifs with extreme IPDs at cytosines

We then searched for novel sequence motifs with extreme IPDs in the two VC2010 WGA samples. Specifically, we analyzed the sequences around loci with extreme IPD values that represented either the top 1% or the bottom 1% in the entire IPD distribution (Fig. 1C and Supplementary Fig. 4). We then examined the relationships between extreme IPDs and specific sequence motifs using the motif analysis program MEME-ChIP. This analysis revealed the presence of shared motifs in the two WGA samples. Figure 3A-E illustrate five representatives among 37 motifs with significantly extreme IPDs in comparison with the IPDs of four single bases in the whole genome (minimum *p*-values among the samples for each motif were less than 3.14 × 10^− 3^ after Bonferroni correction; Supplementary Fig. 6B-N and Supplementary Table 4), which demonstrate that DNA polymerization speed is not necessarily determined by single bases or 2-mers but can be context-dependent. We also examined the IPDs of these 37 motifs in SMRT sequencing data from the human genome (see Methods) and found that the IPDs of 31 motifs were significantly correlated between the human and VC2010 WGA datasets (*p*-values < 5% according to Pearson’s correlation coefficient analysis; Supplementary Fig. 7), indicating their intrinsic relevance to DNA polymerization speed.

The motifs include those prevalent in non-B DNA in human genomes and are correlated with polymerization slowdown or acceleration according to single-molecule real-time sequencing [37]. For example, Fig. 3A and Table 3 show that (GGN)4 is associated with polymerization slowdown (indicated by high IPDs) that might be caused by the formation of DNA tertiary structures such as G-quadruplexes. Figure 3B, C and Table 3 present AT(CAG)(CTG) and (TGAC)(GTCA), where pairs of sequences in parentheses are reverse-complementary and can form quasi-palindromes. Such inverted repeats have the potential to form cruciform DNA structures and could possibly generate structured DNA around the motifs that may allow polymerase to move at different rates. The IPDs of other motifs with quasi-palindromes, such as (GCGC)(GCGC) and (GC)(GC)GTCA, are given in Supplementary Fig. 6H and M.

**Table 3:**
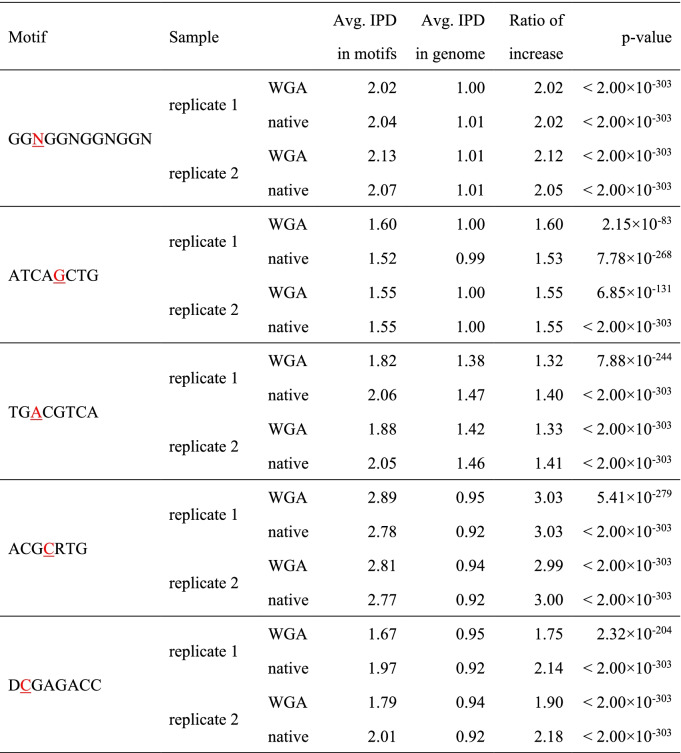
Similarly to Table 2, this table shows motifs that have one or more bases with extremely high IPD

In addition to these motifs in non-B DNA, Fig. 3D, E and Table 3 show other types of motifs such as ACGCRTG and DCGAGACC. These two motifs and other twenty-five motifs in Supplementary Fig. 6 do not lead to unusual structural consequences that we know of (Supplementary Table 4). Certainly, there may be many yet-to-be-characterized effects of DNA sequence on structure and interaction with polymerases, so the structures of these motifs and their interactions with polymerases will be worthy of future investigation.

Of some interest, in Figs. 2 and 3, we observed unusual high IPD signals outside of several motifs. These anomalies were also observed in both WGA and native samples. As examples, GATC and ATCAGCTG respectively had high IPDs at the positions three and four bases upstream of the motifs (Figs. 2A and 3B). We examined whether a single nucleotide was dominant at these positions and found that all nucleotides were present and had IPDs significantly greater than their averages in the entire genome (Supplementary Fig. 6O, and P). These motifs might be related to the increased IPDs at these specific positions outside the motifs either through direct effect on the DNA polymerase or through an association with a more complex upstream sequence feature.

### Discrepancy between observed and predicted IPDs in *C. elegans*

**Fig. 4:**
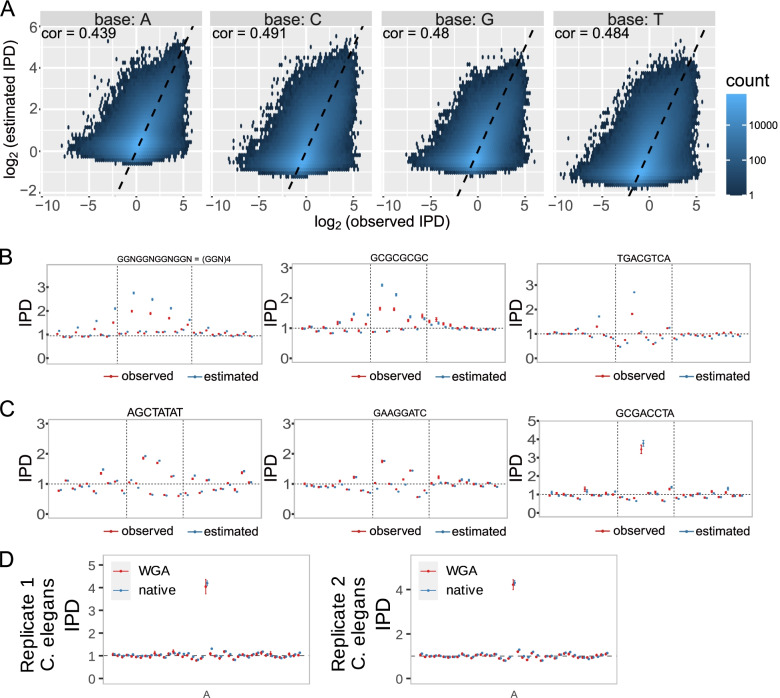
Discrepancy between the observed IPDs in the replicate 1/WGA sample and predicted IPDs in *C. elegans*. **A** Hexbin plot comparing the IPDs (x-axis) observed in the replicate 1/WGA sample with those estimated (y-axis)) using the PacBio software (SMRT Link 6.0.0.47841) for each type of base. Values are shown using a logarithmic scale. Inside each plot, cor represents the Pearson’s correlation coefficient. **B** Large discrepancies between the observed and estimated IPDs are seen at the bases with extreme IPDs in the three motifs; namely, at Ns in (GGN)4, Cs in GCGCGCGC, and the first A in TGACGTCA. **C** Predictions are almost consistent with observations in several motifs. Supplementary Figs. 12 and 13 show the observed and predicted IPDs around all motifs in the four samples. **D** The IPD distributions of our WGA and native samples almost agree, which suggests the absence of 6 mA, at the locations where 6mAs are called in the data that are used to report the presence of DNA N6-methyladenine in the *C. elegans* genome

In *C. elegans,* O’Brown et al. [33] suggest that most of the N6-adenine methylation detected by SMRT sequencing could be false-positive signals, presumably because the IPDs of local sequence contexts in negative control WGA samples are not observed in reality but are predicted by using the standard machine-learning method that is trained from several bacteria [34, 35]. Indeed, significant discrepancies between observations and predictions are seen from the relationship between IPDs of individual single bases in the two WGA samples and those predicted using the PacBio software program (SMRT Link v6.0.0.47841, Fig. 4A, Table 4 and Supplementary Figs. 8-9).

**Table 4:**
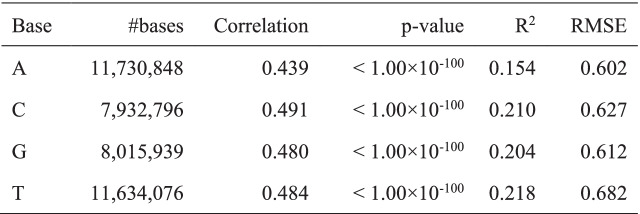
Discrepancy between the observed IPDs in the replicate 1/WGA sample and predicted IPDs in *C. elegans*. For each base, the table shows the number of bases, the Pearson’s correlation coefficient, *p*-value for a hypothesis that the correlation coefficient equals zero, R^2^ (coefficient of determination), and RMSE (root-mean-square error)

To check whether this difference is prevalent only on the *C. elegans* genome or is also present in the *E. coli* genome, we investigated IPDs on the *C. elegans* and *E. coli* genomes separately (Fig. 4A, Supplementary Figs. 8, 10-11), and we indeed confirmed the differences in both of the genomes. Figure 4B shows a large discrepancy between the observed and predicted IPDs of three motifs in replicate 1/WGA, though in several motifs, predictions were consistent with observations (Fig. 4C). Similar discrepancies can be seen in replicate 2/WGA as well as in the two native samples (see Supplementary Figs. 12-13). We then examined the reliability of 6 mA calls in the native sample that was used to report the presence of DNA N6-methyladenine in the *C. elegans* genome [16] by checking the difference between the IPD distributions of our WGA and native samples at the locations where 6mAs are called; however, we observed no remarkable difference (Fig. 4D), showing most or all of the previous 6 mA calls were false-positive due to the discrepancies between predicted and actual values.

An overall conclusion from this analysis is that the current IPD caller (based on bacterial genomes) is not infallible as a baseline for the assignment of modifications in a complex genome (in this case the *C. elegans* genome). Because of the nature of single outlying values in any distribution, it would seem likely that no single model would predict kinetic properties for a large and complex genome. Instead, definitive identification of modified bases in any genome would by nature require a direct comparison between native DNA and material with modifications removed (e.g., using the WGA amplification approach here) or material with modifications introduced by methyltransferases [38].

## Discussion

We have described the use of single molecule modification-sensitive native genomic DNA sequencing combined with a whole-genome-amplified (unmodified) DNA control to distinguish base modification from kinetic effects of DNA sequence in complex genomes. The context for this analysis is a number of studies where possible modification signals were identified but where interpretation was limited due to a lack of an unmodified reference. Here we show that such a homologous unmodified reference can provide a critical standard for rare and potentially complex signals in DNA that show anomalous kinetics in the SMRT sequencing platform.

When measuring IPD ratios in native samples, it is not always feasible to have negative control samples using whole-genome amplification, and hence it is desirable to have a computational tool that can simulate the IPD of each nucleotide solely from its sequence context in WGA samples. The software tool that has most commonly been used for this purpose is tuned to bacterial genomes and produced IPDs that were in some cases discordant with those from WGA samples for the worm genome. With WGA datasets from an autologous genome, it becomes possible to develop an accurate IPD caller for the any genome for specific study of known or novel modifications.

Sequences capable of retarding DNA polymerase could reflect various chemical and biological aspects of DNA structure. We found that loci with high IPDs were significantly enriched in exons, enhancers, and 5′ UTRs, while they tended to be absent from introns, 3′ UTRs, and tandem repeats (Supplementary Figs. 14A and 15; *q* < 0.1%). In contrast, loci with low IPD values were significantly enriched in promoters, 3′ UTRs, and introns, whereas they were absent from exons and tandem repeats (Supplementary Figs. 14B and 16; *q* < 0.1%). Importantly, we found a significantly positive correlation in the fold changes of genomic enrichment of high IPDs among all pairs of samples (Supplementary Fig. 17A; *p* < 0.1%); a weak positive correlation was observed for loci with low IPD values (Supplementary Fig. 17B). These data suggested an association between specific classes of genomic regions and bases with high or low IPD values. However, it does not appear that these positions with high IPDs shared common sequence motifs. It remains to be understood why those motifs tend to be conserved in functionally relevant genomic regions.

The fact that we failed to detect extensive adenine modification in our analysis indicates that the standard food source might not lead to pass-through incorporation of alternative nucleosides present in *E. coli* DNA. Nonetheless, there is precedent for pass through of certain dietary nucleotides, as observed experimentally for Bromodeoxyuridine [39]. It is conceivable that equivalent non-position-specific incorporation of 6-Me-Adenine at low levels might occur in *C. elegans* fed on *E. coli*, but this would need to be below the bulk detection limits of O’Brown et al [33] and without specific sites in the *C. elegans* genome showing focal methylation (from this work).

## Conclusions

To provide a definitive means to interpret potential DNA modification signals in single molecule sequencing data, we collected parallel data from native (unamplified) whole genome samples and samples stripped of modification through a whole genome amplification protocol. For the *E. coli* genome, which is known to carry modified 6-methyl adenosines at specific sites, comparisons between native and amplified samples confirmed the expected presence of distinctive native-specific kinetic effects at known positions of 6-methyl adenine residues. For a model eukaryotic genome (*C. elegans*) where the presence of functional 6-methyl adenine residues has been suggested but called into question in recent publications [16, 33], our comparison showed no evidence for such modification. This comparative approach thus provides an effective means to distinguish modification-based and sequence-based alterations in DNA polymerase kinetics.

Sequence-based modifications in kinetic data also provide a window on the interactions between DNA sequence, structure, and the speed of elongation of the DNA polymerase. We identified sequences with extreme IPDs that include both known motifs associated with non-B DNA structure that affect DNA polymerase elongation [37] and a number of additional motifs of unknown structural consequence that will certainly merit further study.

## Methods

### DNA sequencing

*C. elegans* strain VC2010 (hermaphrodite) was obtained from Caenorhabditis genetics center (St. Paul, MN, USA), and cultured with *E. coli* strain OP50, which is a common feed of *C. elegans*. A DNA sample from the *C. elegans* strain VC2010 (i.e., “replicate 2/native”) and a WGA sample from VC2010 (i.e., “replicate 2/WGA”) were prepared. A DNA sample from the *C. elegans* strain VC2010 and *E. coli* strain OP50 was prepared (replicate 1/native); a WGA sample form was also prepared (replicate 1/WGA). All the DNA samples were extracted from the whole organisms of the *C. elegans* at the mixed developmental stage. WGA was done by a Nextera kit (tagmentation using Tn5 transposase) followed by polymerase chain reaction (PCR). These samples were sequenced using a PacBio Sequel sequencing system (binding kit: v2.1, sequencing kit: v2.1).

### Mapping of reads

Resulting reads were mapped to the *C. elegans* WormBase WS235 genomic assembly (annotated as ce11 in the UCSC assembly collection). To check if PacBio reads were correctly aligned to their original genome of either *C. elegans* or *E. coli*, we aligned reads to the two genomes using pbalign (blasr), estimated the probability of incorrect alignment *p* for each read alignment, and retained high mapping quality read alignments with extremely low incorrect alignment probability *p* such that *p* < 10^-12.7^ or in terms of widely-used MapQ score, MapQ(*p*) = − 10 log_10_ *p* > 127 = − 10 log_10_ 10^-12.7^. Mapped reads were then merged with the *E. coli* B strain REL606 genomic assembly (GenBank CP000819.1), which is most similar to *E. coli* strain OP50 (personal communication with Robin C. May at [40]). Table 1 shows the mean read length and the average read coverage per strand in each of the samples. Although reads collected from the WGA samples are shorter than those from the native samples, they are sufficiently long to call IPDs of individual bases.

### Calculations of IPD

Mapping of the reads and IPD data analysis were performed using pbsmrtpipe v0.66.0 software (SMRT Link 6.0.0.47841), using minor modifications for the base modification detection. We collected the IPDs of these reads at each position, trimmed outlier IPDs using the standard PacBio pipeline named “ipdSummary,” and calculated the average of the IPDs. Valid IPDs were defined as IPDs that were not considered outliers of the IPDs at the same locus; neighboring bases of the read matched a reference sequence.

### Detection of bases with extreme IPDs

Bases with high or low IPD were defined as bases that had IPD higher than the top 1% or lower than the bottom 1%, respectively. The “replicate 1” and “replicate 2” were defined as the combination of replicate 1/native and replicate 1/WGA, or the combination of the replicate 2/native and replicate 2/WGA, respectively.

### Feature enrichment analysis

Enrichment of high IPD loci, or low IPD loci in the different genomic regions was assessed. Gene annotation of the WormBase version WS267 (https://wormbase.org/) was used for this analysis. Relative enrichment of kinetic features in genomic regions was defined as the fold change in the fraction of the kinetic feature loci (i.e., fraction of kinetic feature loci in a genomic region divided by fraction of kinetic feature in the genome). To assess whether the fold changes significantly differed from 1, the two-sided binomial test was used; the size of a genomic region was used as the number of trials, the fraction of the kinetic feature loci in the genome was used as the probability of success, and the number of kinetic feature loci in the genomic feature region was used as the number of successes. Kinetic loci and genomic regions with valid IPD counts per strand of ≥25 were used for this analysis. The code for the feature enrichment analysis is available at [41].

### Motif searching

Sequences of 41 bp around bases with valid IPD counts (≥ 25) were subjected to motif analysis with MEME-ChIP version 5.0.4 [42], using the following settings: -time 300 -ccut 100 -fdesc description -order 1 -db db/WORM/uniprobe_worm.meme -meme-mod anr -meme-minw 4 -meme-maxw 30 -meme-nmotifs 8 -meme-searchsize 100,000 -dreme-e 0.05 -centrimo-score 5.0 -centrimo-ethresh 10.0.

### Checking 6 mA calls in a previous *C. elegans* study

To examine the reliability of 6 mA calls in the previous *C. elegans* study [16], we used the data available at http://datasets.pacb.com.s3.amazonaws.com/2014/c_elegans/list.html.

### Confirmation of *C. elegans* motifs in the human genome using publicly available datasets

To examine the 37 *C. elegans* motifs in the human genome, we used the human data of all runs with P6-C4 chemistry in the NCBI SRA database with the accession SRX1424851 [31] except for two non-P6-C4-chemistry runs SRR3085709 and SRR3085710 in SRX1424851. These data were from native samples. We compared the IPDs around the 37 *C. elegans* motifs between the human and two VC2010 WGA replicates datasets, and for each motif, we tested the null hypothesis that there was no correlation between any pair of the three datasets. To this end, between a pair of two datasets, we calculated Pearson’s correlation coefficients of mean values of log_2_ IPDs at the nucleotides of each motif. Calculations of Pearson’s correlation coefficients were also performed for the nucleotides in each motif and for the 10 nucleotides in the upstream region that were likely to have extreme IPDs. We used the latter case because considering the 10 upstream positions in addition to the positions within each motif provides more statistically reliable results (Supplementary Fig. 7).

### Data analyses and statistical analyses

Data analyses were performed using R (4.0.2) [43], R packages data.Table (1.13.0) [44], ggplot2 (3.3.2) [45], hdf5r (1.3.2) [46], fst (0.9.2) [47], cowplot (1.0.0) [48], Biostrings (2.56.0) [49], command line tools bedtools (v2.28.0) [50], SeqKit (v0.10.1) [51], and samtools (1.11) [52]. Statistical tests were two-sided unless stated otherwise. Scripts for IPD analysis are available at [41].

## Supplementary Information

**Additional file 1.**

## Data Availability

Our data are available at the Sequence Read Archive (SRA) with study accession PRJNA724924. The accession numbers of reads and IPD files are SRR14322326 and SRR14322325 for replicate1/WGA, SRR14322323 and SRR14322322 for replicate2/WGA, SRR14322324 for replicate 1/native, and SRR14322321 for replicate 2/native.

## Abbreviations

IPD: Inter-pulse duration
WGA: Whole genome amplification
FDR: False discovery rate
SMRT sequencing: Single-molecule, real-time sequencing
ZMW: Zero-mode waveguide
RM: Restriction modification
dnmt: DNA methyltransferase
6 mA: DNA N6-Methyladenine;
4mC: DNA N4-methylcytosin
N6-A: DNA N6-Methyladenine
UHPLC-ms/ms: Ultra-high performance liquid chromatography tandem mass spectrometry
ce11: A version of *C. elegans* reference genome
