# Supplementary material for: Context-dependent DNA polymerization effects can masquerade as DNA modification signals
Source: BMC Genomics. 2022 Mar 31;23:249. doi: 10.1186/s12864-022-08471-2 (PMC8973881; doi:10.1186/s12864-022-08471-2)
Supplement: Supplementary file 1 — Additional file 1. [file 12864_2022_8471_MOESM1_ESM.pdf]

Supplementary tables

Supplementary table 1: For each of four samples, the table shows the average read coverage per strand in *C. elegans*, the mean read length, the number of high mapping quality read alignments with *C. elegans* (X), the number confounding read alignments with both *C. elegans* and *E. coli* genomes (Y), and the ratio of confounding read alignments (Y/X). The ratio is smaller than 10^{-4} (0.01%) in all the samples. The values of X in the WGA samples are higher than those in the native sample simply because the mean read lengths of the WGA samples are lower than those of the native samples.

Sample	Average read coverage	Mean read length (bp)	Number of alignments (X)	Number of confounding alignments (Y)	Ratio Y/X
Replicate 1/WGA	12.9	146	16,998,228	1,488	8.75×10^{-5}
Replicate 2/WGA	21.6	268	16,605,308	1,103	6.64×10^{-5}
Replicate 1/native	41.8	1,096	7,307,206	191	2.61×10^{-5}
Replicate 2/native	45.1	1,184	7,373,465	149	2.02×10^{-5}

Supplementary Table 2: In the table, the columns labeled with "# motif occ." show the number of qualified motif occurrences covered by $\geq$ 25 long reads, "avg. IPD" the average IPDs of the focal adenine in the motif occurrences, and "ratio of increase" the ratio of increase in the average IPD from the WGA to native samples. The significance of the ratio of increase is assessed by comparing the frequency distributions of the IPDs in the WGA and native samples using Wilcoxon's ranksum test (*p*-values in the last columns). The ratio of increase ranged from 2 to 11, and *p*-values were less than 5% except for ATGCAT. The number of ATGCAT occurrences was small and the increase ratio was moderate.

sequence motif	sample name	WGA		native		ratio of increase	p-value
		# motif occ.	avg. IPD	# motif occ.	avg. IPD		
G<u>A</u>TC	replicate 1	594	1.40	428	13.50	9.66	2.13×10^{-148}
	replicate 2	830	1.54	213	13.18	8.57	2.64×10^{-100}
ATGC<u>A</u>T	replicate 1	29	1.37	14	3.00	2.19	6.73×10^{-1}
	replicate 2	33	1.39	20	3.28	2.36	2.39×10^{-1}
TG<u>A</u>NNNNNNNNTGCT	replicate 1	12	1.29	5	14.13	10.93	3.23×10^{-4}
	replicate 2	15	1.33	5	5.40	4.07	1.97×10^{-1}
AGC<u>A</u>NNNNNNNNTCA	replicate 1	14	1.51	5	10.21	6.78	1.72×10^{-4}
	replicate 2	15	1.15	5	7.37	6.42	1.29×10^{-4}

Supplementary Table 3: The table shows the average IPDs of individual sets of nucleotides in the entire *C. elegans* genome or *E. coli* genome and the number of qualified nucleotides that are covered by ≥ 25 long reads in each of the four samples. The average IPDs were consistent among replicate 1 (WGA/native) and replicate 2 (WGA/native). The sets of nucleotides summarized in the table are as follows: A, C, G, T, N (= {A,C,G,T}), H (={A,C,T}), and Y (={C,T}). The average IPDs of individual bases in the *E. coli* genome concord with those in the *C. elegans* genome, presumably because all samples used the same chemistry (v2.1).

		Sample: *C. elegans*				Sample: *E. coli*			
	Base	Replicate 1/ WGA	Replicate 2/ WGA	Replicate 1/ native	Replicate 2/ native	Replicate 1/ WGA	Replicate 2/ WGA	Replicate 1/ native	Replicate 2/ native
Average IPD in the entire genome	A	1.38	1.42	1.47	1.46	1.37	1.40	1.61	1.59
	C	0.95	0.94	0.92	0.92	1.01	1.01	0.96	0.98
	G	1.00	1.00	0.99	1.00	1.02	1.02	0.99	0.99
	T	0.65	0.63	0.60	0.61	0.64	0.64	0.62	0.64
	N	1.00	1.01	1.01	1.01	1.01	1.02	1.05	1.07
	H	1.00	1.01	1.01	1.01	1.01	1.03	1.07	1.09
	Y	0.78	0.76	0.71	0.72	0.84	0.84	0.79	0.81
Number of qualified nucleotides	A	7,367,495	13,350,861	42,823,567	45,027,596	29,113	38,214	20,157	11,540
	C	5,088,988	8,621,888	23,441,834	24,626,206	32,085	40,367	19,697	9,927
	G	5,220,984	8,976,693	24,230,223	25,348,113	33,164	43,934	22,002	11,523
	T	7,219,301	12,915,350	41,785,889	43,933,577	28,483	35,978	19,039	10,380
	N	24,896,768	43,864,792	132,281,513	138,935,492	122,845	158,493	80,895	43,370
	H	19,675,784	34,888,099	108,051,290	113,587,379	89,681	114,559	58,893	31,847
	Y	12,308,289	21,537,238	65,227,723	68,559,783	60,568	76,345	38,736	20,307

Supplementary Table 4: The table shows the summary of known tertiary structures of the motifs we identified around extreme IPDs.

Motif	Motif type	known tertiary structure motif	Comment (structure)
ACGCRTG	high IPD motif	No	
ATCAGCTG	high IPD motif	No	
(GGN)4	high IPD motif	Yes	G4 two-quartets / i-motif
RGTA	high IPD motif	No	
AAAABT	high IPD motif	Yes	A-tract
AGAGAGTA	high IPD motif	No	
AGCTATAT	high IPD motif	Yes	quasi-palindrome: AGC(TA)(TA)T
AGGCAGGC	high IPD motif	No	
ATGGGAYA	high IPD motif	No	
ATTGTTAC	high IPD motif	No	
CAAACTAC	high IPD motif	No	
CAGYTG	high IPD motif	Yes	quasi-palindrome: (CA)GY(TG)
CCAATCAG	high IPD motif	No	
CRACGAS	high IPD motif	No	
DCGAGACC	high IPD motif	No	
DGCTTC	high IPD motif	No	
GAAGGATC	high IPD motif	Yes	quasi-palindrome: (GA)AGGA(TC)
GATATRGY	high IPD motif	Yes	quasi-palindrome: G(AT)(AT)RGY
GCGACCTA	high IPD motif	No	
GCGCGCGC	high IPD motif	Yes	quasi-palindrome: (GCGC)(GCGC)
GGHGGY	high IPD motif	No	
GTAGATCA	high IPD motif	No	
GTATCGTA	high IPD motif	No	
TGACGTCA	high IPD motif & low IPD motif	Yes	quasi-palindrome: (TGAC)(GTCA)
TGGTGSA	high IPD motif	No	
YGGAR	high IPD motif	No	
ACATMTGG	low IPD motif	Yes	quasi-palindrome: A(CA)TM(TG)G
CTGDAR	low IPD motif	No	
TACTGTAG	low IPD motif	Yes	quasi-palindrome: (TAC)T(GTA)G
AATMAATA	low IPD motif	Yes	quasi-palindrome: A(AT)MA(AT)A
AGACGCAG	low IPD motif	No	
CGGYTTGA	low IPD motif	No	
CTKCAA	low IPD motif	No	
GCGCGTCA	low IPD motif	Yes	quasi-palindrome: (GC)(GC)GTCA
GTGTGTGY	low IPD motif	No	
TACCCCKA	low IPD motif	No	
TACCTTGA	low IPD motif	No	

Supplementary figures

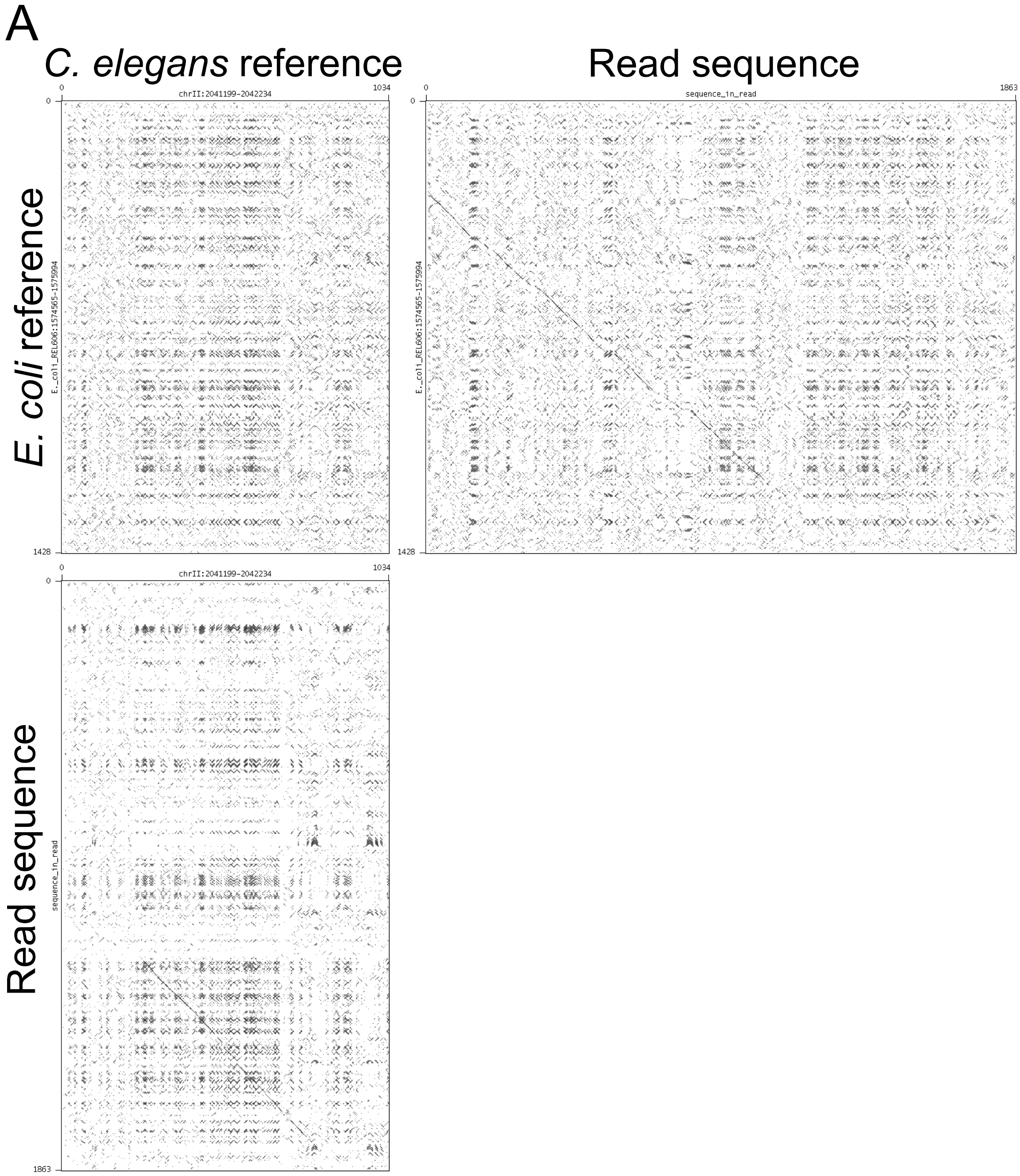

Supplementary Figure 1: (A) The top left dot plot (window length:10bp) shows an alignment between the *C. elegans* genome in the x-axis and the *E. coli* genome in the y-axis, and the bottom left (top right, respectively) dot plot shows alignments between the *C. elegans* (*E. coli*) genome and a typical artificially joined read whose first half and last half map to one of the *C. elegans* and *E. coli* genomes.

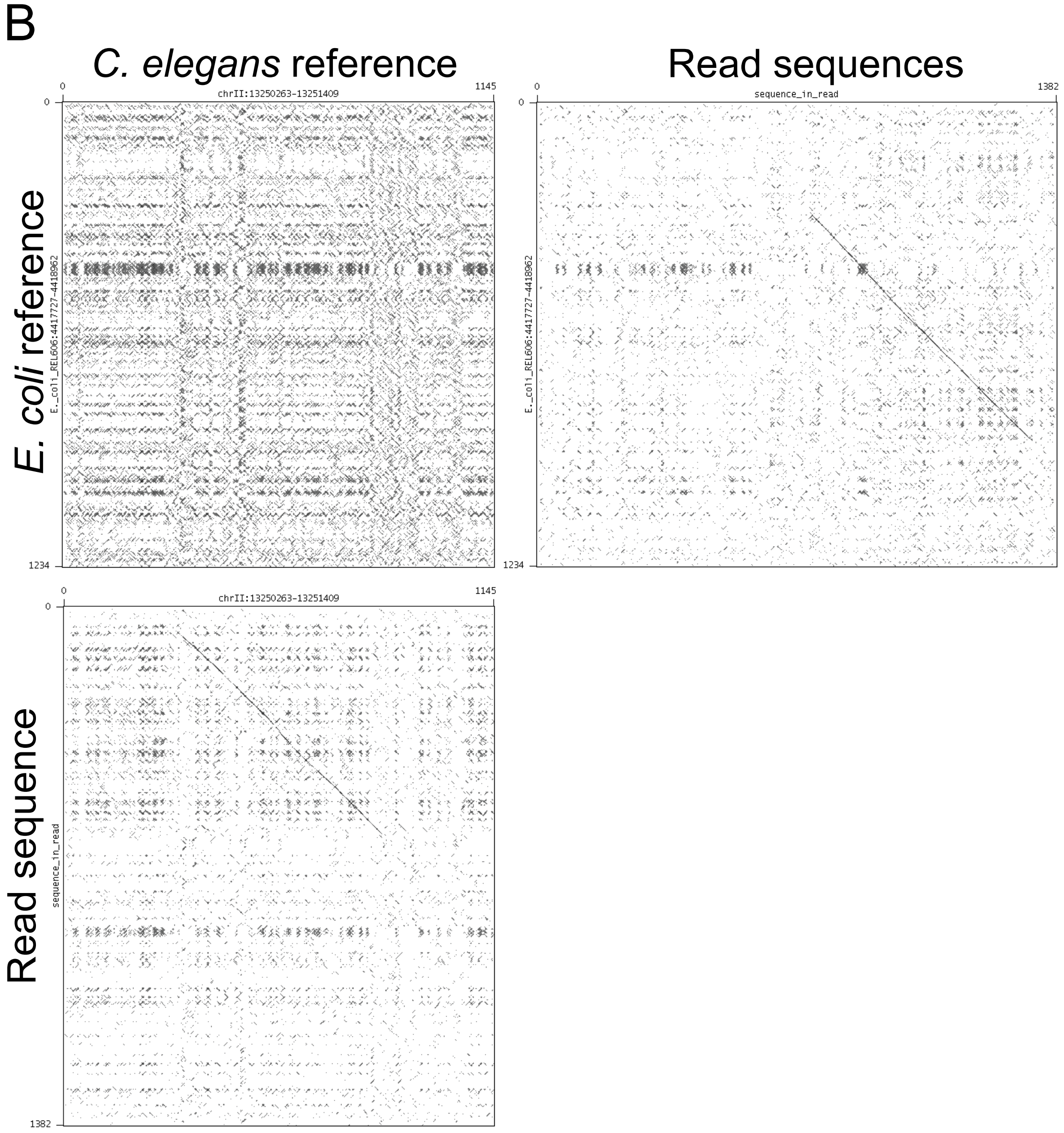

Supplementary Figure 1: (B) Dot plots for another example of artificially joined reads like Supplementary Figure 1A.

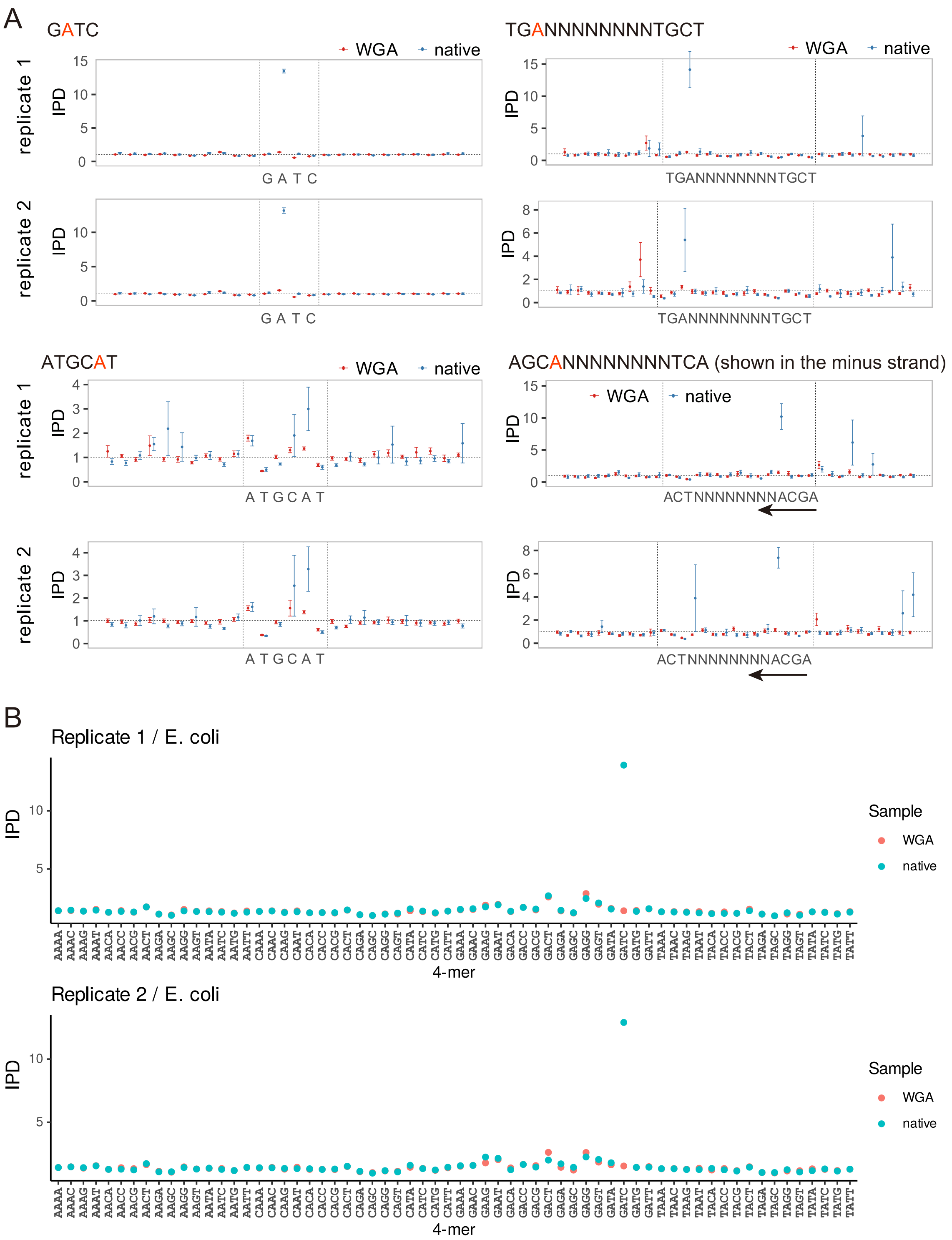

Supplementary Figure 2A-B: A. In the *E. coli* genome, we compared IPDs in the WGA (colored red) and native (blue) samples of two replicates for four motifs that are reported to have N6-adenine methylation. Specifically, the adenines highlighted read in four motifs, G<u>A</u>TC, ATGC<u>A</u>T, TG<u>A</u>NNNNNNNNTGCA, and AGC<u>A</u>NNNNNNNNTCA, are reported to have N6-adenine methylation in the *E. coli* genome. The last motif is the reverse complement of the third

and is shown in the direction of the minus strand to show the correspondence of the bases in the two motifs. **B.** In the *E. coli* genome, we compared the means of IPDs in the WGA (colored red) and native (blue) samples of two replicates for all the tetramers with an adenine at the second base.

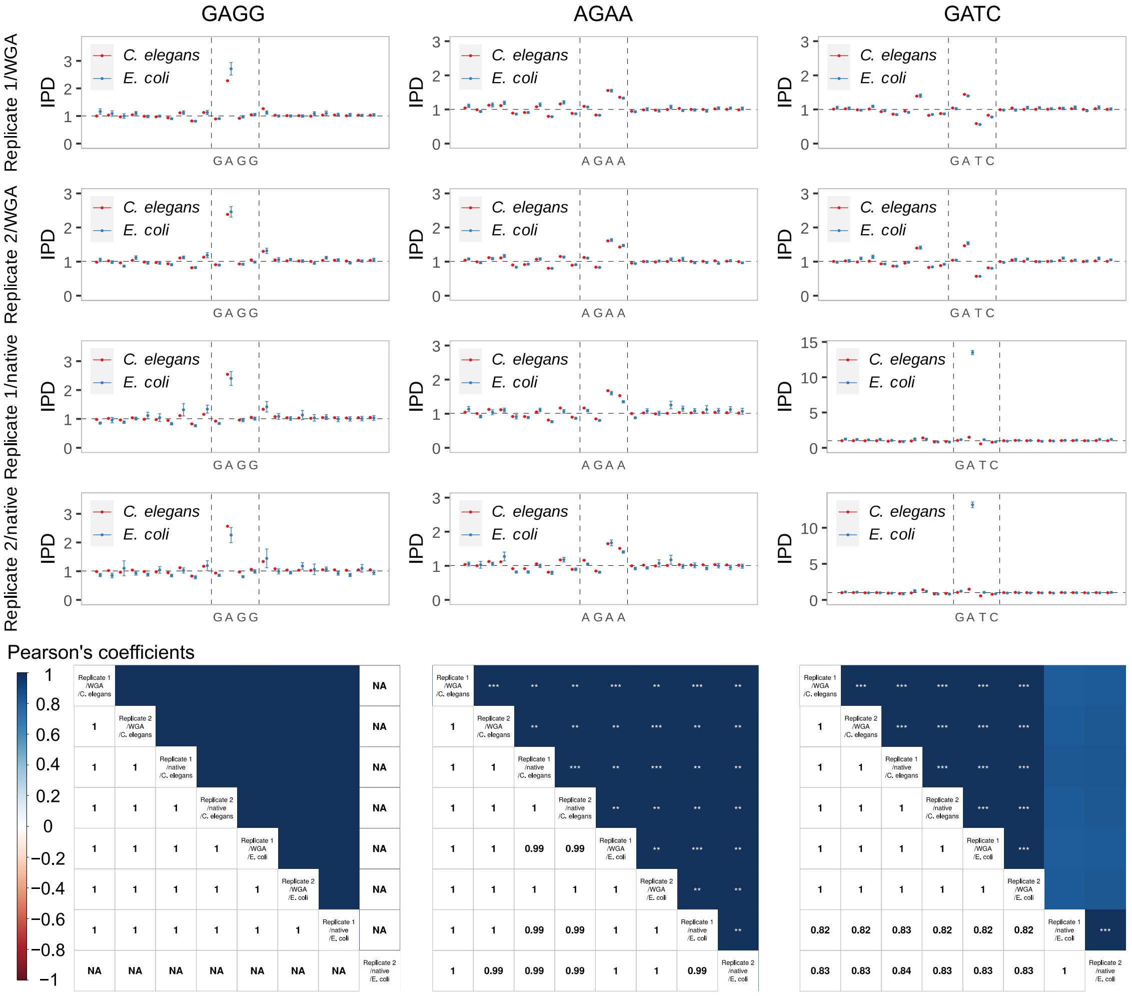

Supplementary Figure 2C: The upper four rows show the IPDs of the three motifs in the *C. elegans* (blue) and *E. coli* (red) genomes of four WGA or native samples. The bottom matrices present Pearson's correlation coefficients of mean values of $\log_2$ IPDs between pairs of the four samples observed in the *C. elegans* and *E. coli* genomes. In the bottom-left matrix, "NA" indicates that no Pearson's coefficients are available for the pair because in replicate 2/native, the *E. coli* genome fails to have 100 or more qualified occurrences of GAGG.

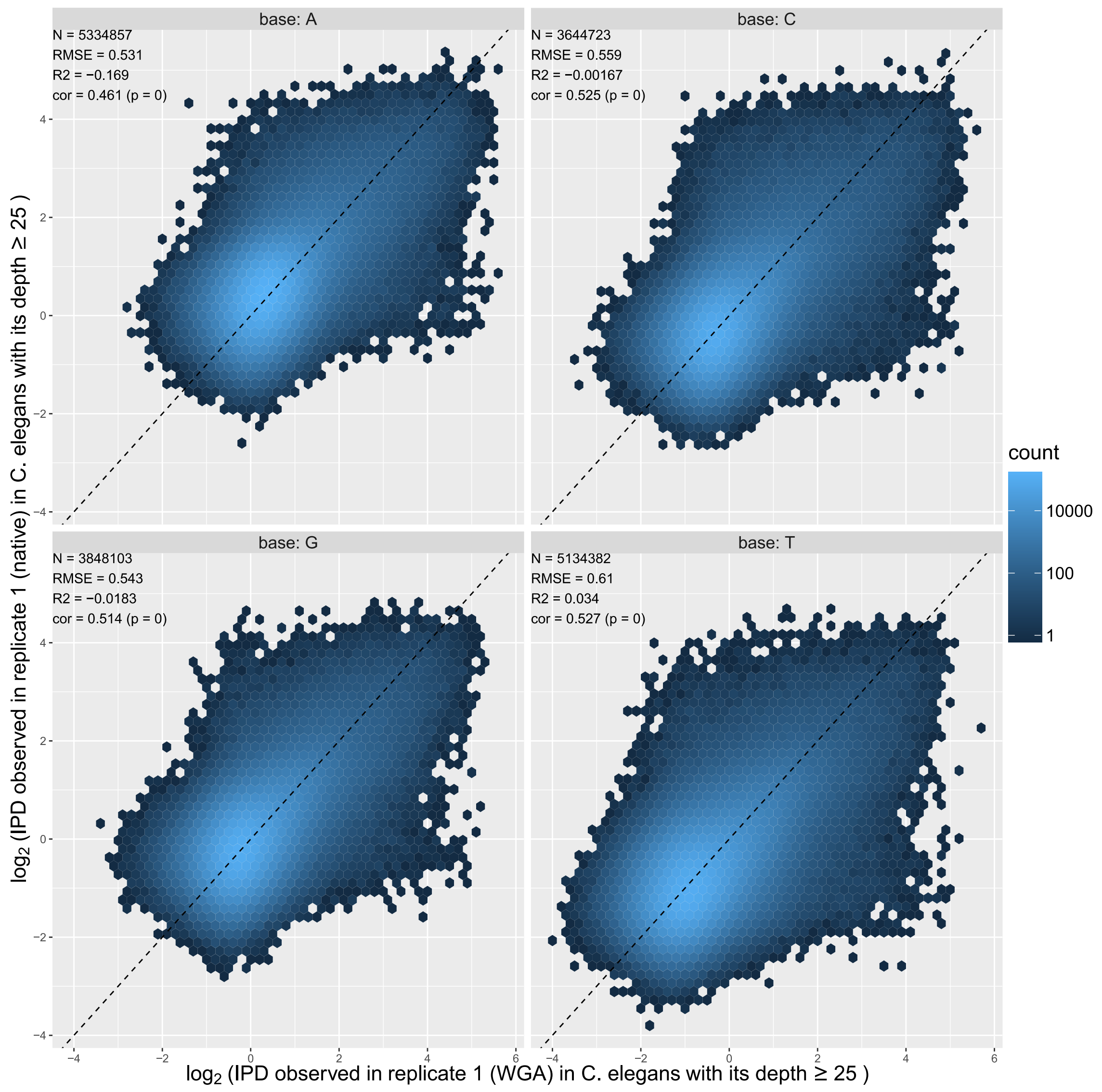

Supplementary Figure 3A: Hexbin plot comparing $\log_2$ (IPDs observed in replicate 1/WGA) with $\log_2$ (IPDs observed in replicate 1/native) for each of the bases with valid IPD counts (or depths) (≥ 25) in the *C. elegans* genome in both samples. Inside each plot, N, RMSE, R2, and cor represent the number of bases, root-mean-square error, R^2 (coefficient of determination, where IPDs in the WGA and native samples were used as observation and prediction, respectively), and Pearson's correlation coefficient with the associated p-value. Dashed line is $y = x$. Note that values are shown using a logarithmic scale.

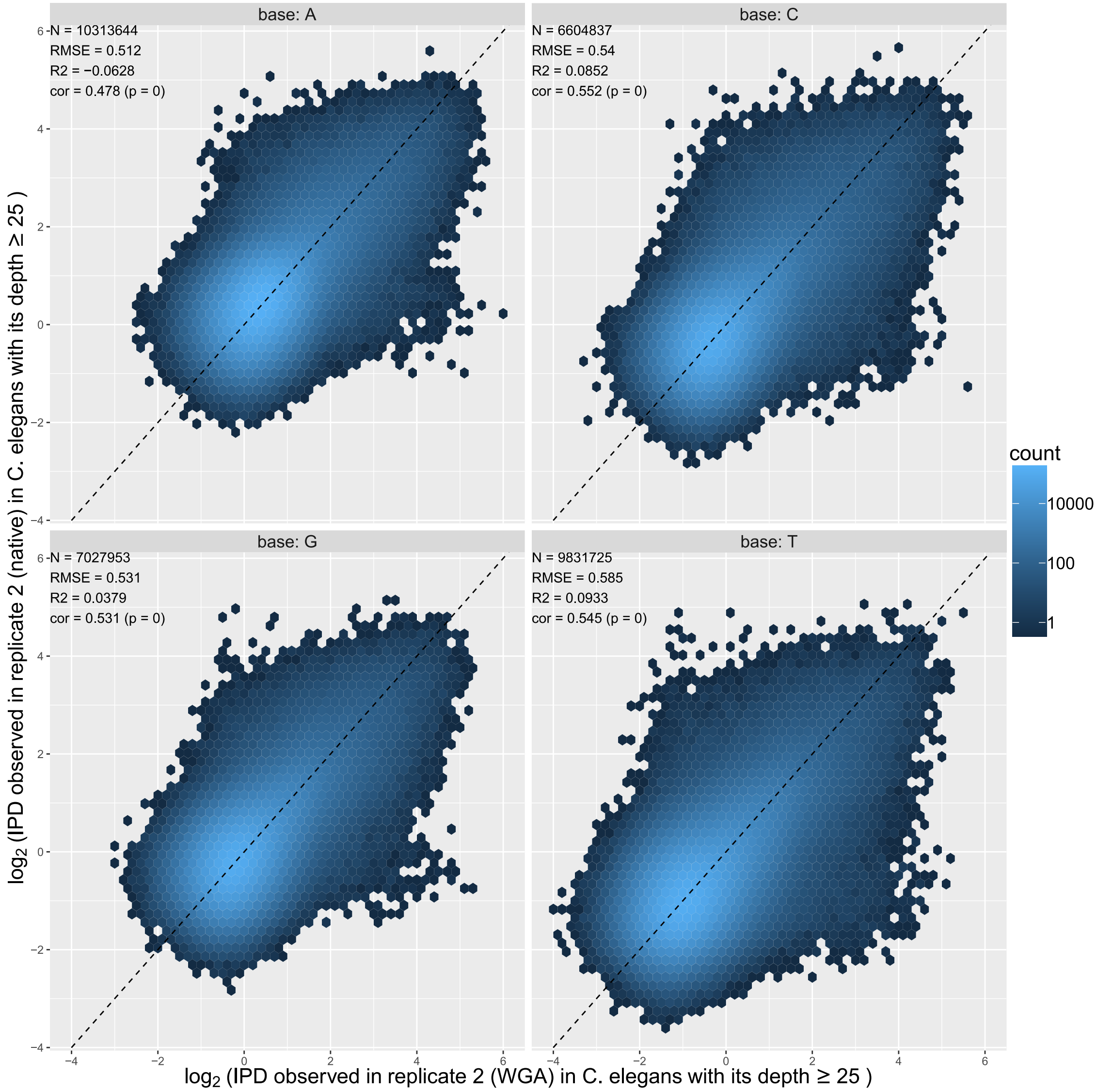

Supplementary Figure 3B: Hexbin plot similar to Supplementary Figure 3A that compares $\log_2$ (IPDs observed in replicate 2/WGA) with $\log_2$ (IPDs observed in replicate 2/native).

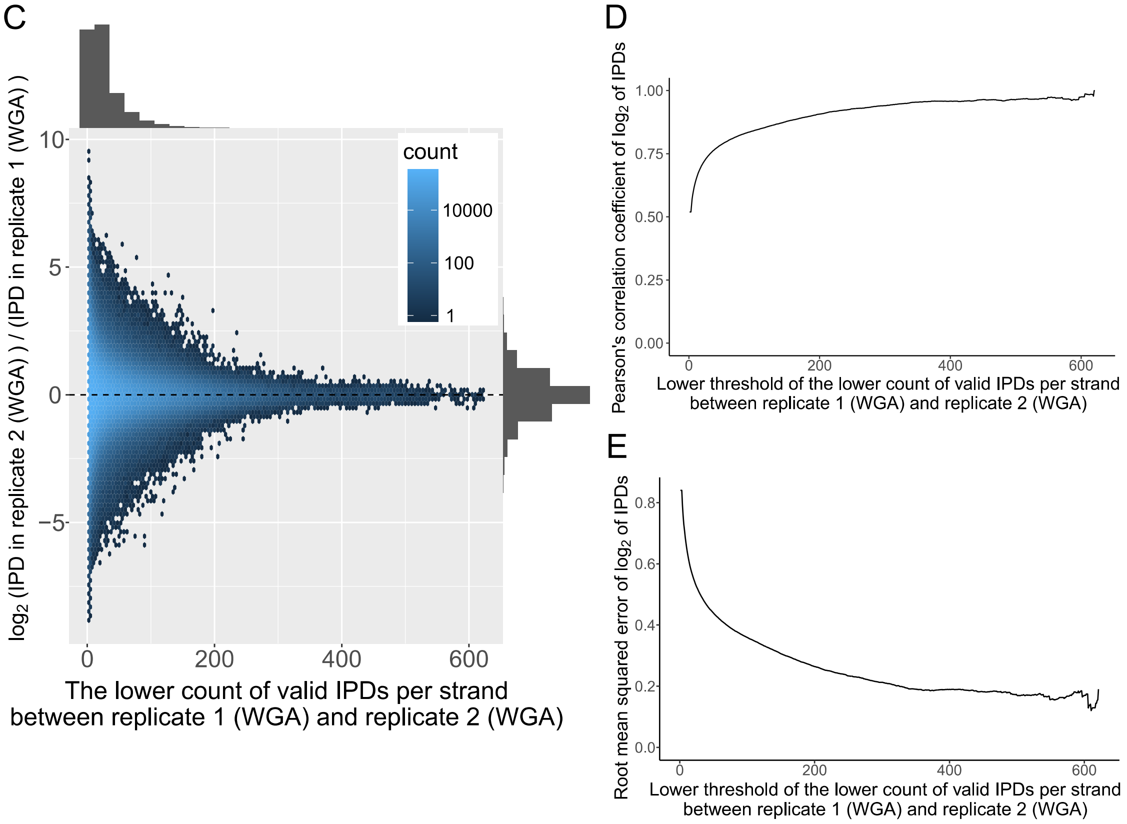

Supplementary Figure 3C, 3D, and 3E: Consistency in IPD values between two WGA samples (replicate 1/WGA and replicate 2/WGA). **(C)** Hexbin plot of $\log_2$ (IPD in replicate 2/WGA / IPD in replicate 1/WGA) versus lowest count (or depth) of valid IPDs (for each strand of replicate 1/WGA and replicate 2/WGA). Marginal histograms are also plotted. **(D)** Pearson's coefficient for $\log_2$ IPDs in replicate 1/WGA and replicate 2/WGA for variable thresholds of IPD lowest value. **(E)** Root-mean-square error of $\log_2$ IPDs in replicate 1/WGA and replicate 2/WGA for variable thresholds of IPD lowest value.

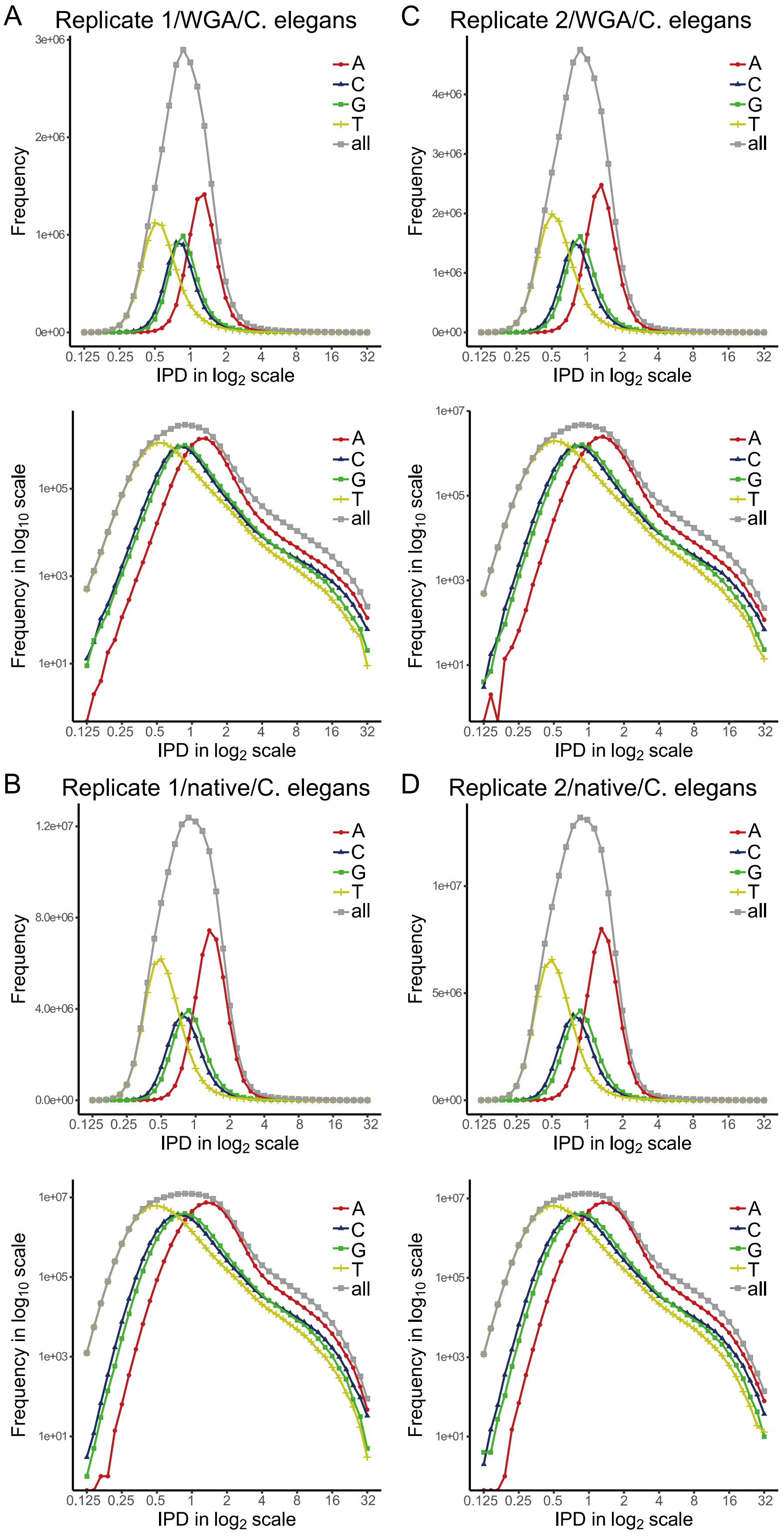
A
Replicate 1/WGA/C. elegans
C
Replicate 2/WGA/C. elegans
B
Replicate 1/native/C. elegans
D
Replicate 2/native/C. elegans
A
C
G
T
all
Frequency
Frequency in log10 scale
IPD in log2 scale
0.125 0.25 0.5 1 2 4 8 16 32
3e+06
2e+06
1e+06
0e+00
4e+06
1e+07
1e+05
1e+03
1e+01
1.2e+07
8.0e+06
4.0e+06
0.0e+00
5e+06

Supplementary Figure 4A-D: The frequency distributions of the IPDs of all bases (gray) and of individual four bases (adenine: red, cytosine: blue, guanine: green, thymine: yellow) that are valid and covered by $\geq$ 25 reads in the *C. elegans* genome (ce11, or WS235). **A** shows the IPD distributions in the replicate 1 (WGA) sample, **B** in the replicate 1 (native), **C** in the replicate 2 (WGA), and **D** in the replicate 2 (native). Each sample has two distributions, and in the lower distribution, the y-axis is in log-scale to highlight the frequencies of high IPDs $\geq$ 2.

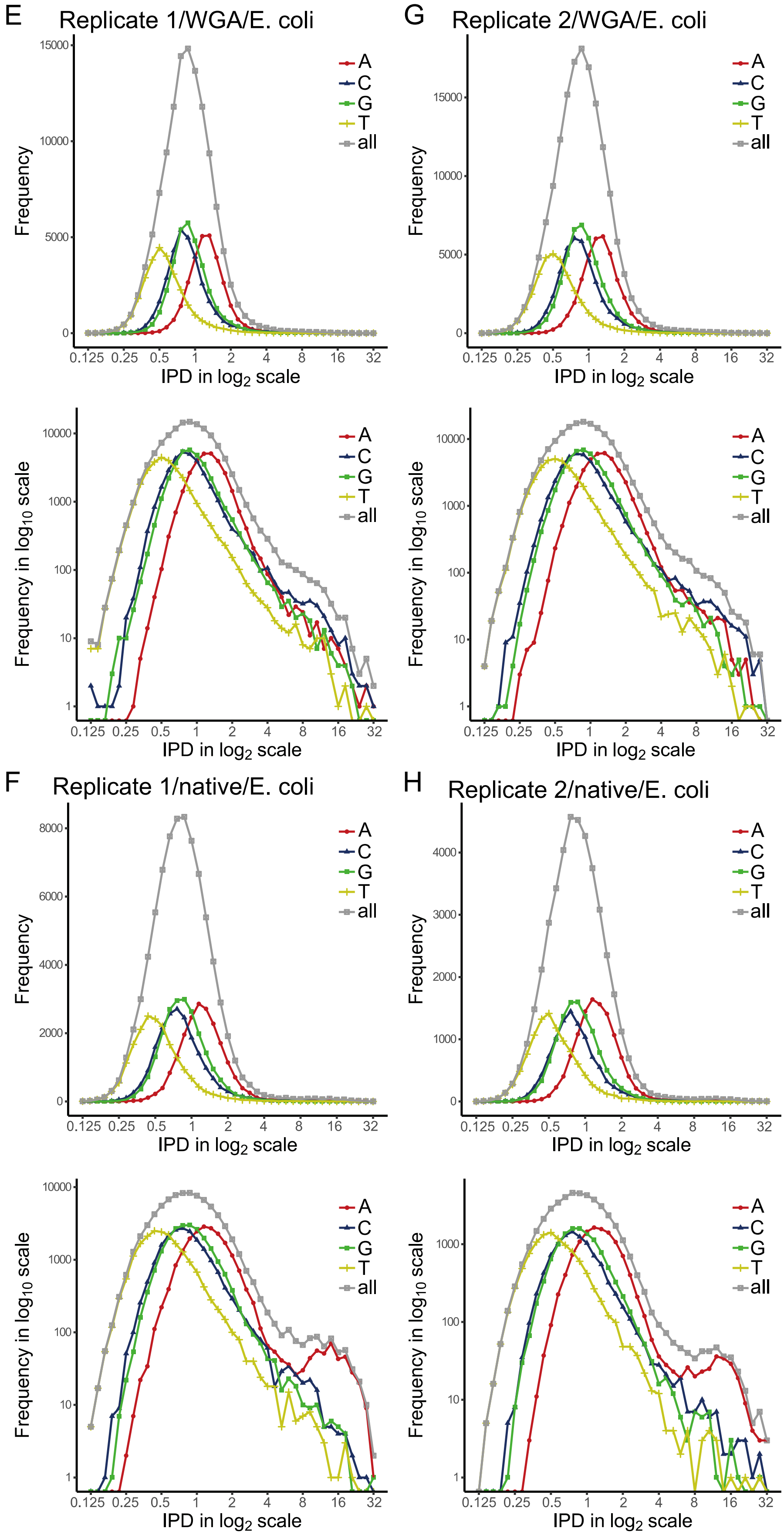
E
Replicate 1/WGA/E. coli
G
Replicate 2/WGA/E. coli
F
Replicate 1/native/E. coli
H
Replicate 2/native/E. coli
A
C
G
T
all
Frequency
Frequency in $\log_{10}$ scale
IPD in $\log_2$ scale
0.125
0.25
0.5
1
2
4
8
16
32

Supplementary Figure 4E-H: Figures show the frequency distributions of the IPDs of all bases (gray) and of individual four bases (adenine: red, cytosine: blue, guanine: green, thymine: yellow) that are valid and covered by $\geq$ 25 reads in the *E. coli* genome. **E** show the IPD distributions in the replicate 1 (WGA) sample, **F** in the replicate 1 (native), **G** in the replicate 2 (WGA), and **H** in the replicate 2 (native).

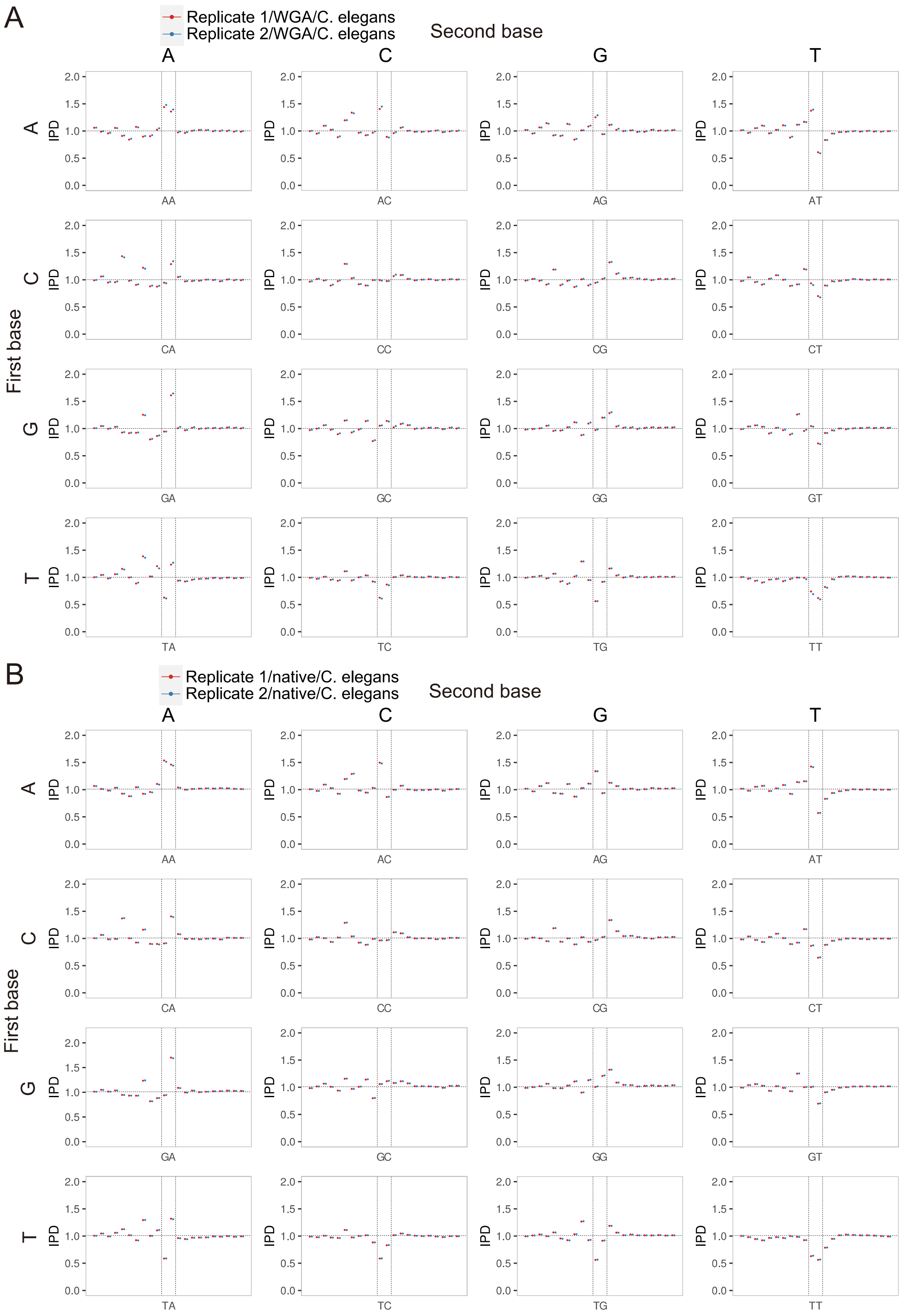

Supplementary Figure 5A and 5B: A and **B** respectively show concordance among the IPDs of the <u>*C. elegance*</u> genome in the two WGA replicates and in the two native replicates for each of 2-mer motifs.

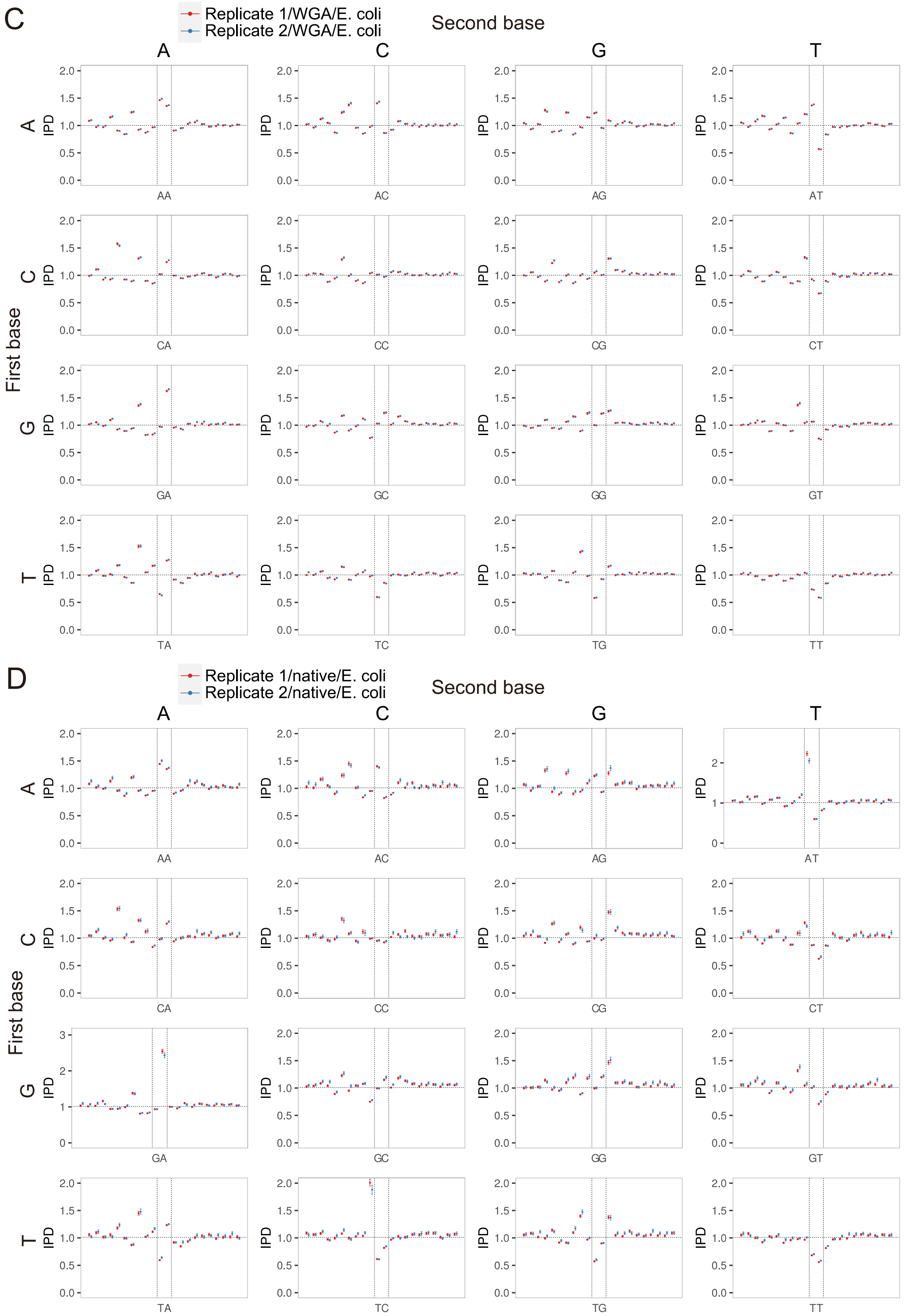

Supplementary Figure 5C and 5D: Similarly to Supplementary Figure 5A and 5B, Figures **C** and **D** respectively show concordance among the IPDs of the <u>*E. coli*</u> genome in place of the *C. elegans* genome in the two WGA replicates and in the two native replicates for each of 2-mer motifs.

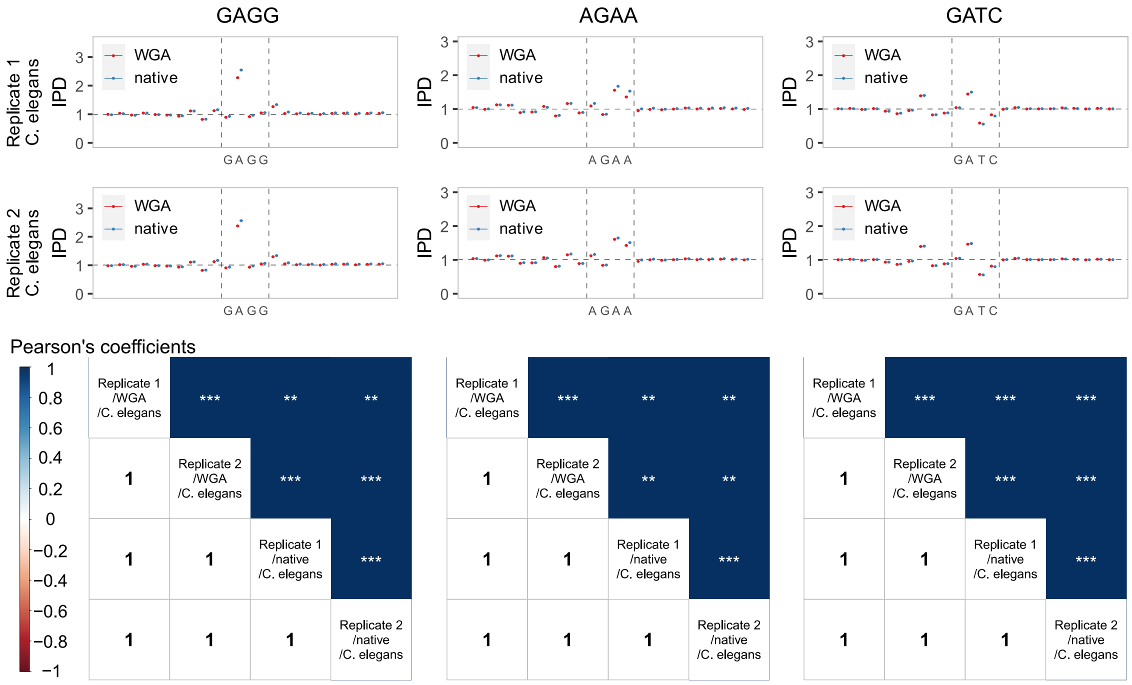

Supplementary Figure 6A: Similar to Figure 2, the top two rows show the concordance among the IPDs in the WGA (colored red) and native (blue) samples of replicate 1 and replicate 2 for each of three motifs. The bottom row displays Pearson's coefficients showing correlations of mean values of $\log_2$ IPDs within individual motifs between pairs of the four samples (replicate 1/WGA, replicate 2/WGA, replicate 1/native, and replicate 2/native) observed in the *C. elegans*. Correlation coefficients are shown with colors in upper triangle and with numbers in lower triangle.

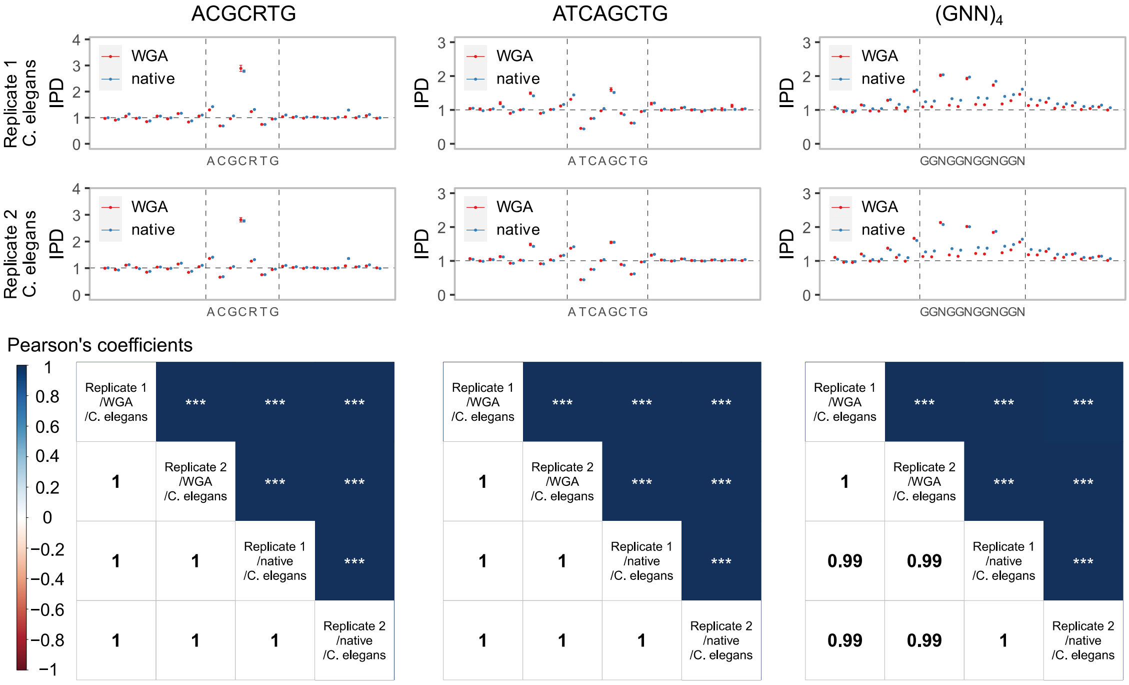

Supplementary Figure 6B: Similar to Supplementary Figure 6A, we show the concordance between the IPDs in the four samples (replicate 1/WGA, replicate 2/WGA, replicate 1/native, and replicate 2/native) for each of three motifs with extreme IPDs.

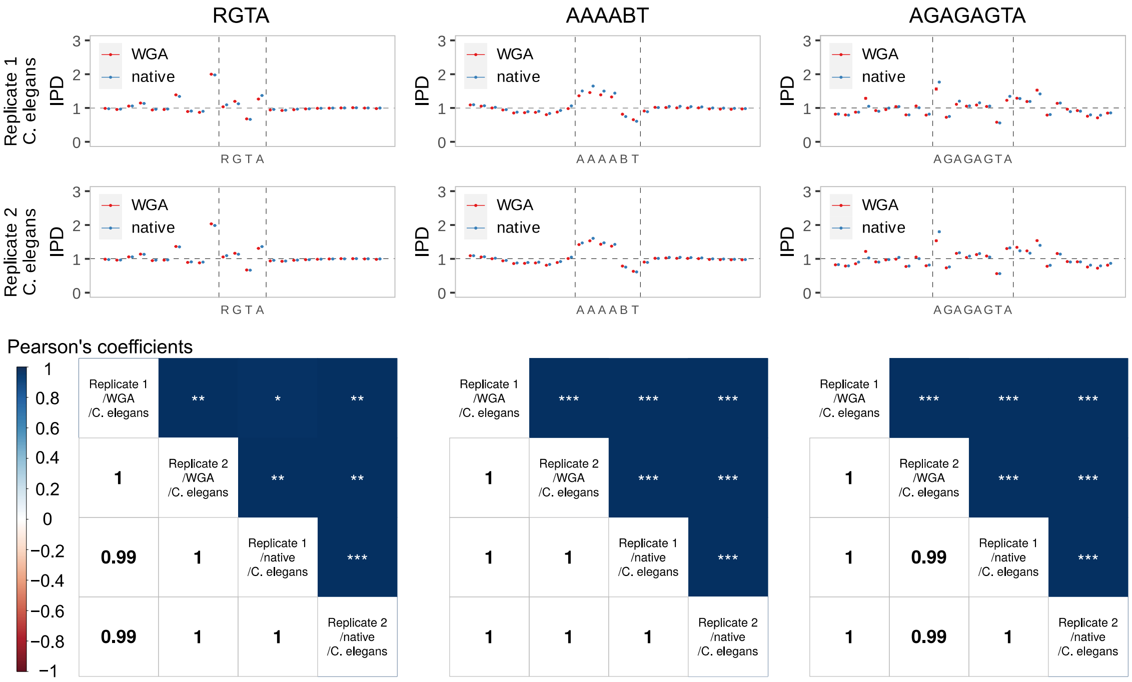

Supplementary Figure 6C: Similar to Supplementary Figure 6A, we show the concordance between the IPDs in the four samples (replicate 1/WGA, replicate 2/WGA, replicate 1/native, and replicate 2/native) for each of three motifs with extreme IPDs.

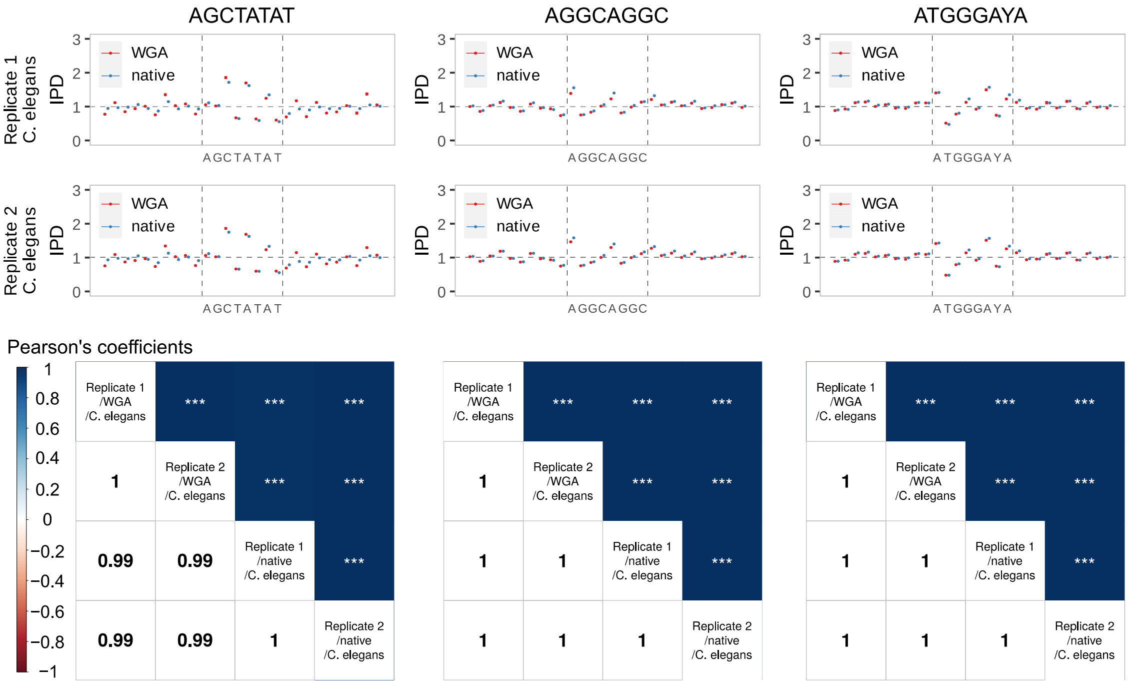

Supplementary Figure 6D: Similar to Supplementary Figure 6A, we show the concordance between the IPDs in the four samples (replicate 1/WGA, replicate 2/WGA, replicate 1/native, and replicate 2/native) for each of three motifs with extreme IPDs.

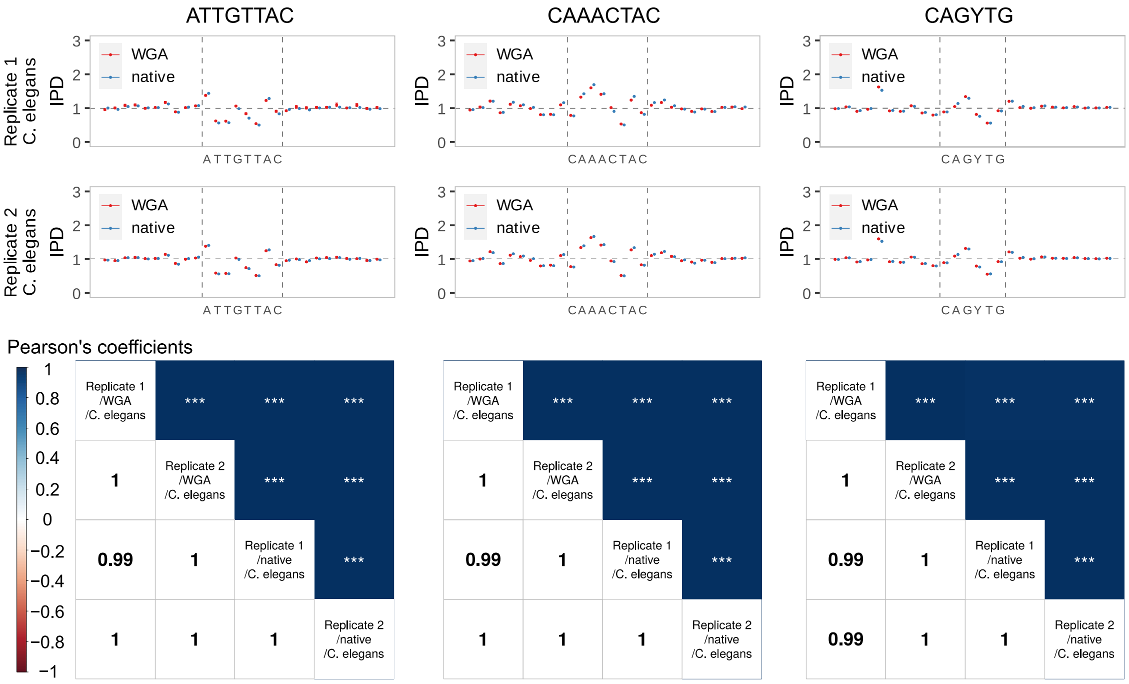

Supplementary Figure 6E: Similar to Supplementary Figure 6A, we show the concordance between the IPDs in the four samples (replicate 1/WGA, replicate 2/WGA, replicate 1/native, and replicate 2/native) for each of three motifs with extreme IPDs.

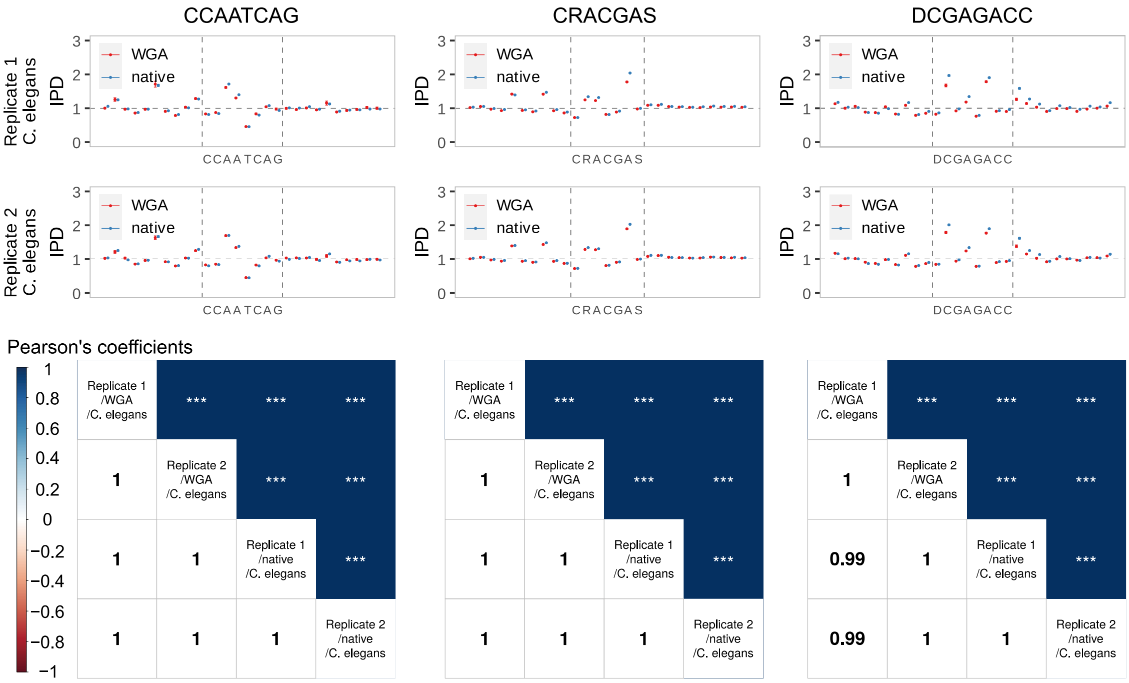

Supplementary Figure 6F: Similar to Supplementary Figure 6A, we show the concordance between the IPDs in the four samples (replicate 1/WGA, replicate 2/WGA, replicate 1/native, and replicate 2/native) for each of three motifs with extreme IPDs.

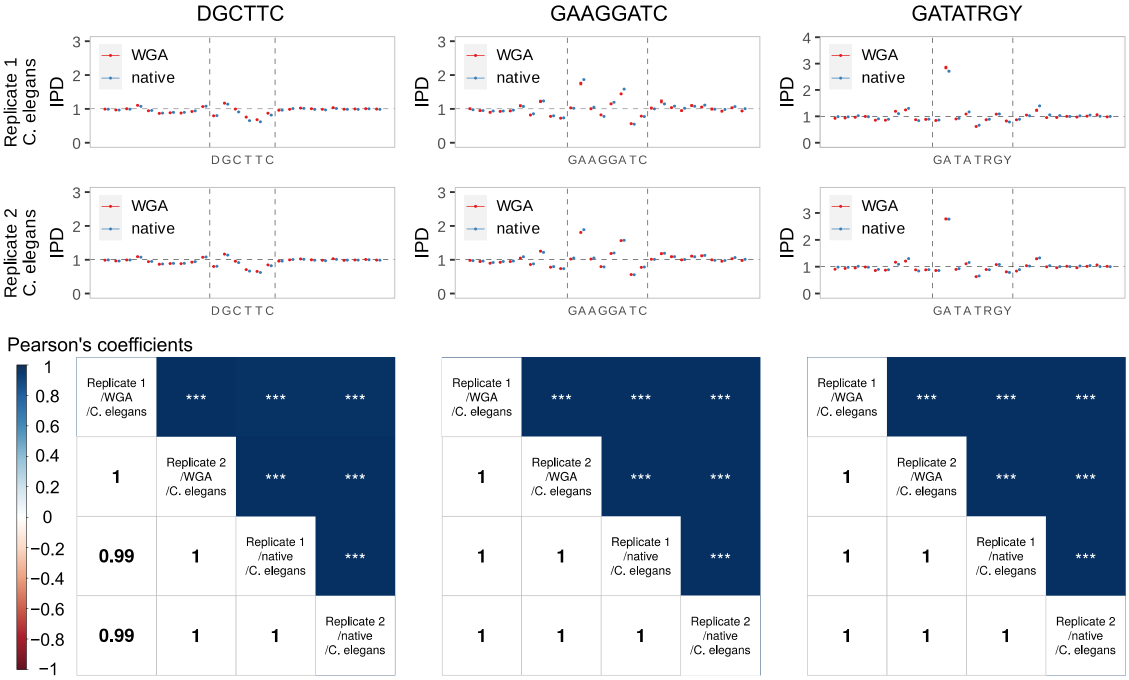

Supplementary Figure 6G: Similar to Supplementary Figure 6A, we show the concordance between the IPDs in the four samples (replicate 1/WGA, replicate 2/WGA, replicate 1/native, and replicate 2/native) for each of three motifs with extreme IPDs.

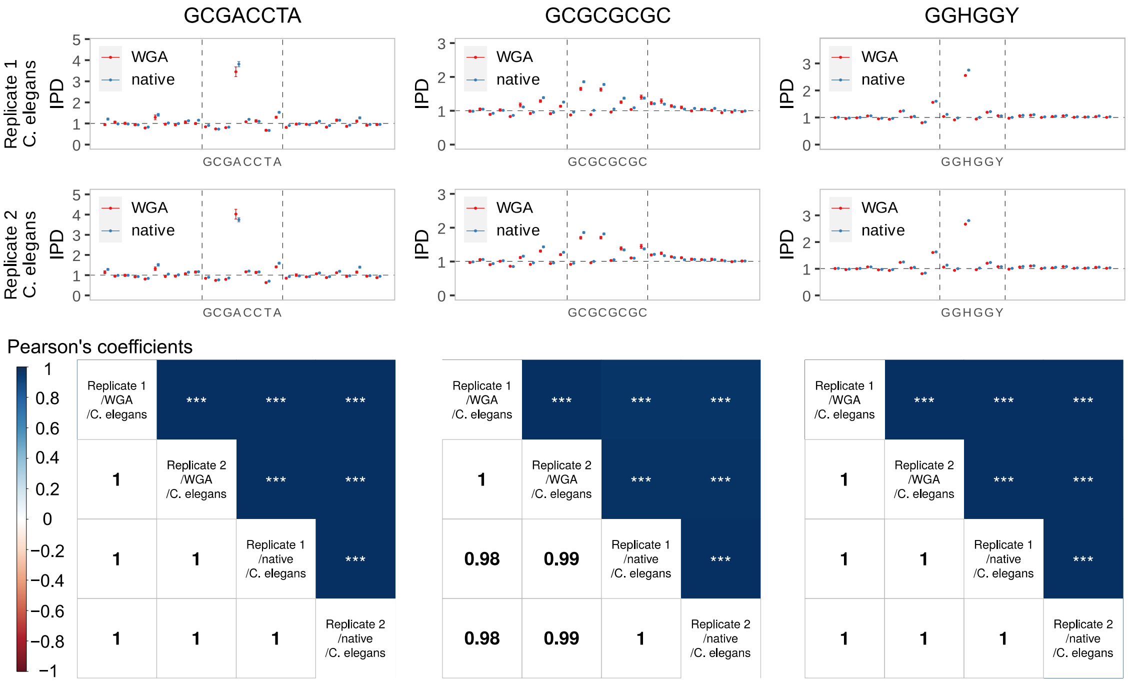

Supplementary Figure 6H: Similar to Supplementary Figure 6A, we show the concordance between the IPDs in the four samples (replicate 1/WGA, replicate 2/WGA, replicate 1/native, and replicate 2/native) for each of three motifs with extreme IPDs.

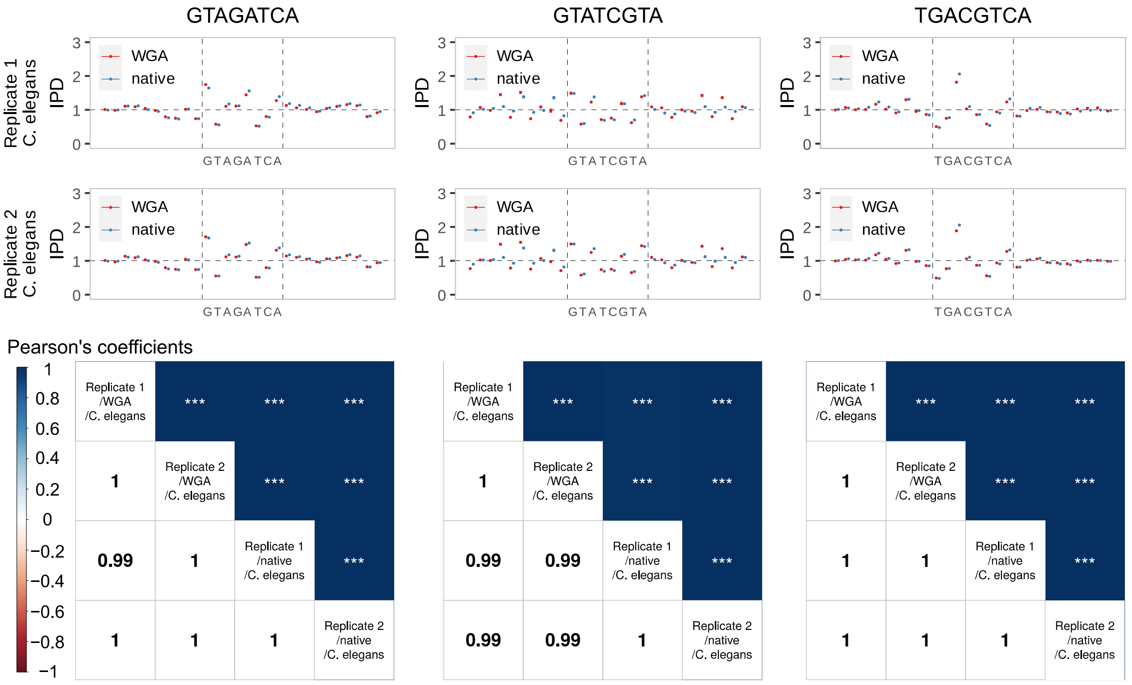

Supplementary Figure 6I: Similar to Supplementary Figure 6A, we show the concordance between the IPDs in the four samples (replicate 1/WGA, replicate 2/WGA, replicate 1/native, and replicate 2/native) for each of three motifs with extreme IPDs.

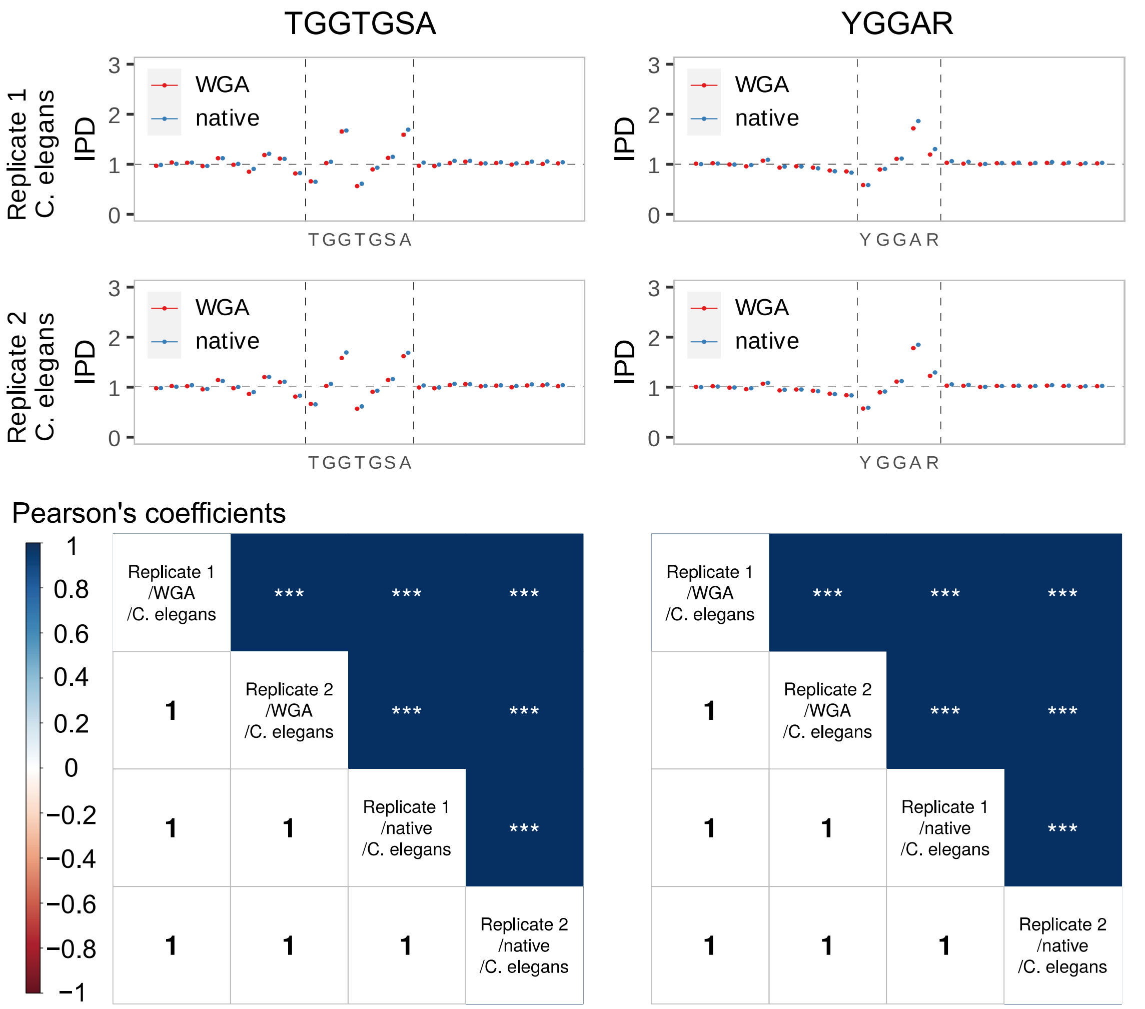

Supplementary Figure 6J: Similar to Supplementary Figure 6A, we show the concordance between the IPDs in the four samples (replicate 1/WGA, replicate 2/WGA, replicate 1/native, and replicate 2/native) for each of two motifs with extreme IPDs.

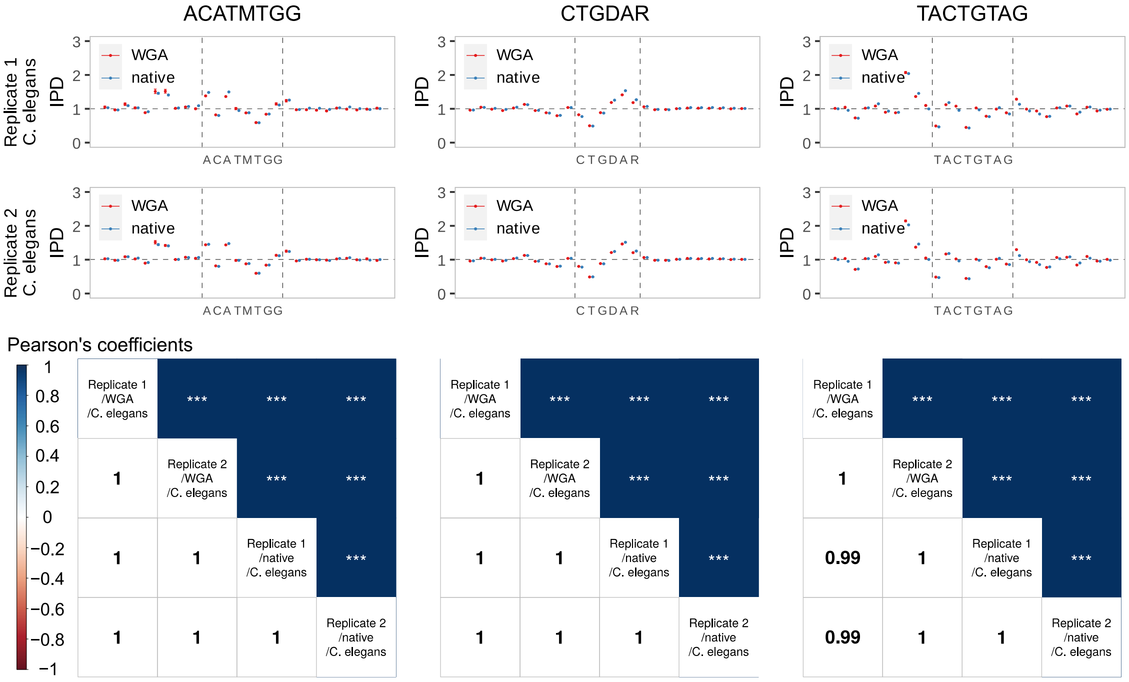

Supplementary Figure 6K: Similar to Supplementary Figure 6A, we show the concordance between the IPDs in the four samples (replicate 1/WGA, replicate 2/WGA, replicate 1/native, and replicate 2/native) for each of three motifs with extreme IPDs.

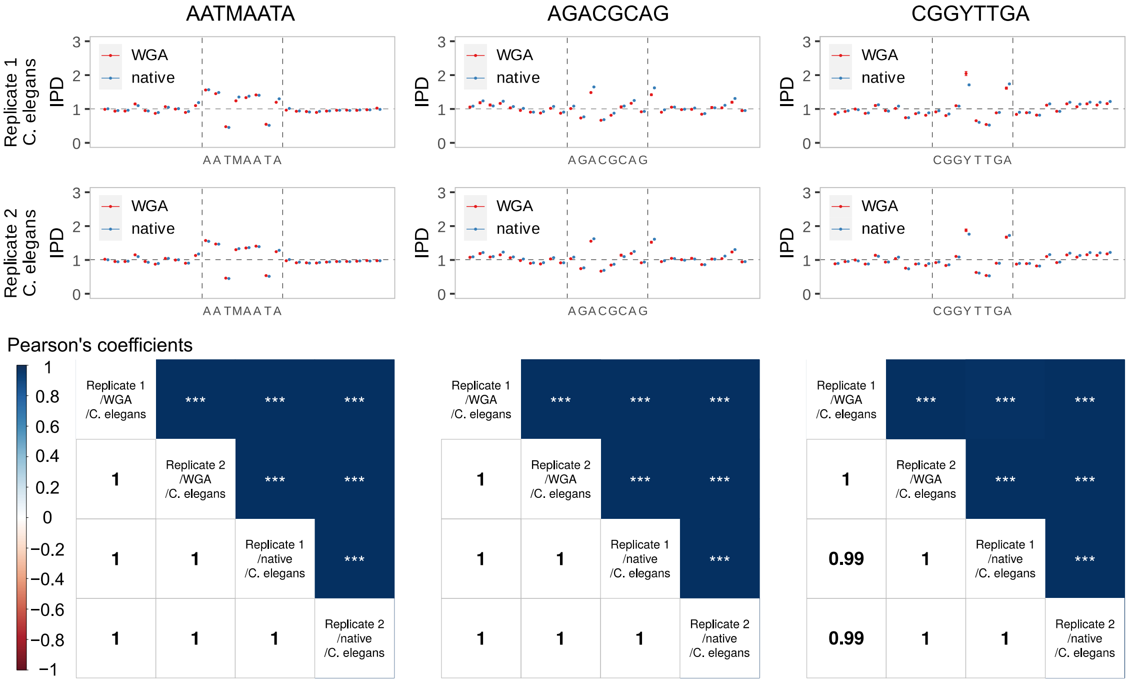

Supplementary Figure 6L: Similar to Supplementary Figure 6A, we show the concordance between the IPDs in the four samples (replicate 1/WGA, replicate 2/WGA, replicate 1/native, and replicate 2/native) for each of three motifs with extreme IPDs.

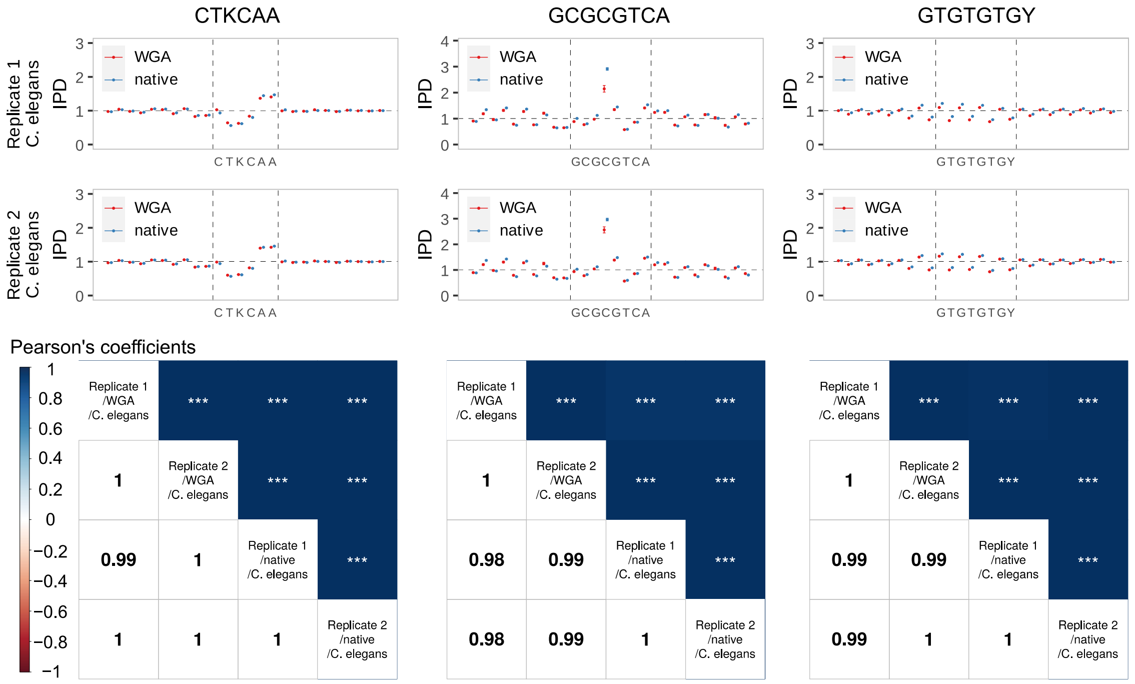

Supplementary Figure 6M: Similar to Supplementary Figure 6A, we show the concordance between the IPDs in the four samples (replicate 1/WGA, replicate 2/WGA, replicate 1/native, and replicate 2/native) for each of three motifs with extreme IPDs.

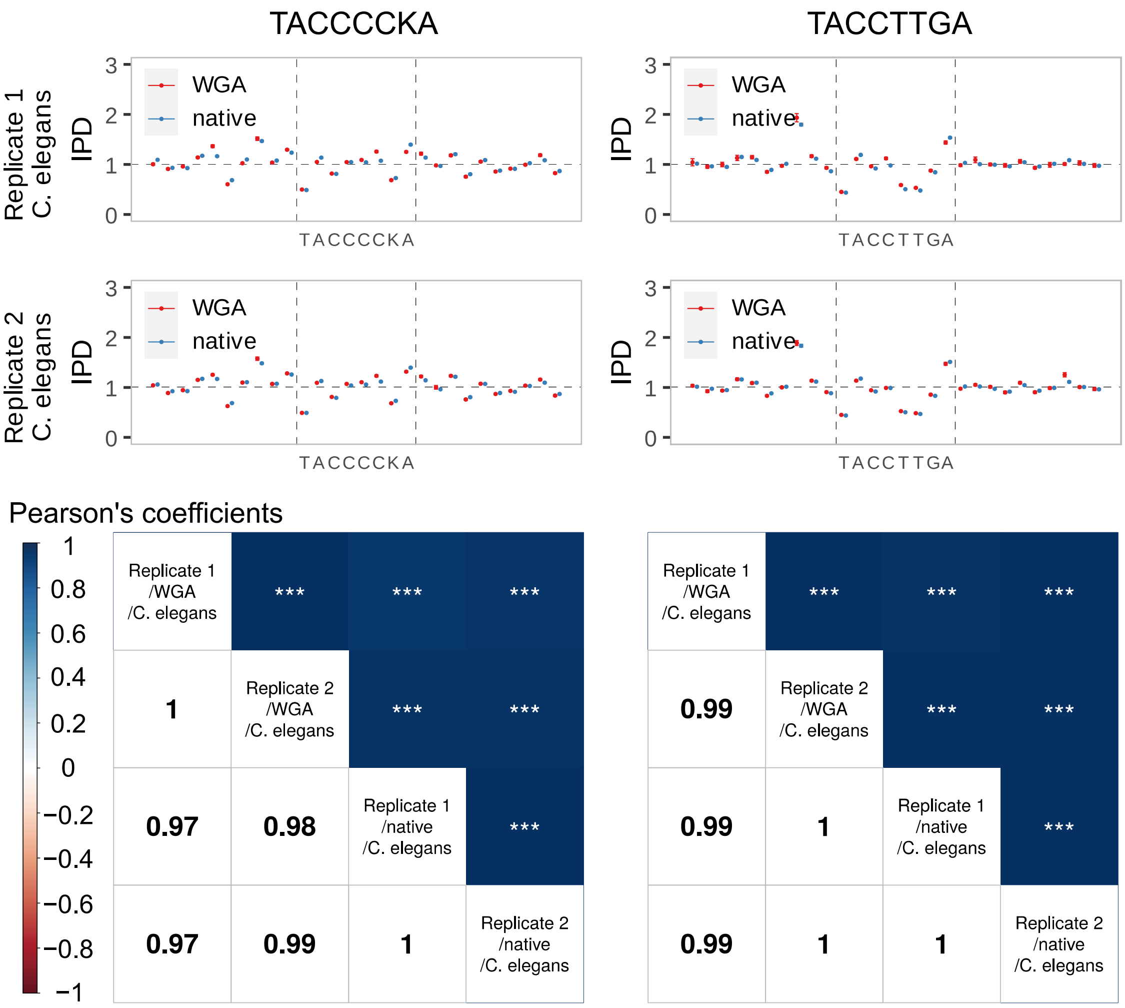

Supplementary Figure 6N: Similar to Supplementary Figure 6A, we show the concordance between the IPDs in the four samples (replicate 1/WGA, replicate 2/WGA, replicate 1/native, and replicate 2/native) for each of two motifs with extreme IPDs.

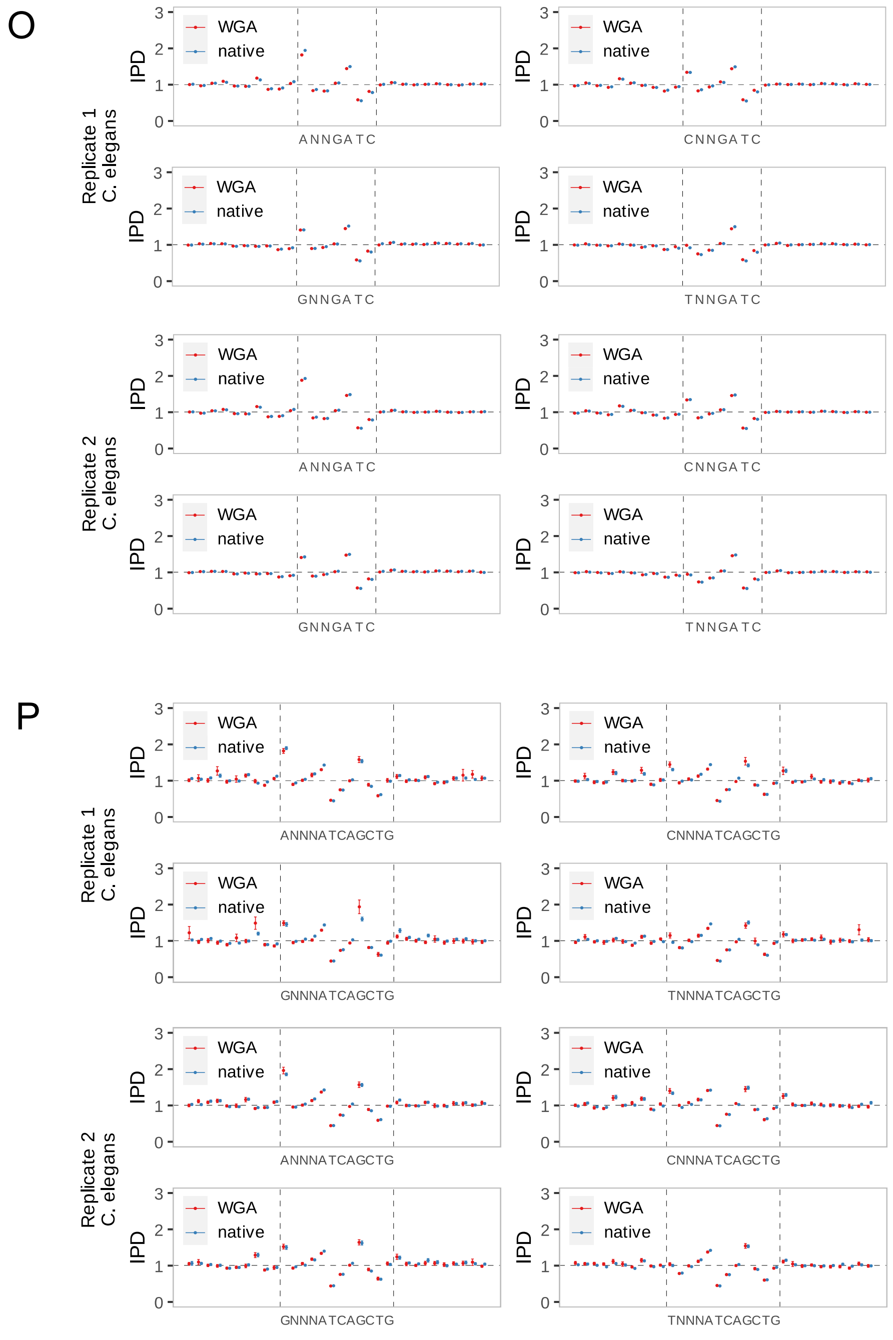

Supplementary Figure 6O and 6P: (O) The upper graphs show the IPD distributions of motif GATC with four nucleotides at the position three bases upstream of the motif, say ANNGATC, on the *C. elegans* genome in the four samples, replicate 1/WGA (red) and replicate 1/native (blue) in the first row, and replicate 2/WGA (red) and replicate 2/native (blue) in the second row. **(P)** Similar to Figure O, the IPD distributions around motif ATCAGCTG with four nucleotides four bases upstream of the motif.

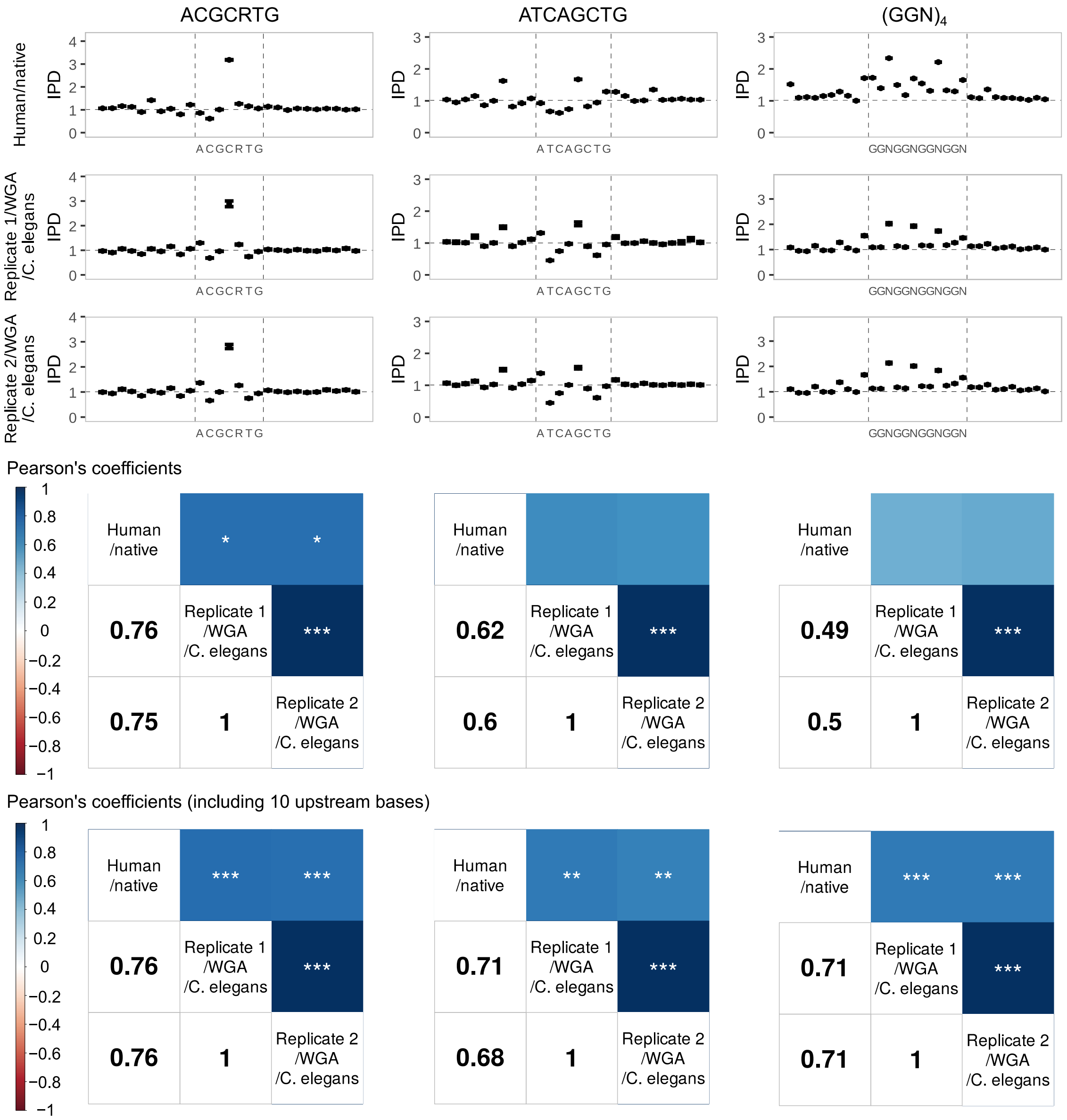

Supplementary Figure 7A: The top three rows show the IPD distributions represented by an error bar plot in the motifs and their surrounding 10 nucleotides in the human sample (the first row), the *C. elegans* replicate 1/WGA (the second row), and the *C. elegans* replicate 2/WGA (the third row). The fourth and fifth rows show Pearson's correlation coefficients of mean values of log2 IPDs within individual motifs (the fourth row) or within individual motifs and the 10 nucleotides in their upstream regions (the fifth row) between pairs of the three samples. The 10 nucleotides upstream of the motifs are considered because they are likely to have nucleotides with significantly high or low IPD values. Correlation coefficients are shown with colors in upper triangle and with numbers in lower triangle.

Statistical significance is shown in upper triangle as follows. (*): $p \leq 0.05$, (**): $p \leq 0.01$, (***): $p \leq 0.001$.

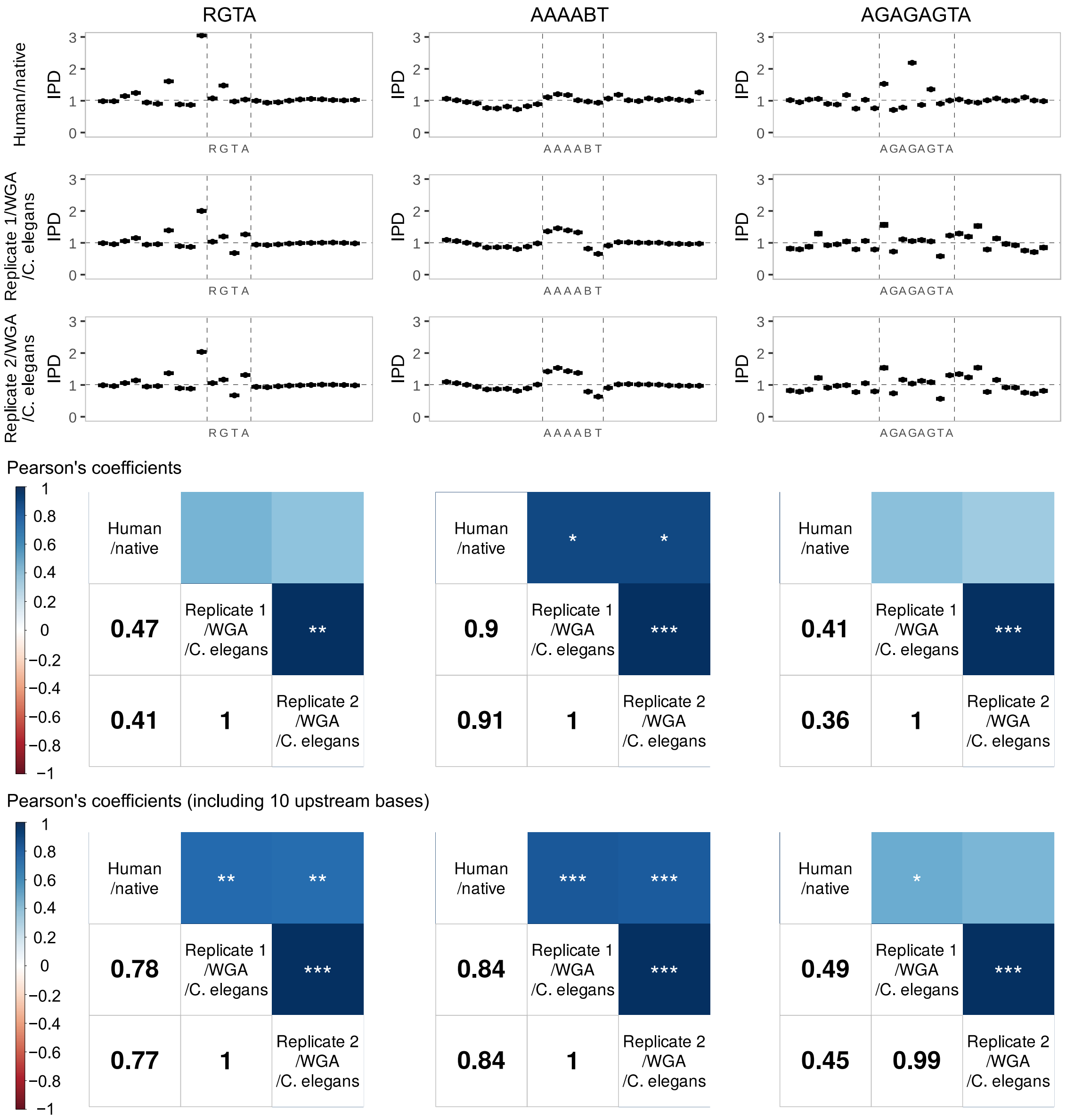

Supplementary Figure 7B: Similar to Supplementary Figure 7A, we show the IPD distributions and correlation coefficients in the three samples for each motif with extreme IPDs.

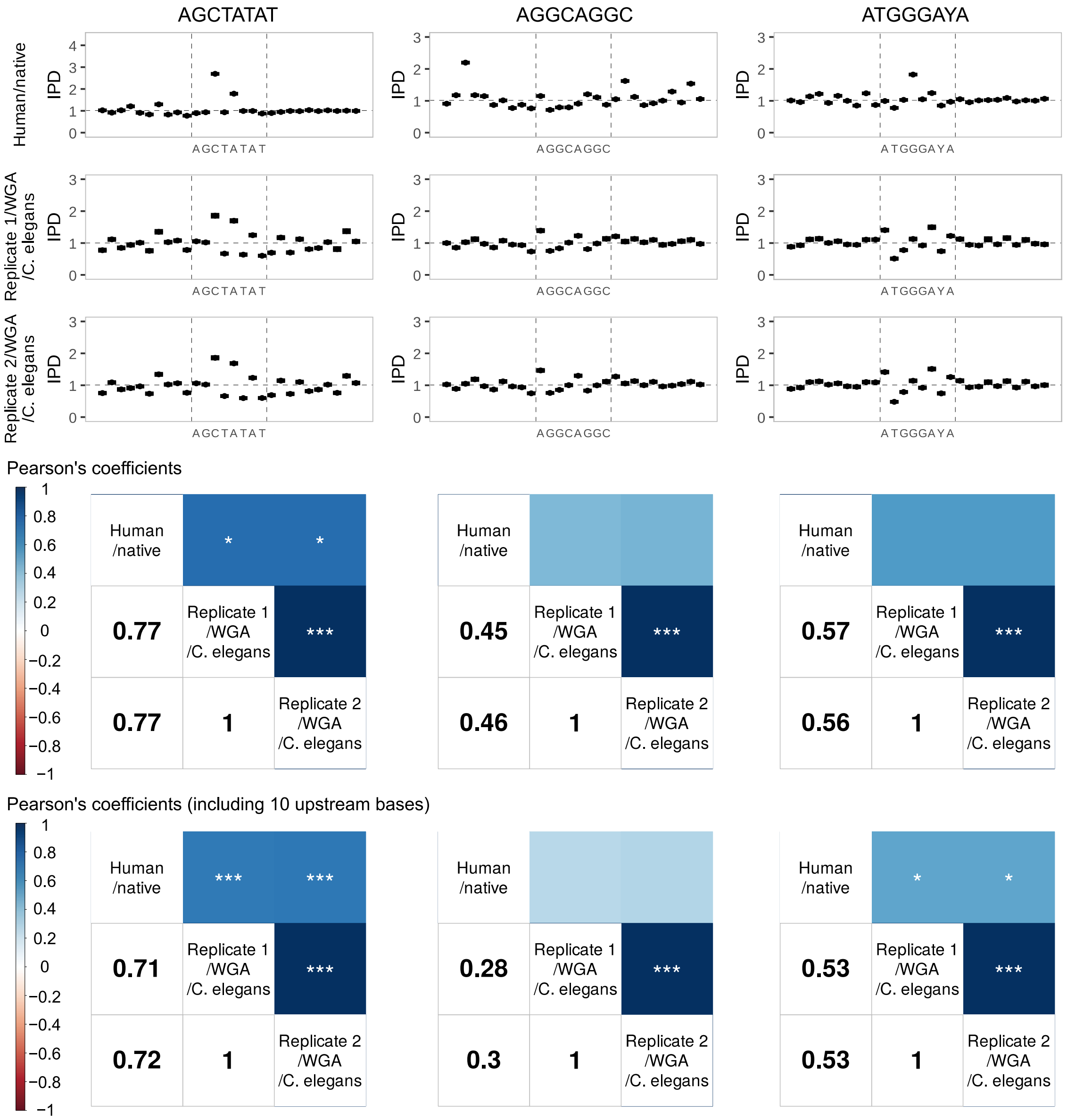

Supplementary Figure 7C: Similar to Supplementary Figure 7A, we show the IPD distributions and correlation coefficients in the three samples for each motif with extreme IPDs.

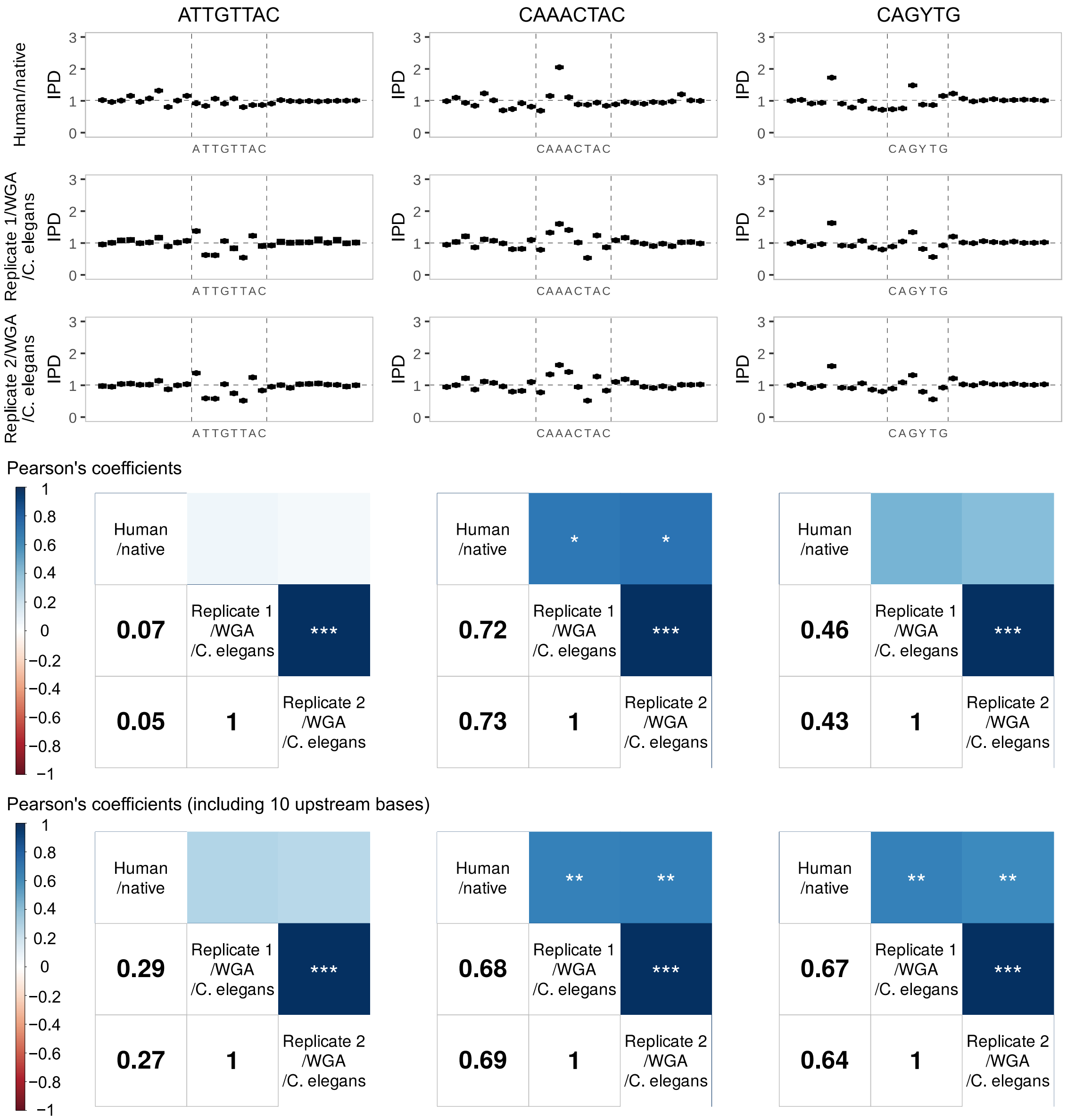

Supplementary Figure 7D: Similar to Supplementary Figure 7A, we show the IPD distributions and correlation coefficients in the three samples for each motif with extreme IPDs.

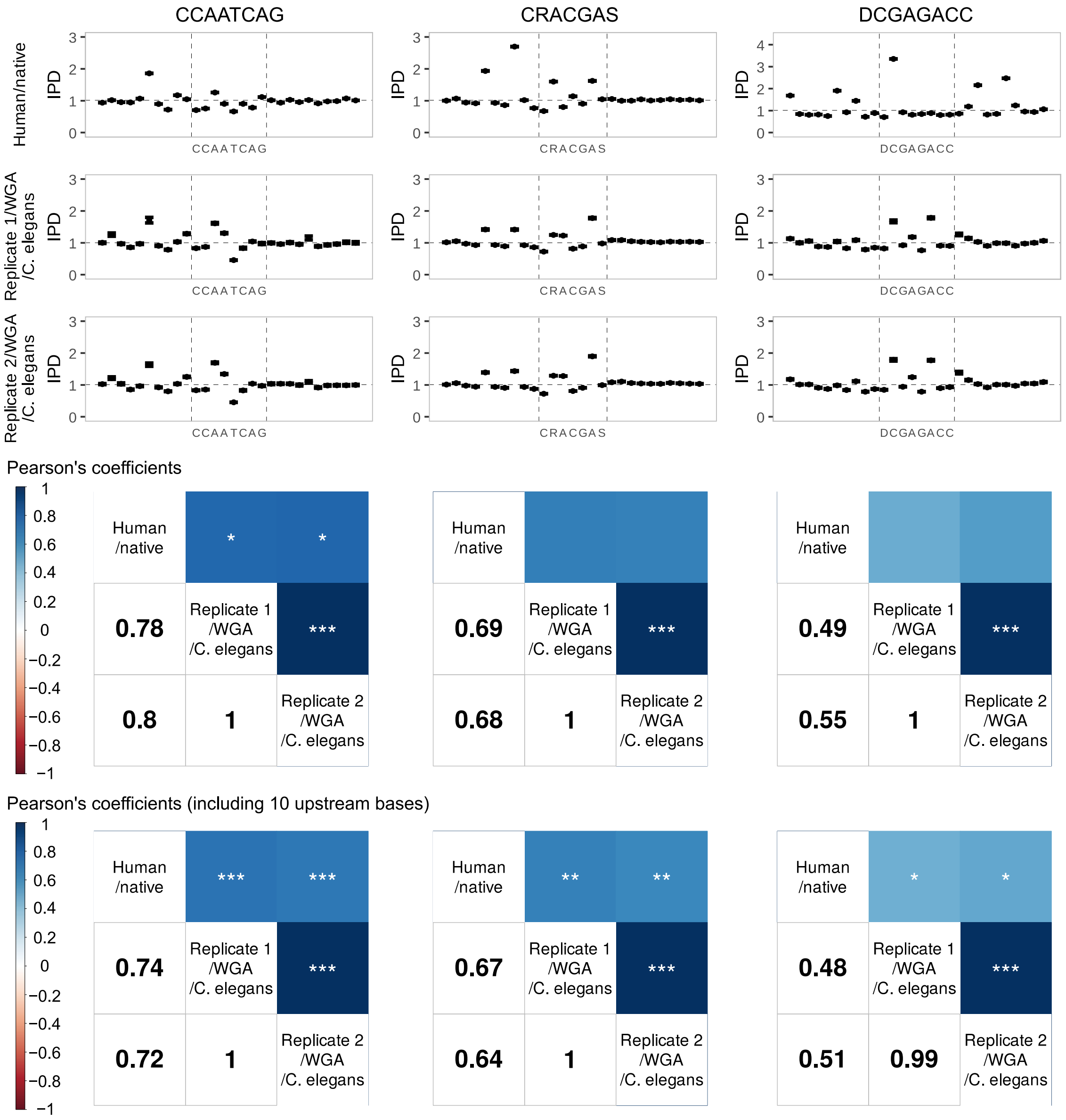

Supplementary Figure 7E: Similar to Supplementary Figure 7A, we show the IPD distributions and correlation coefficients in the three samples for each motif with extreme IPDs.

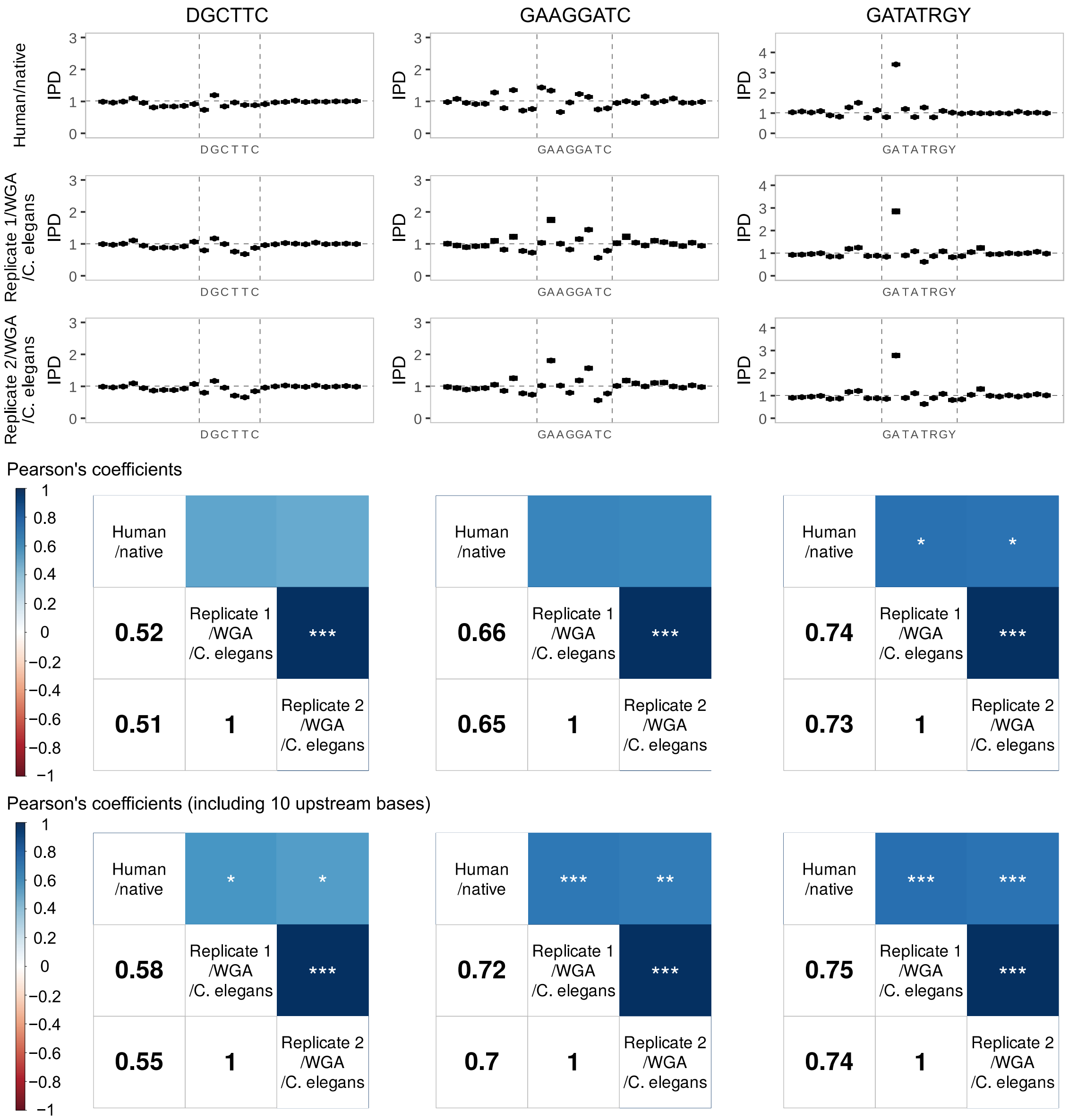

Supplementary Figure 7F: Similar to Supplementary Figure 7A, we show the IPD distributions and correlation coefficients in the three samples for each motif with extreme IPDs.

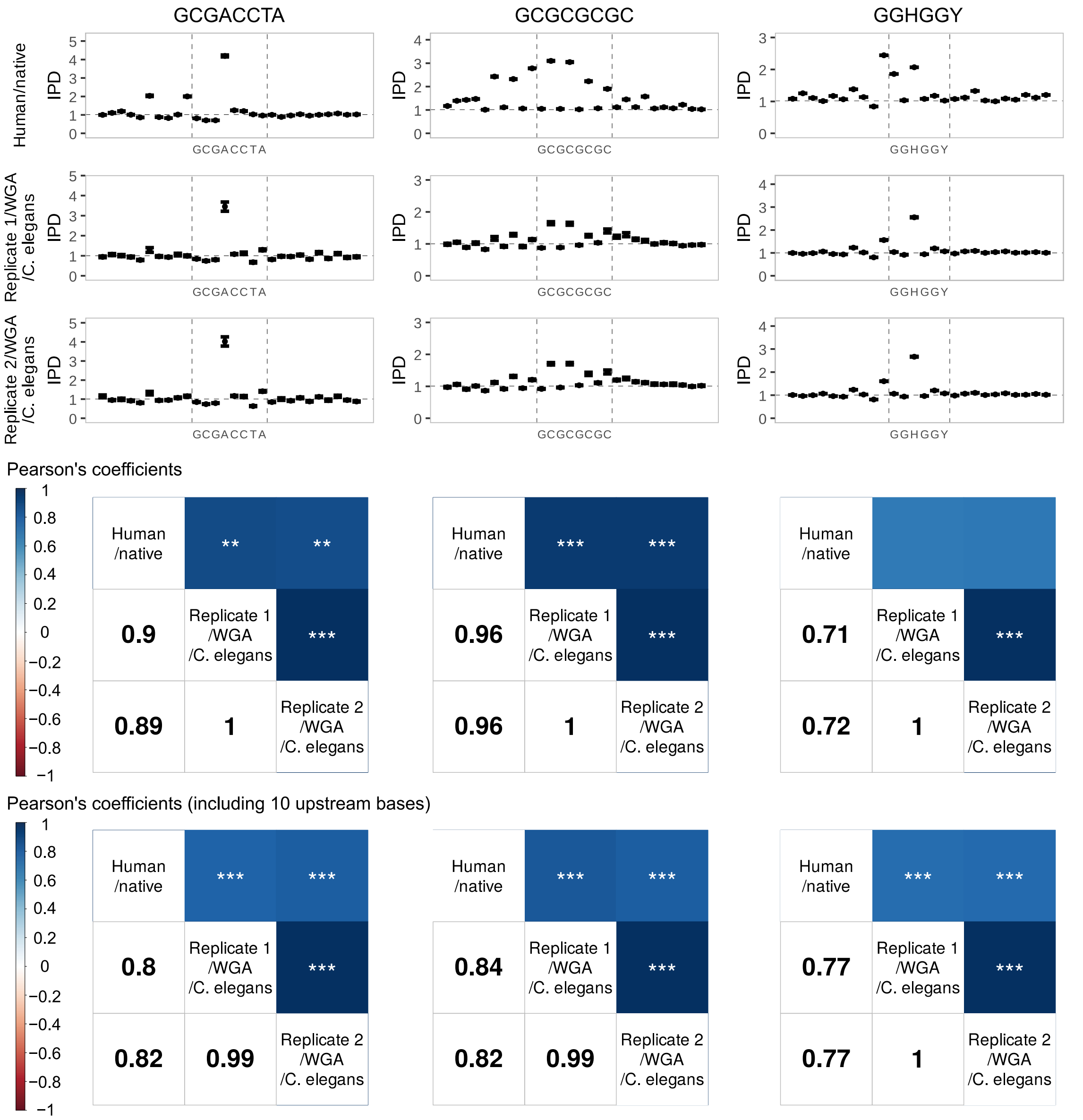

Supplementary Figure 7G: Similar to Supplementary Figure 7A, we show the IPD distributions and correlation coefficients in the three samples for each motif with extreme IPDs.

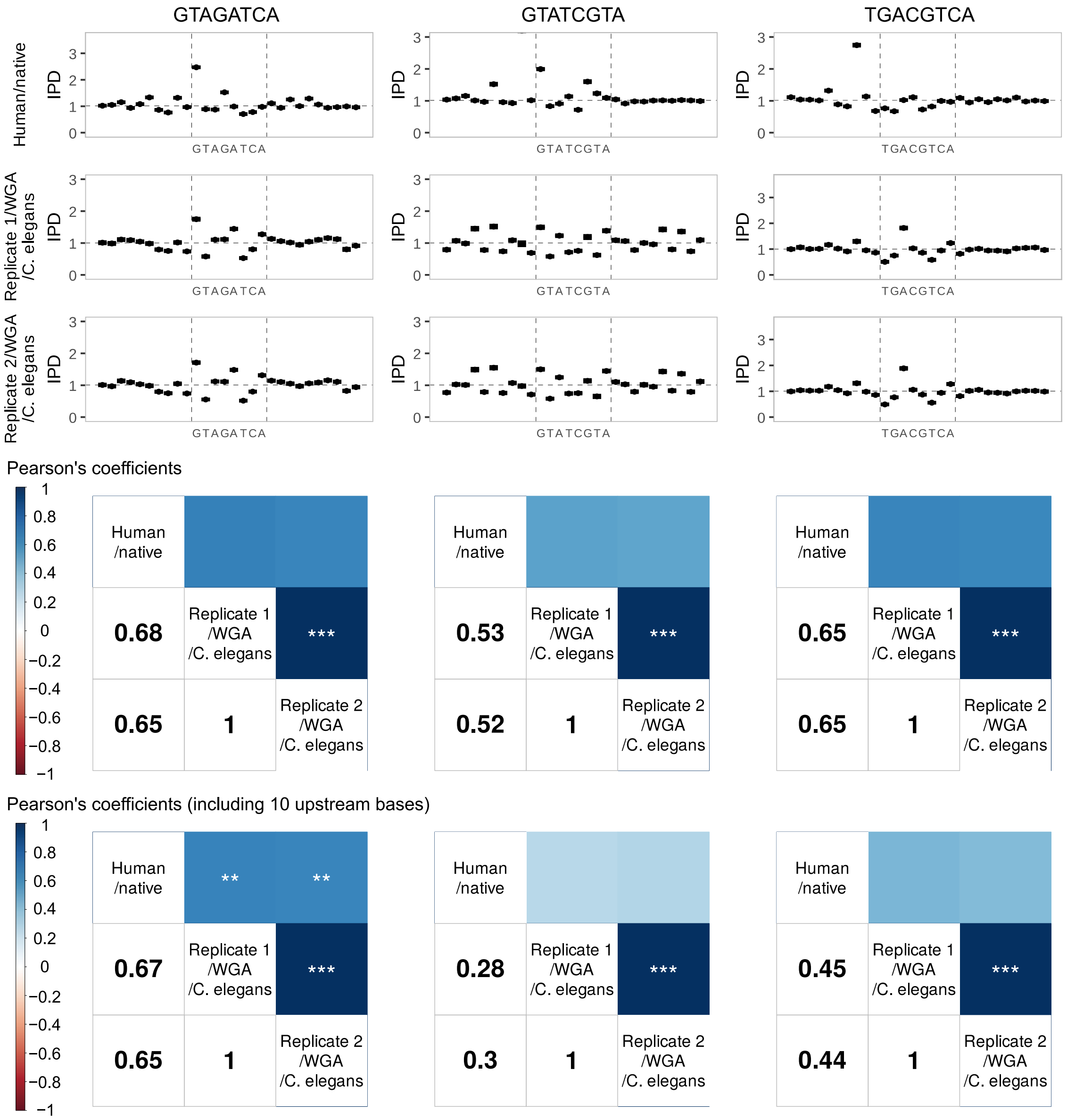

Supplementary Figure 7H: Similar to Supplementary Figure 7A, we show the IPD distributions and correlation coefficients in the three samples for each motif with extreme IPDs.

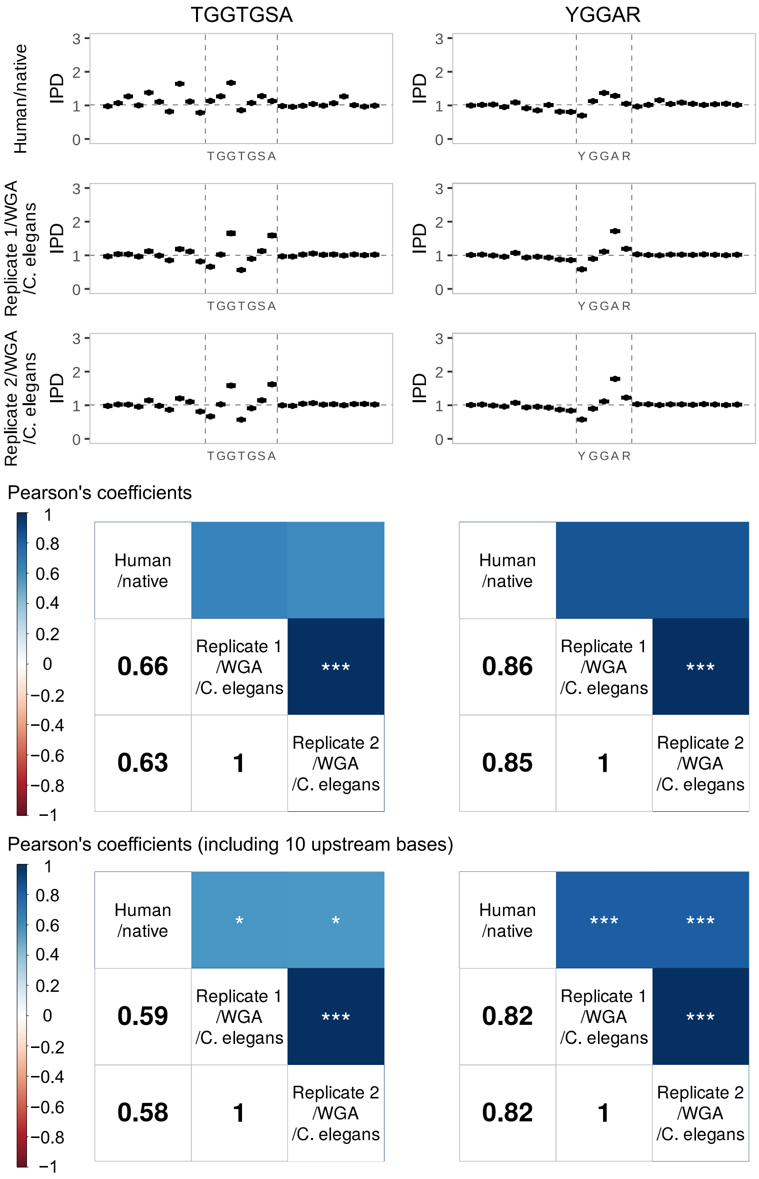

Supplementary Figure 7I: Similar to Supplementary Figure 7A, we show the IPD distributions and correlation coefficients in the three samples for each motif with extreme IPDs.

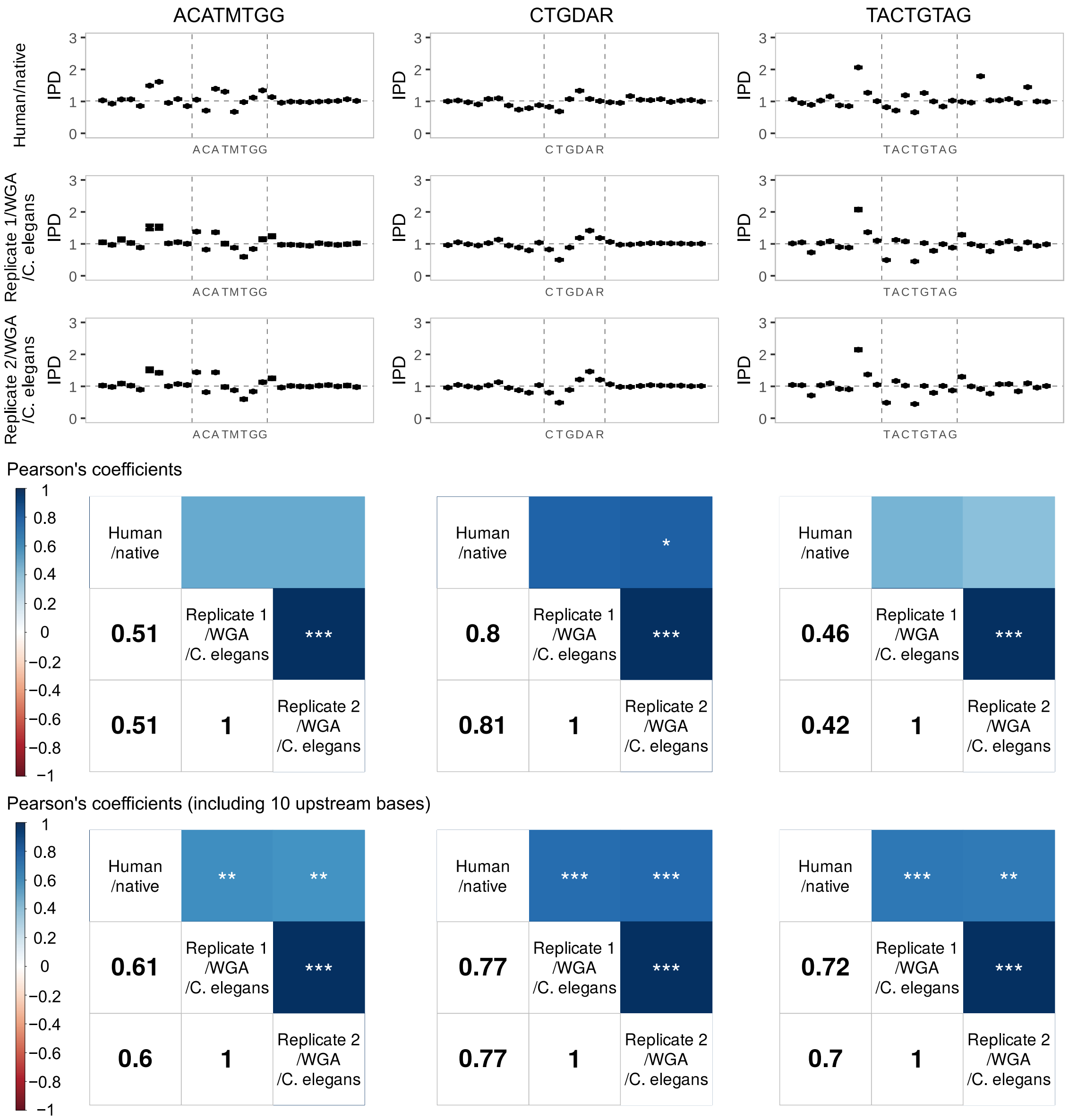

Supplementary Figure 7J: Similar to Supplementary Figure 7A, we show the IPD distributions and correlation coefficients in the three samples for each motif with extreme IPDs.

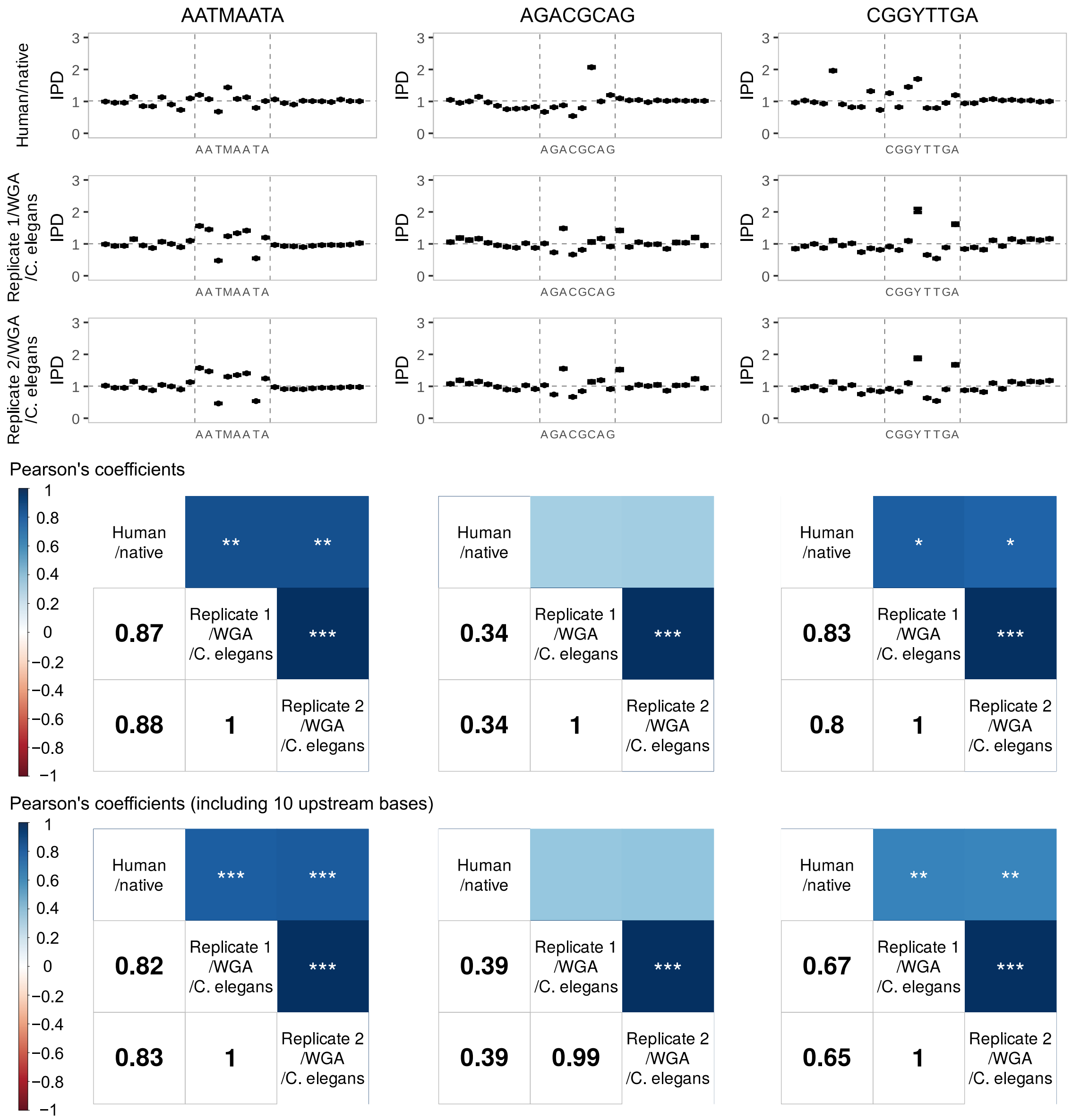

Supplementary Figure 7K: Similar to Supplementary Figure 7A, we show the IPD distributions and correlation coefficients in the three samples for each motif with extreme IPDs.

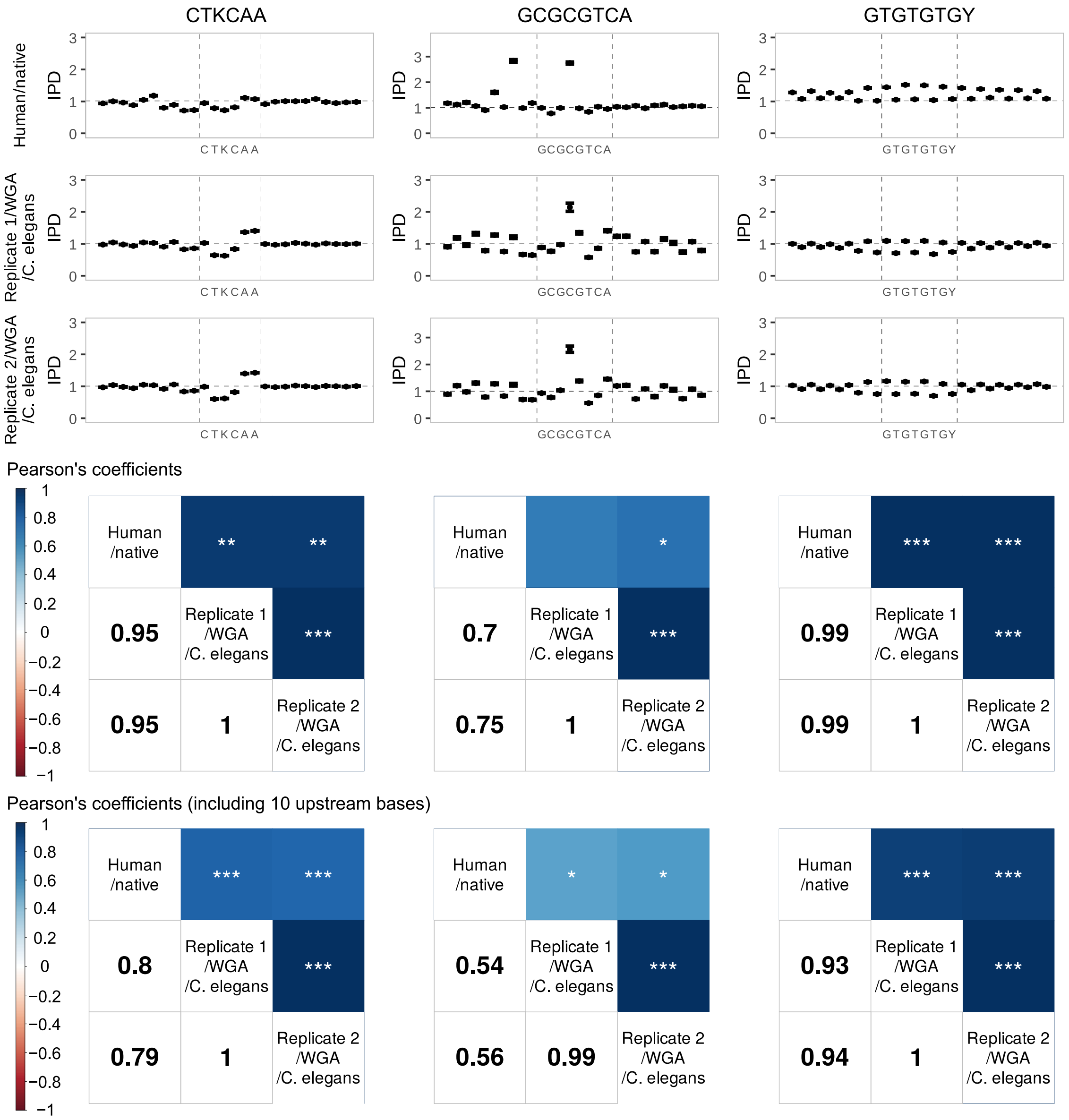

Supplementary Figure 7L: Similar to Supplementary Figure 7A, we show the IPD distributions and correlation coefficients in the three samples for each motif with extreme IPDs.

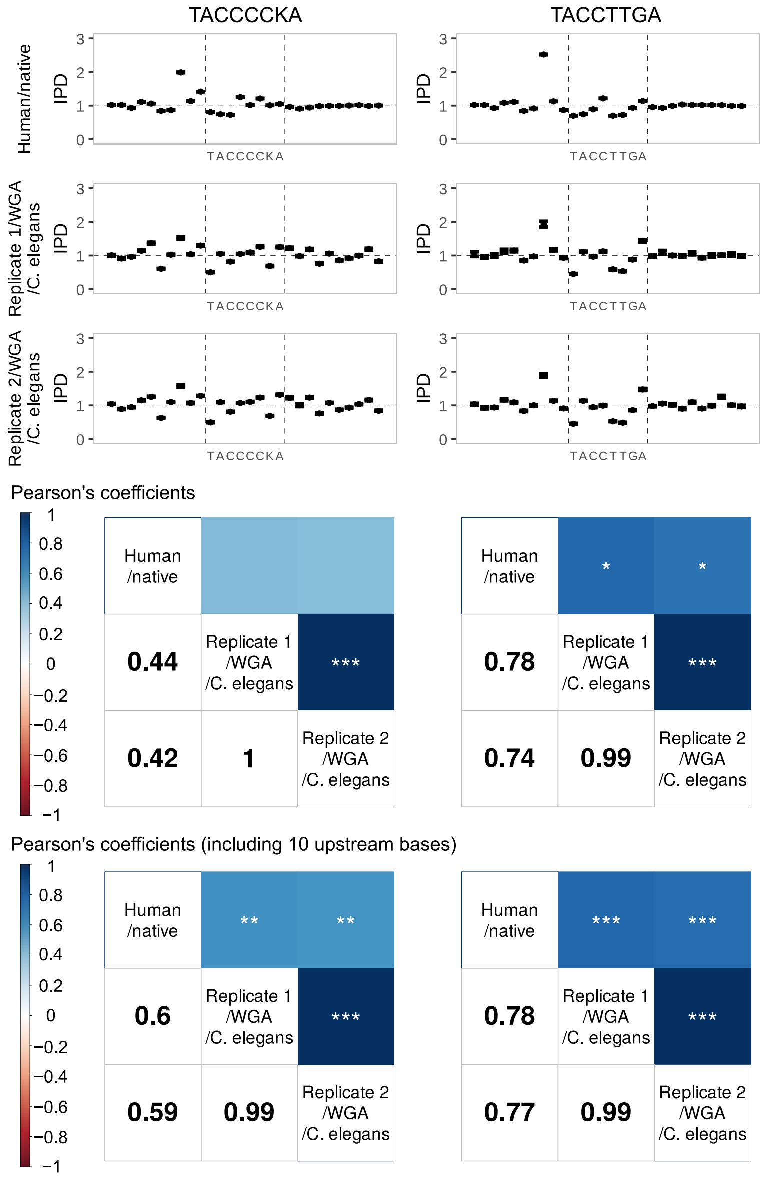

Supplementary Figure 7M: Similar to Supplementary Figure 7A, we show the IPD distributions and correlation coefficients in the three samples for each motif with extreme IPDs.

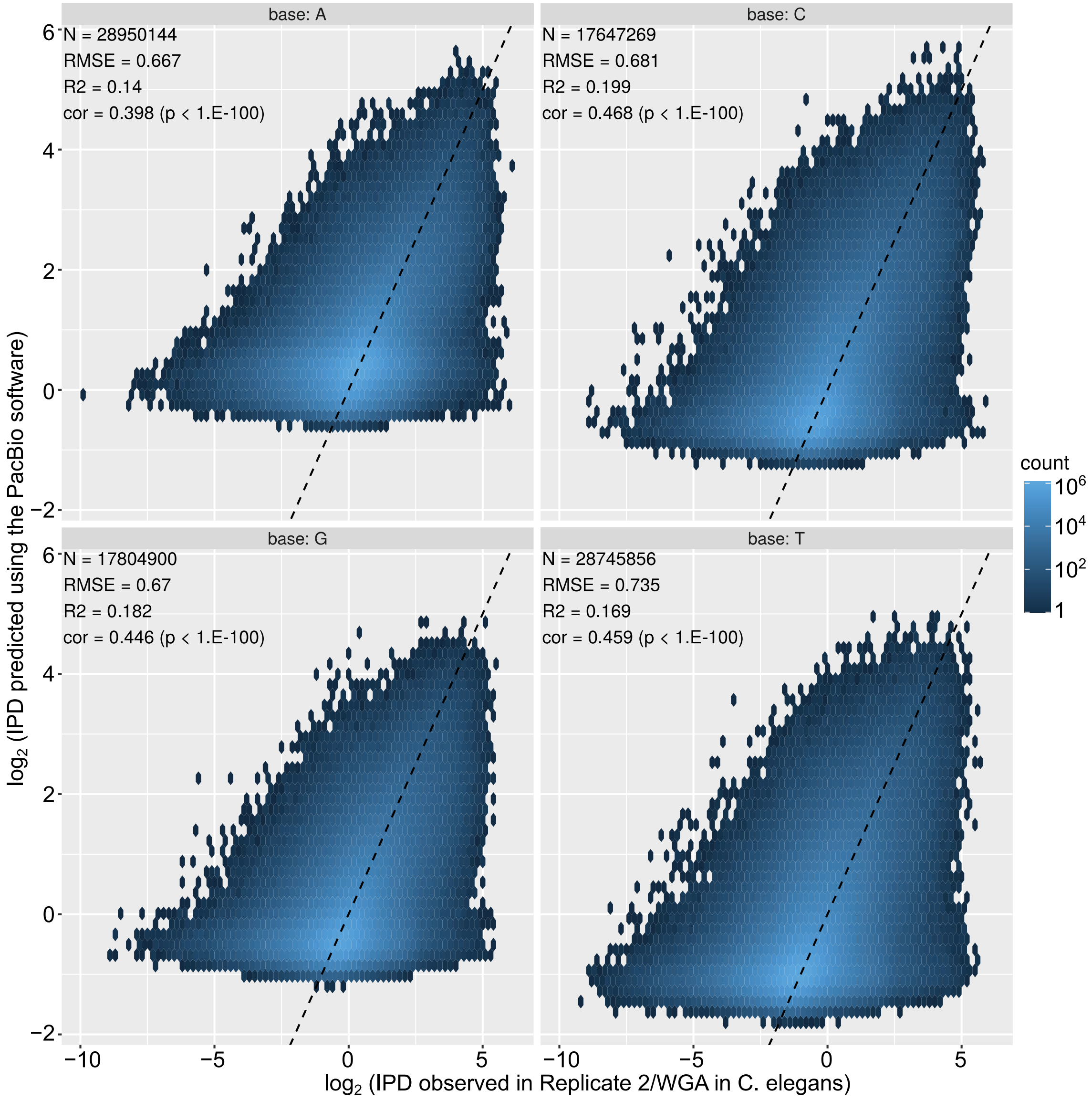

Supplementary Figure 8: Similar to Supplementary Figure 3A, but we here compare $\log_2$ (IPDs observed in replicate 2/WGA in *C. elegans*) with $\log_2$ (IPDs predicted using the PacBio software).

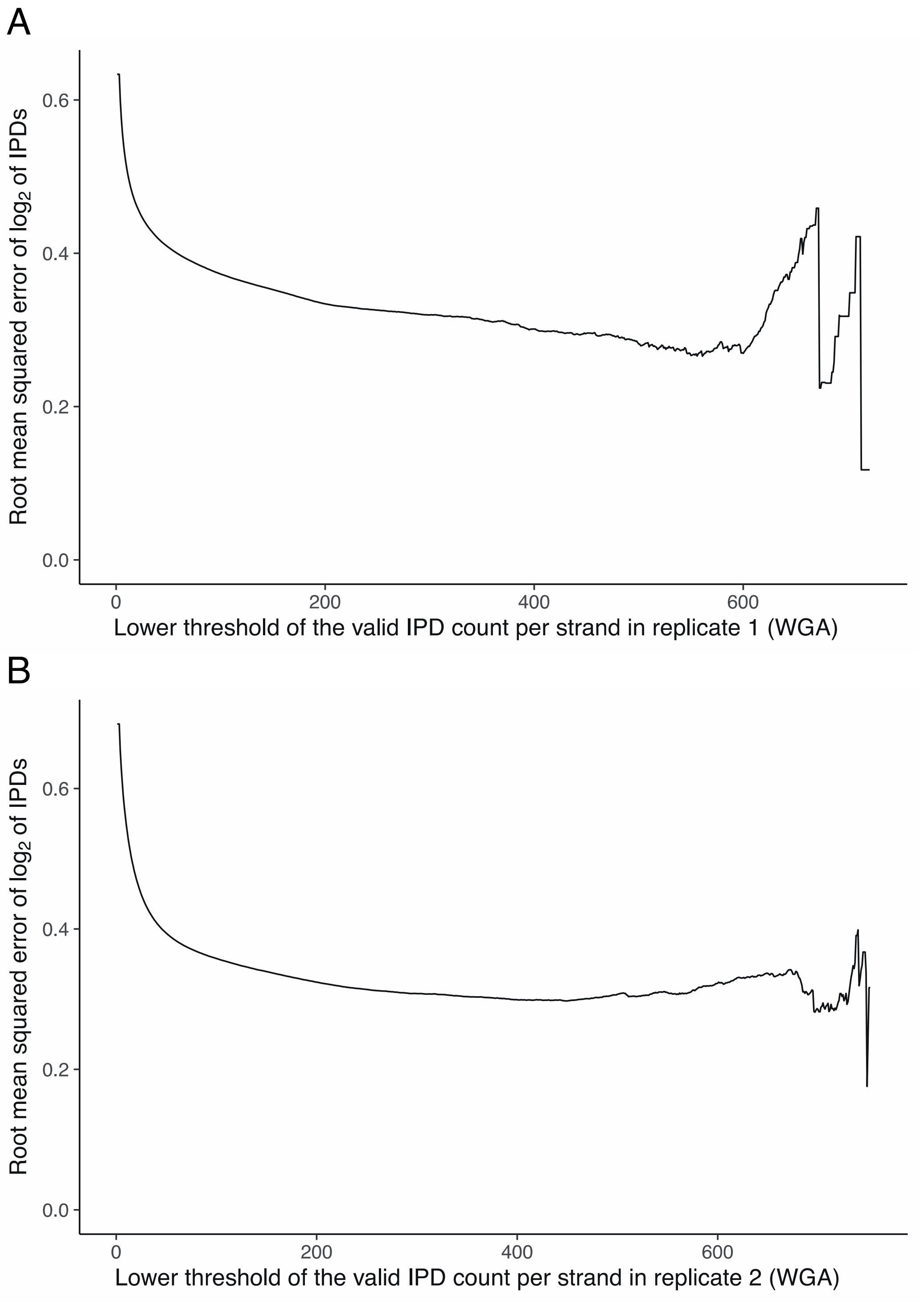

Supplementary Figure 9: Root-mean-square error between $\log_2$ (observed IPDs) and $\log_2$ (IPDs predicted using the PacBio software) for different lower thresholds of valid IPD values per strand in replicate 1/WGA (A) or replicate 2/WGA (B) in *C. elegans*.

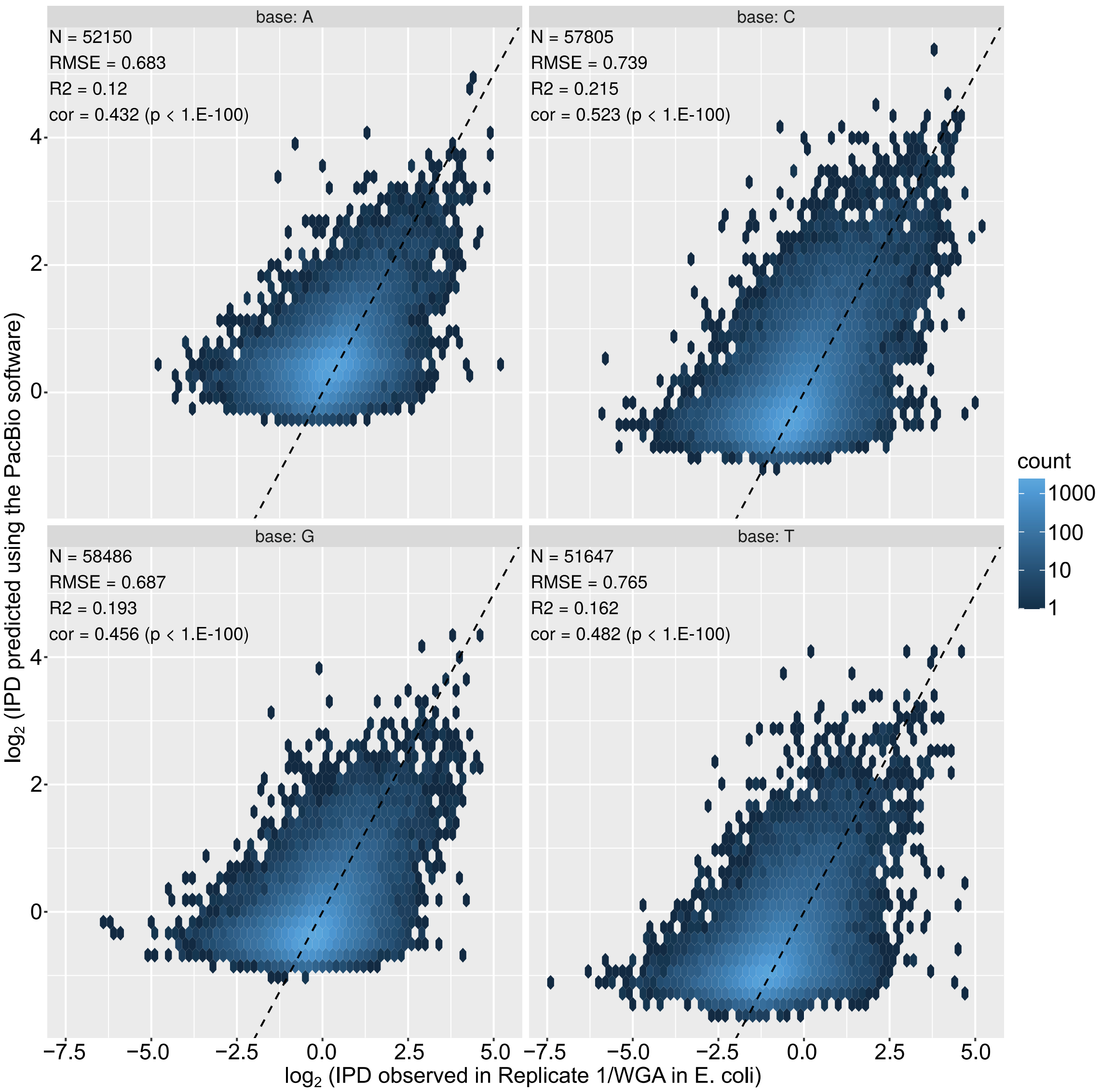

Supplementary Figure 10: Similar to Supplementary Figure 3A, but we here compare $\log_2$ (IPDs observed in replicate 1/WGA in *E. coli*) with $\log_2$ (IPDs predicted using the PacBio software).

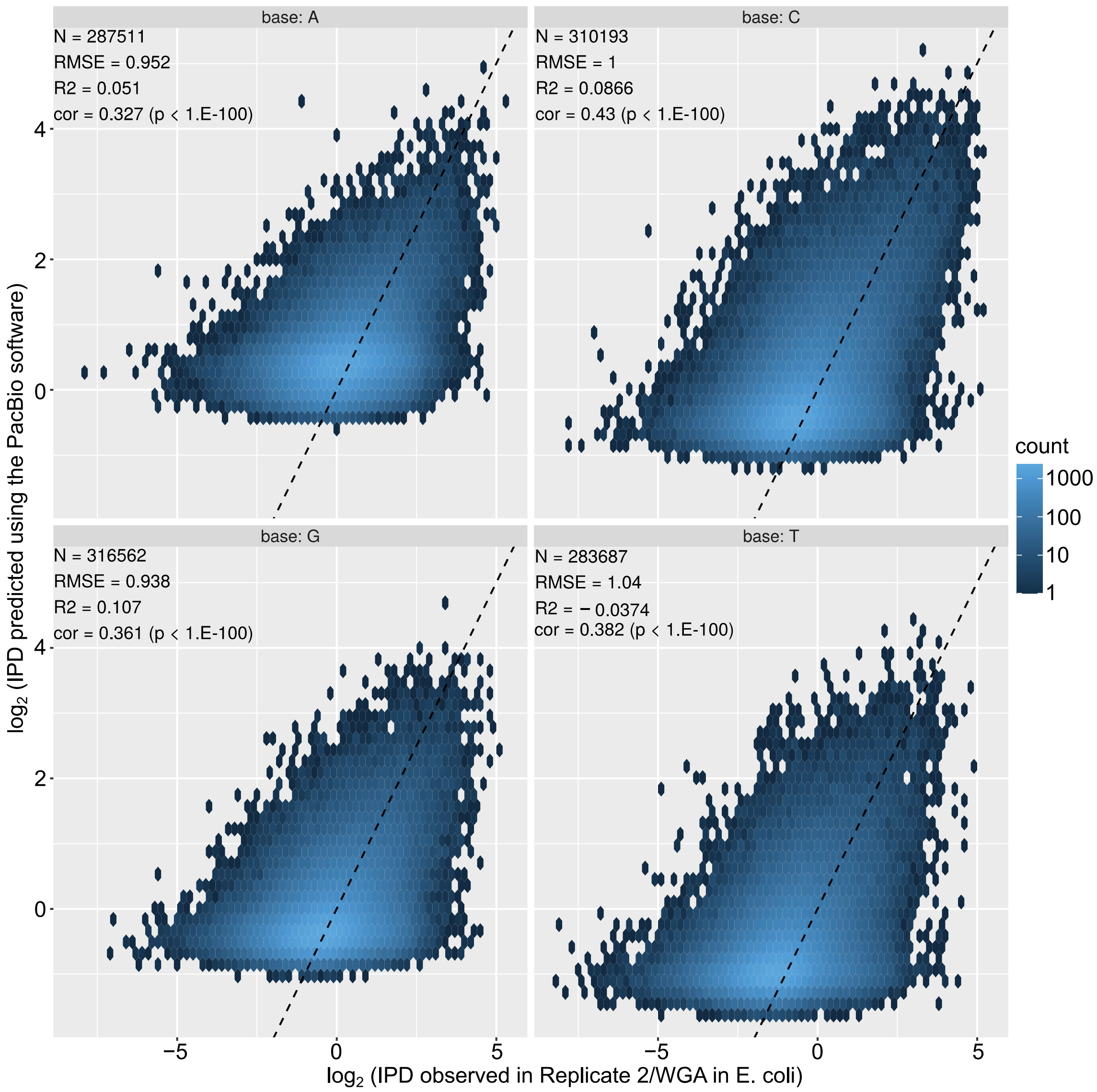

Supplementary Figure 11: Similar to Supplementary Figure 3A, but we here compare $\log_2$ (IPDs observed in replicate 2/WGA in *E. coli*) with $\log_2$ (IPDs predicted using the PacBio software).

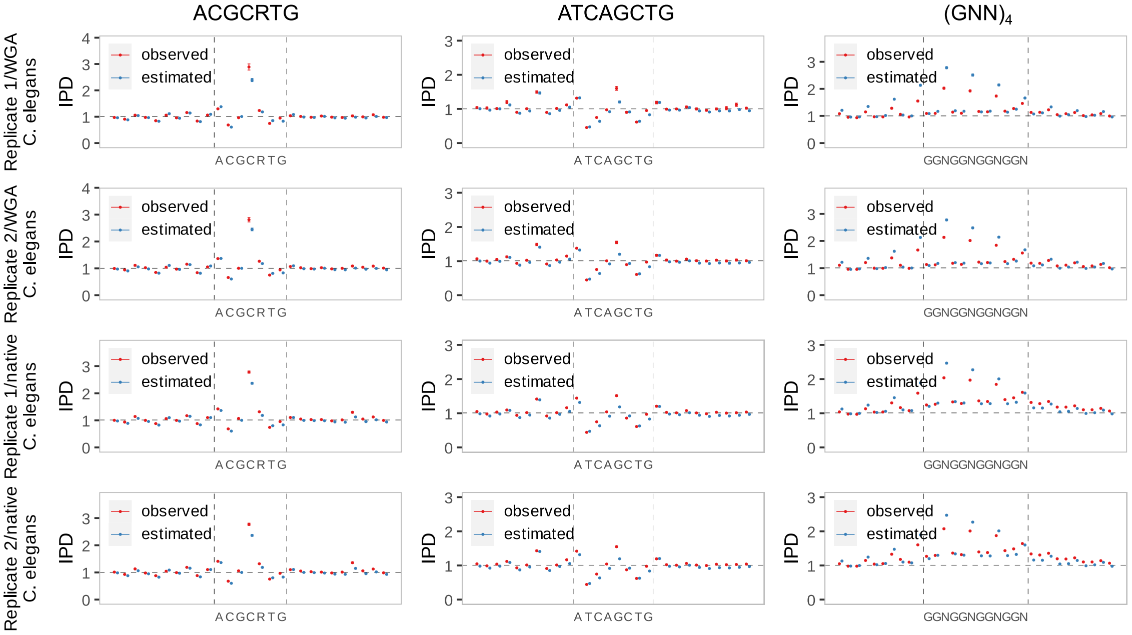

Supplementary Figure 12A: Discrepancy between observed (red) and predicted (blue) IPDs of three motifs in the *C. elegans* genome of the four samples that are replicate 1/WGA, replicate 2/WGA, replicate 1/native, and replicate 2/native from top to bottom.

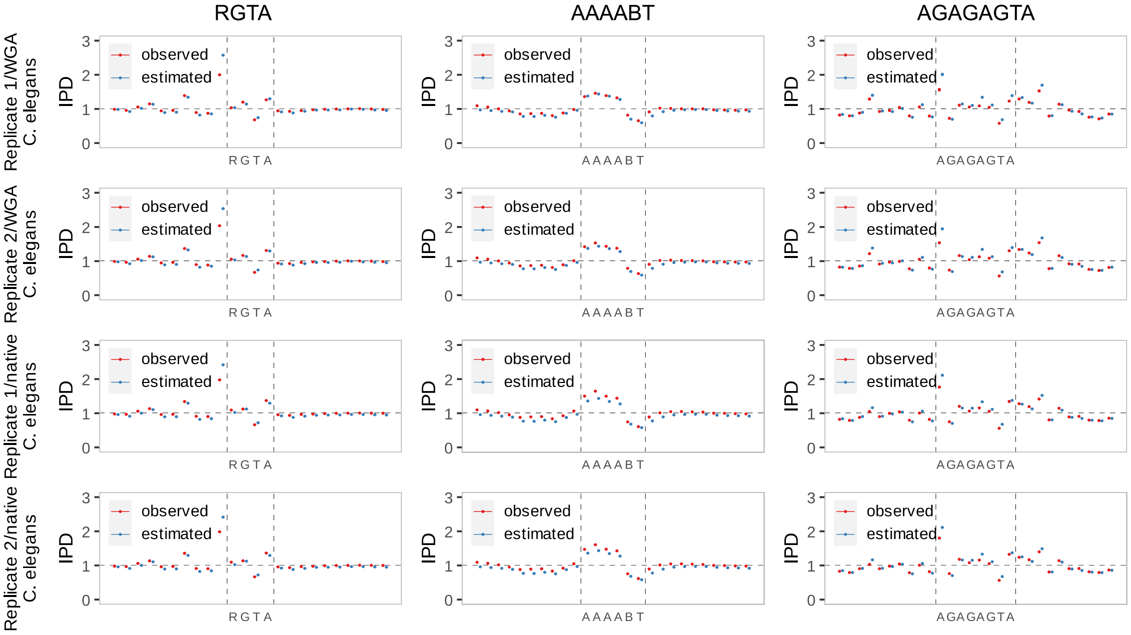

Supplementary Figure 12B: Similar to Supplementary Figure 12A, discrepancy between observed (red) and predicted (blue) IPDs of three motifs in the *C. elegans* genome of the four samples.

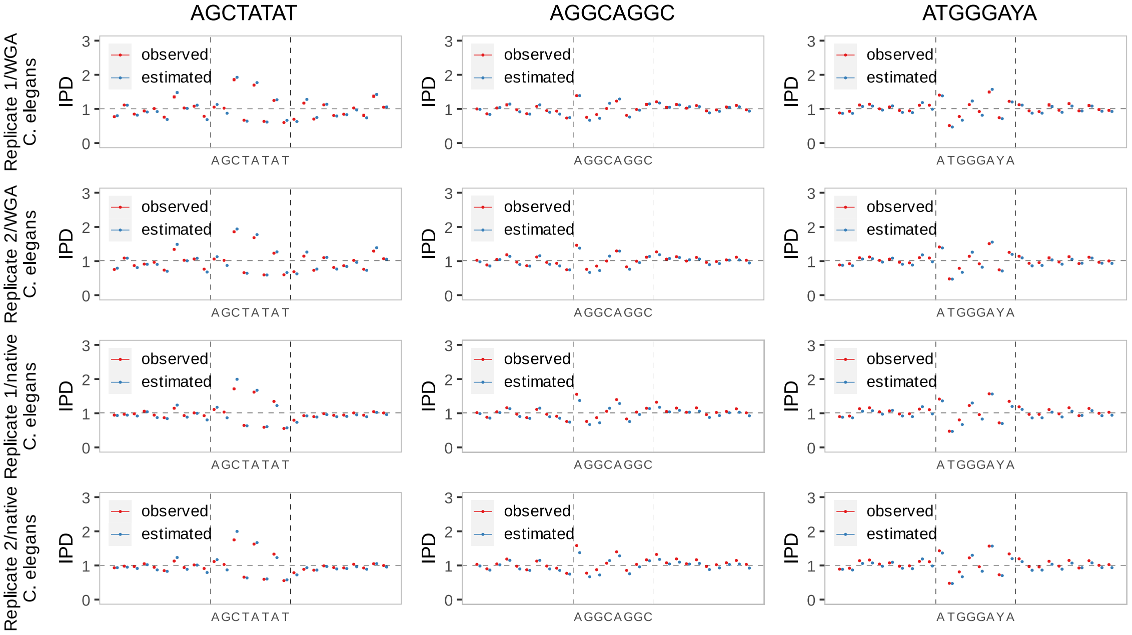

Supplementary Figure 12C: Similar to Supplementary Figure 12A, discrepancy between observed (red) and predicted (blue) IPDs of three motifs in the *C. elegans* genome of the four samples.

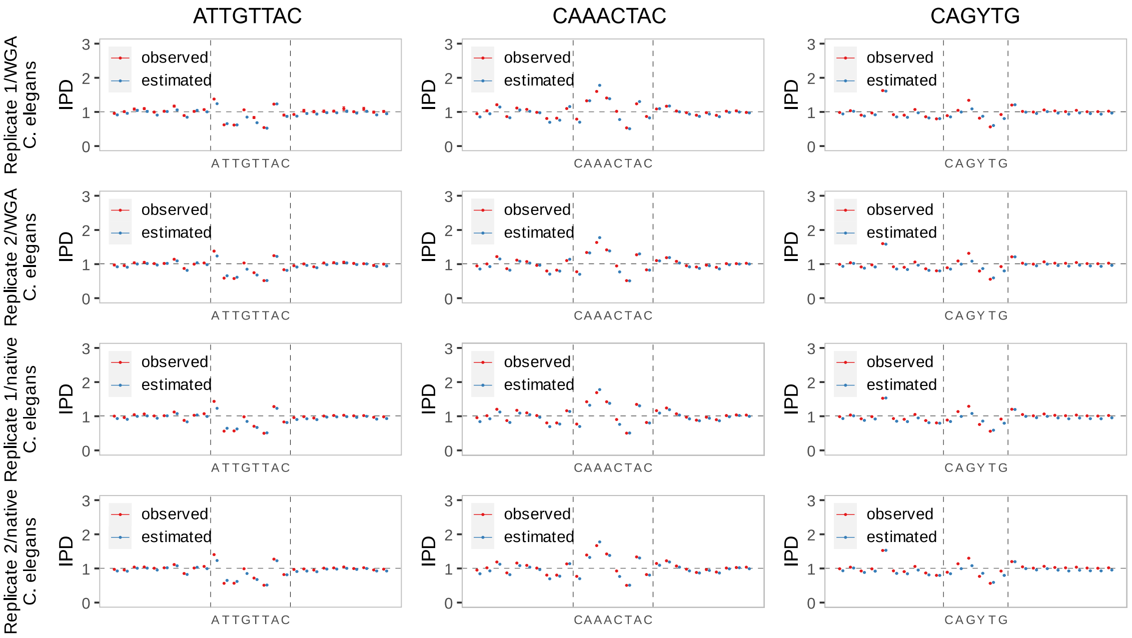

Supplementary Figure 12D: Similar to Supplementary Figure 12A, discrepancy between observed (red) and predicted (blue) IPDs of three motifs in the *C. elegans* genome of the four samples.

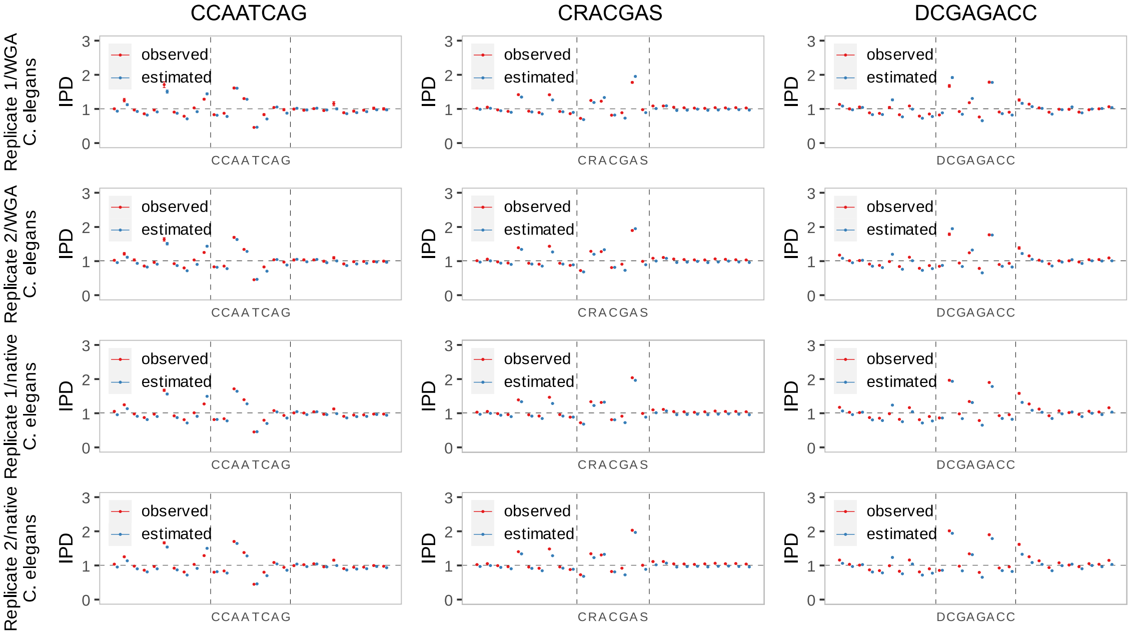

Supplementary Figure 12E: Similar to Supplementary Figure 12A, discrepancy between observed (red) and predicted (blue) IPDs of three motifs in the *C. elegans* genome of the four samples.

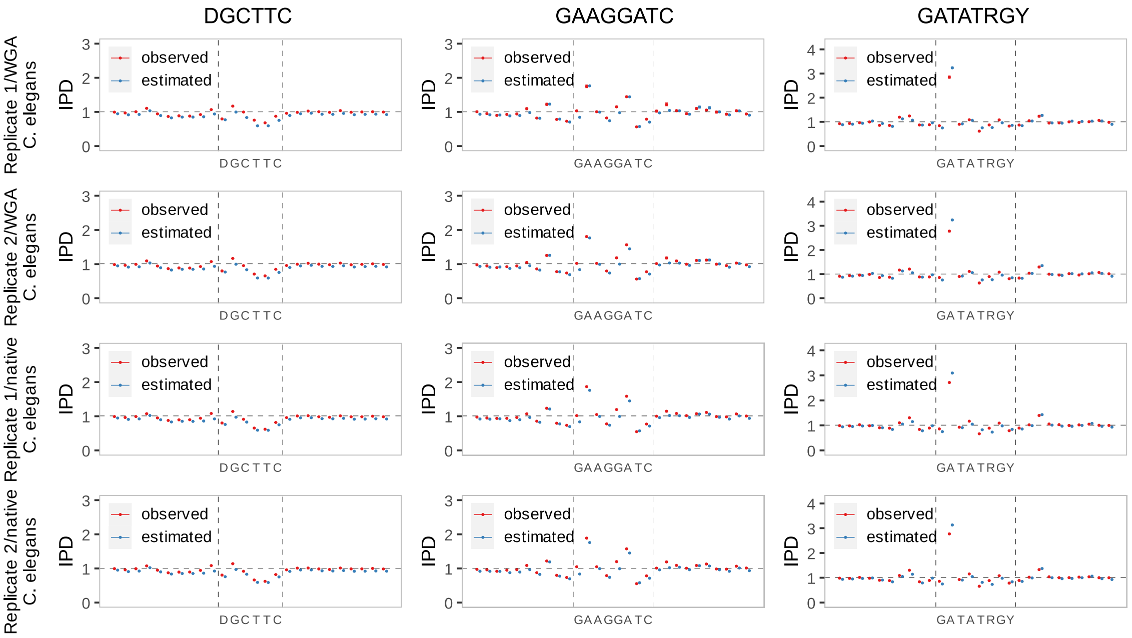

Supplementary Figure 12F: Similar to Supplementary Figure 12A, discrepancy between observed (red) and predicted (blue) IPDs of three motifs in the *C. elegans* genome of the four samples.

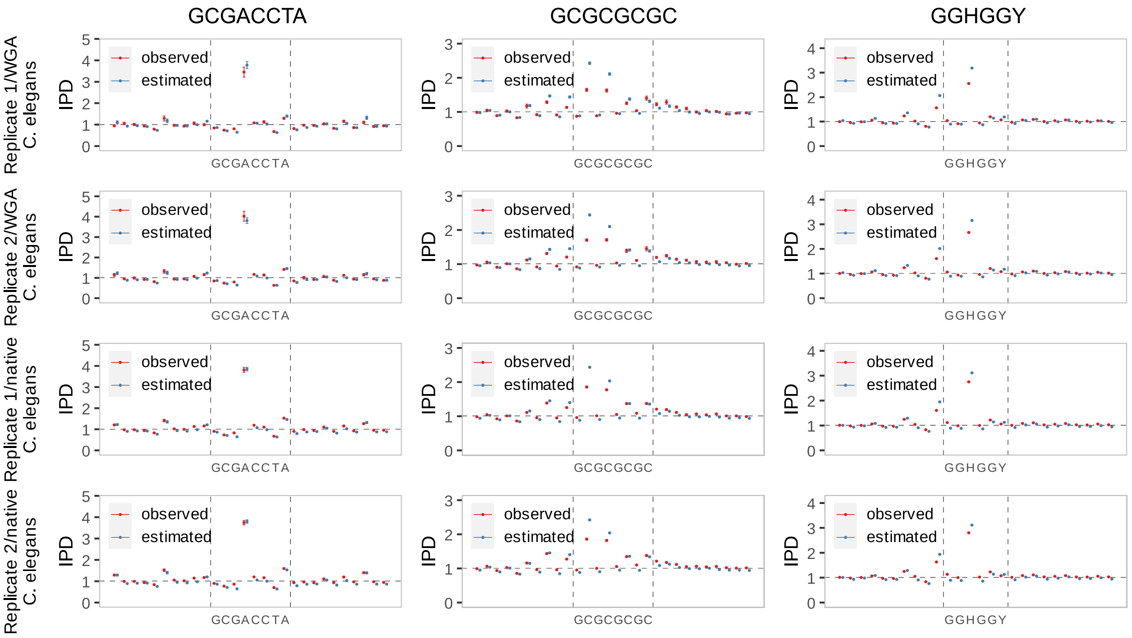

Supplementary Figure 12G: Similar to Supplementary Figure 12A, discrepancy between observed (red) and predicted (blue) IPDs of three motifs in the *C. elegans* genome of the four samples.

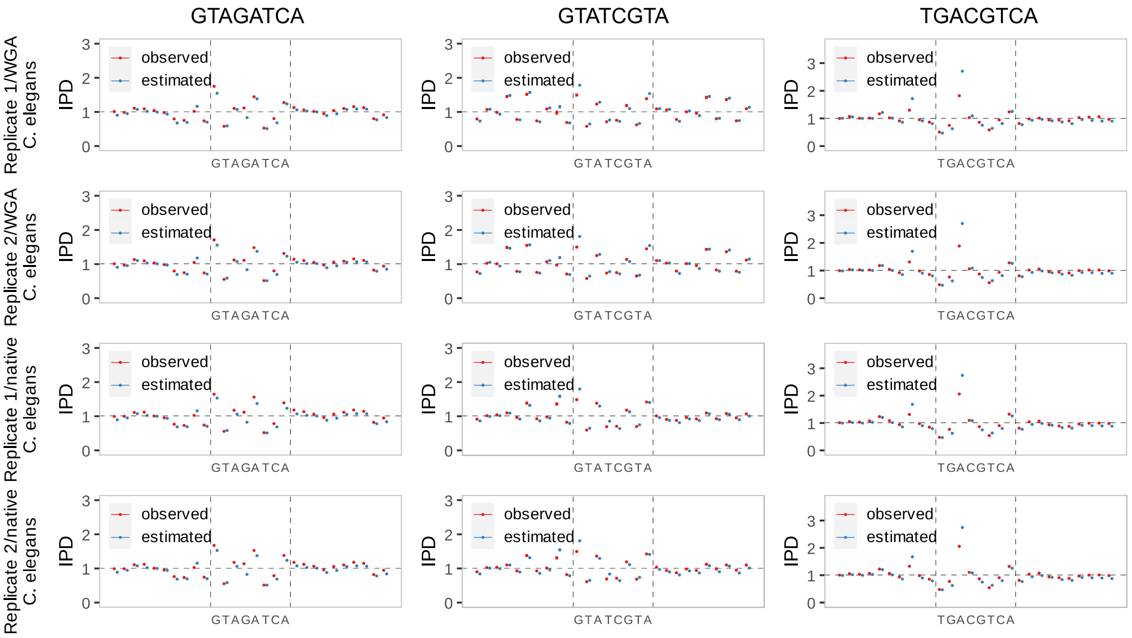

Supplementary Figure 12H: Similar to Supplementary Figure 12A, discrepancy between observed (red) and predicted (blue) IPDs of three motifs in the *C. elegans* genome of the four samples.

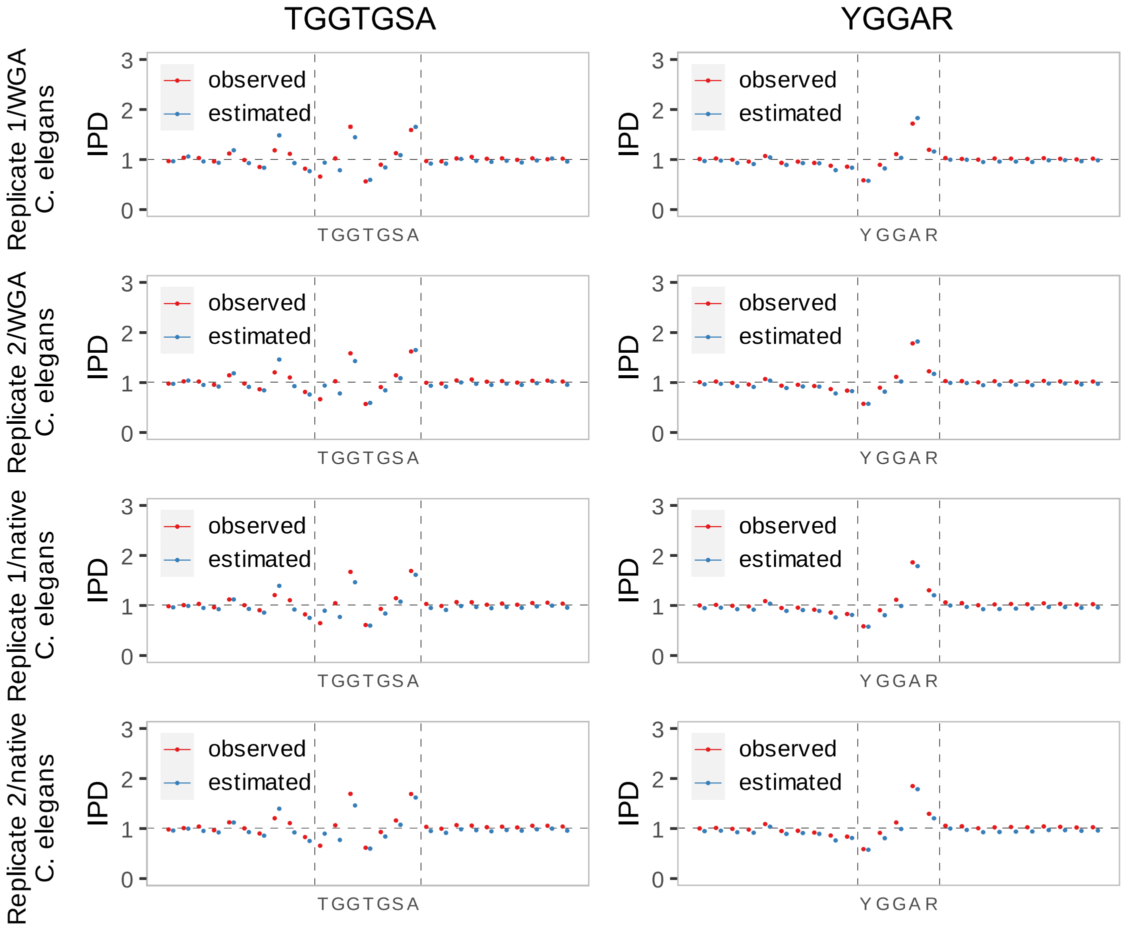

Supplementary Figure 12I: Similar to Supplementary Figure 12A, discrepancy between observed (red) and predicted (blue) IPDs of two motifs in the *C. elegans* genome of the four samples.

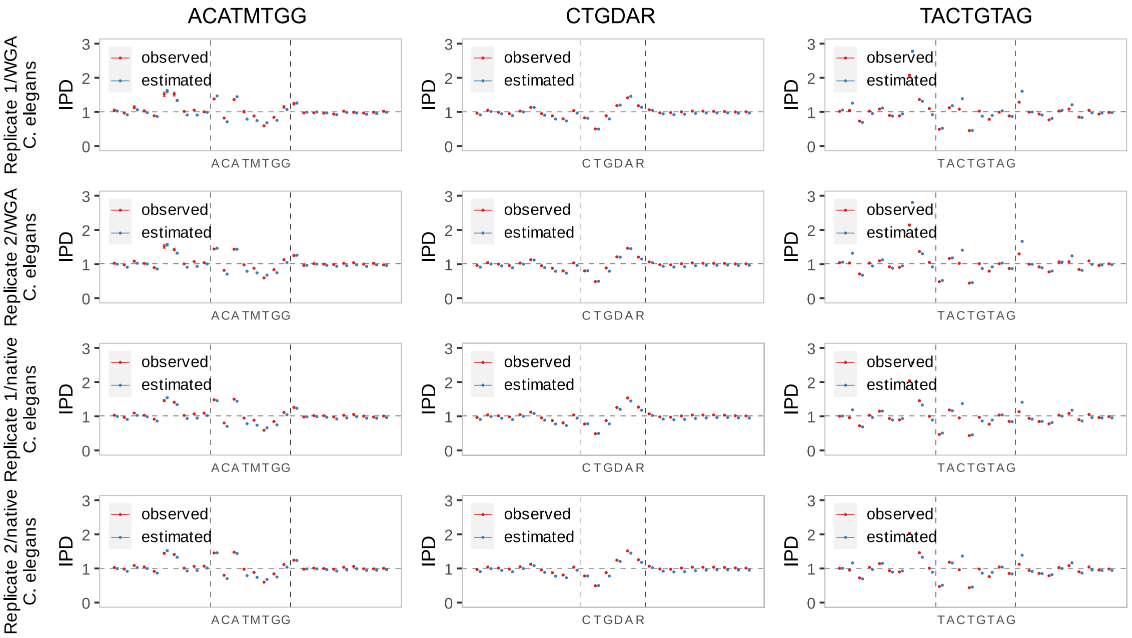

Supplementary Figure 12J: Similar to Supplementary Figure 12A, discrepancy between observed (red) and predicted (blue) IPDs of three motifs in the *C. elegans* genome of the four samples.

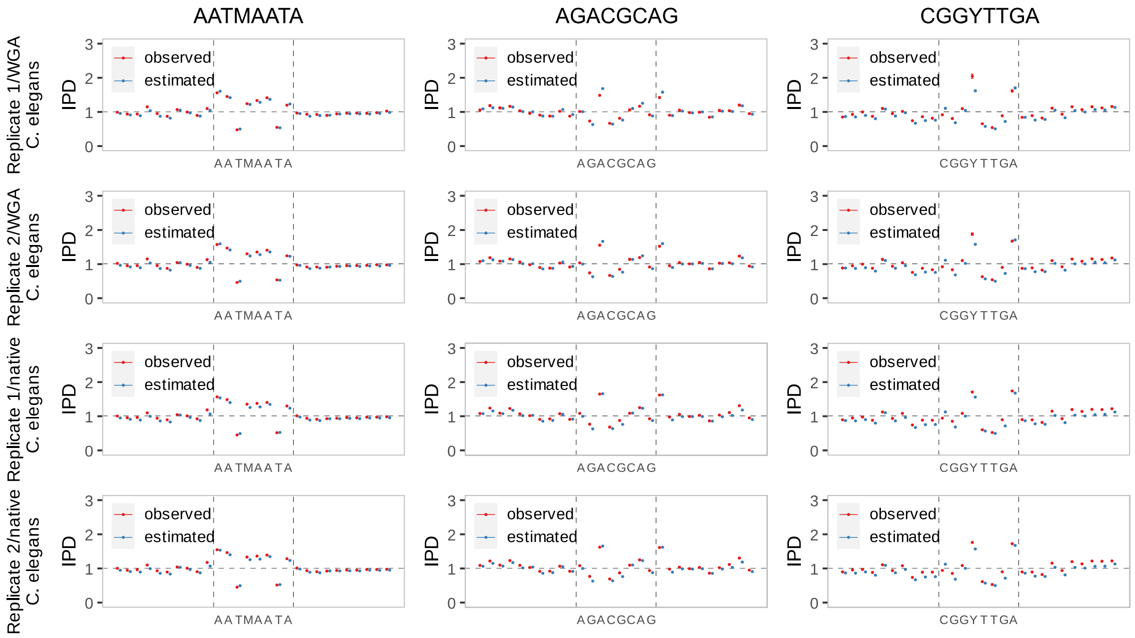

Supplementary Figure 12K: Similar to Supplementary Figure 12A, discrepancy between observed (red) and predicted (blue) IPDs of three motifs in the *C. elegans* genome of the four samples.

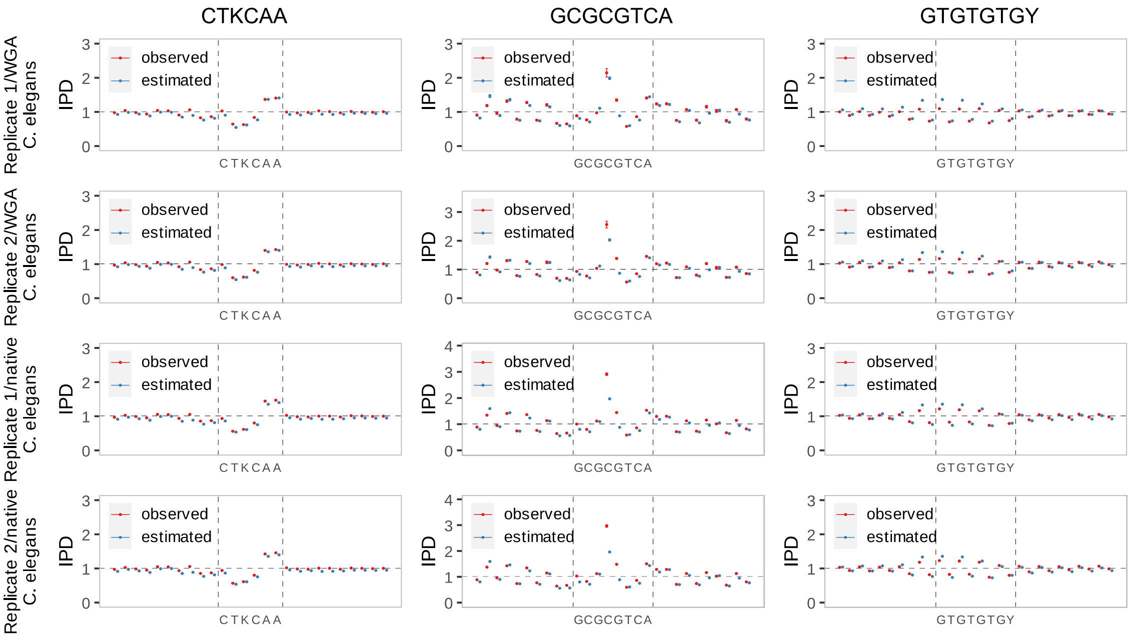

Supplementary Figure 12L: Similar to Supplementary Figure 12A, discrepancy between observed (red) and predicted (blue) IPDs of three motifs in the *C. elegans* genome of the four samples.

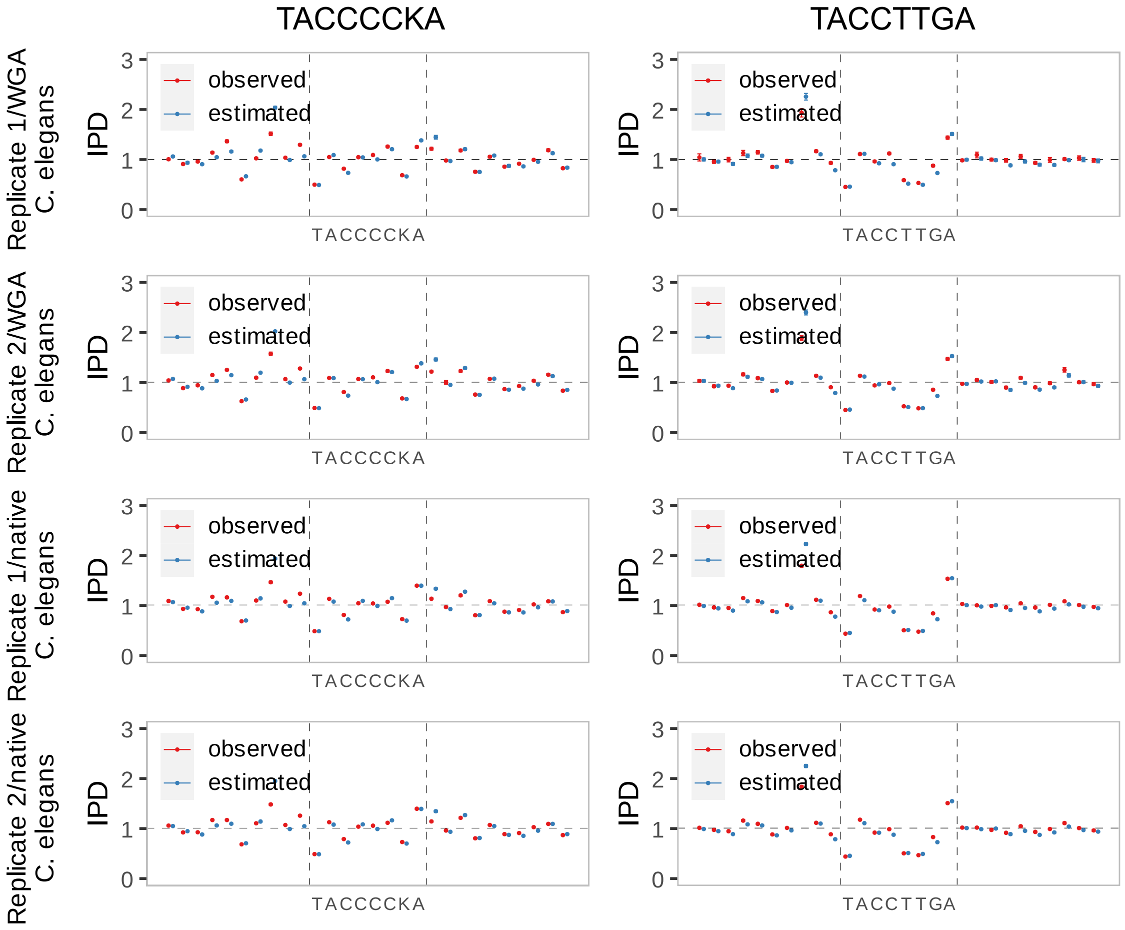

Supplementary Figure 12M: Similar to Supplementary Figure 12A, discrepancy between observed (red) and predicted (blue) IPDs of two motifs in the *C. elegans* genome of the four samples.

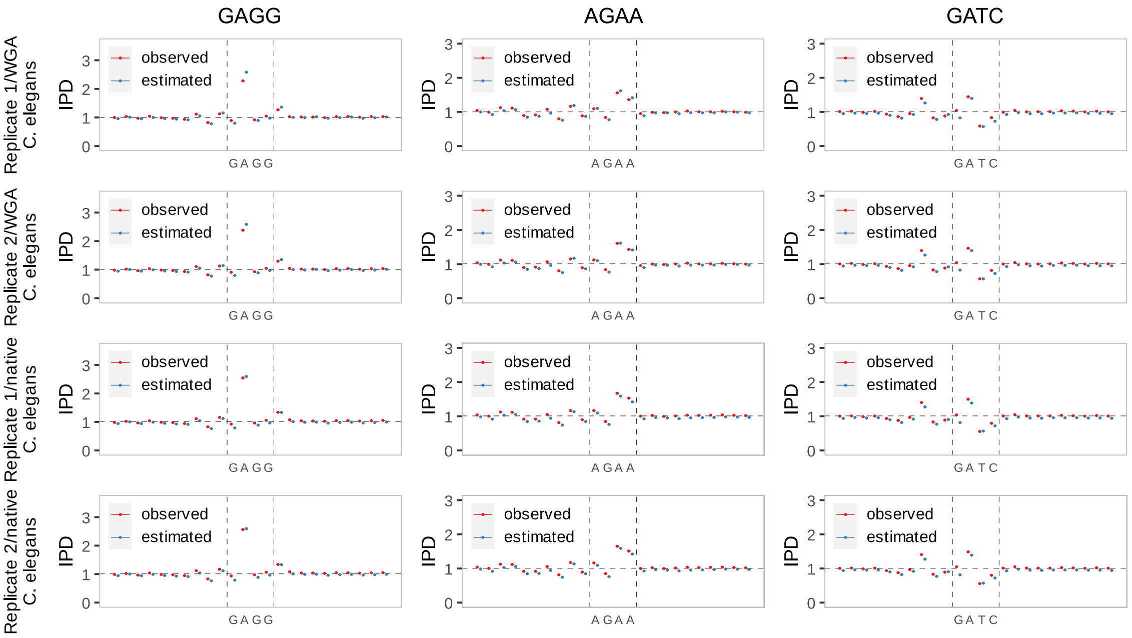

Supplementary Figure 13A: Discrepancy between observed (red) and predicted (blue) IPDs of GAGG, AGAA, and GATC in the *C. elegans* genome of the four samples that are replicate 1/WGA, replicate 2/WGA, replicate 1/native, and replicate 2/native from top to bottom.

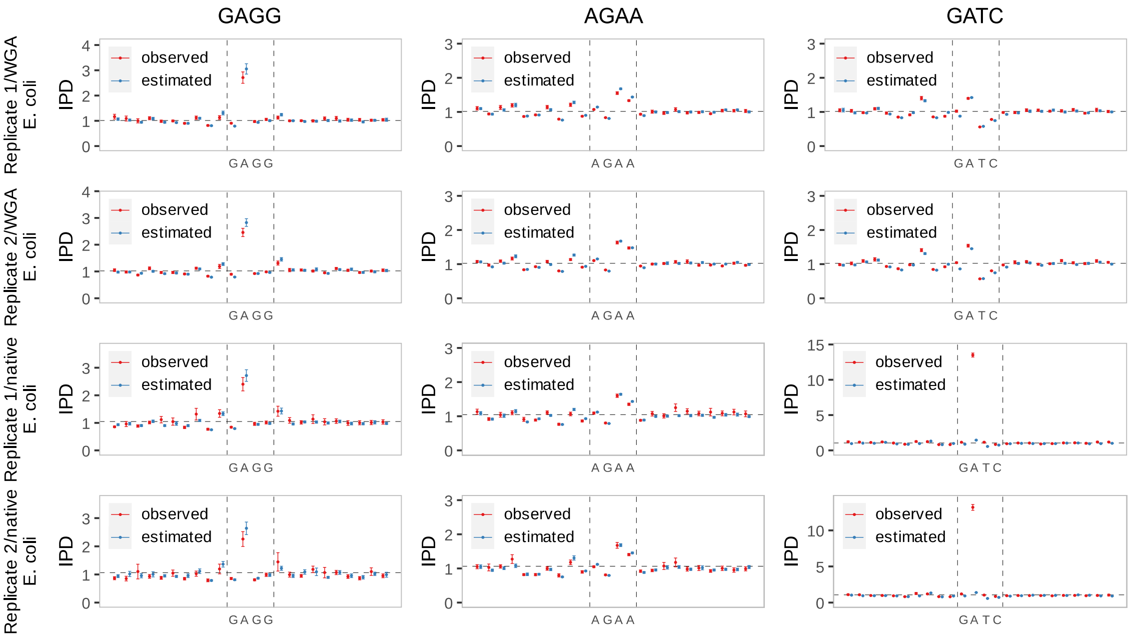

Supplementary Figure 13B: Discrepancy between observed (red) and predicted (blue) IPDs of GAGG, AGAA, and GATC in the *E. coli* genome of the four samples that are replicate 1/WGA, replicate 2/WGA, replicate 1/native, and replicate 2/native from top to bottom.

A

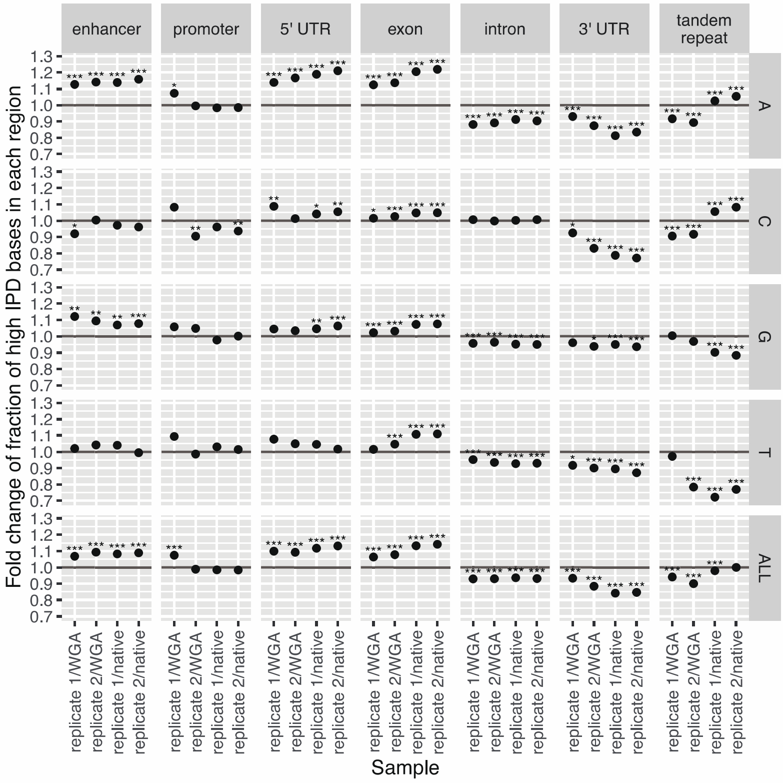

B

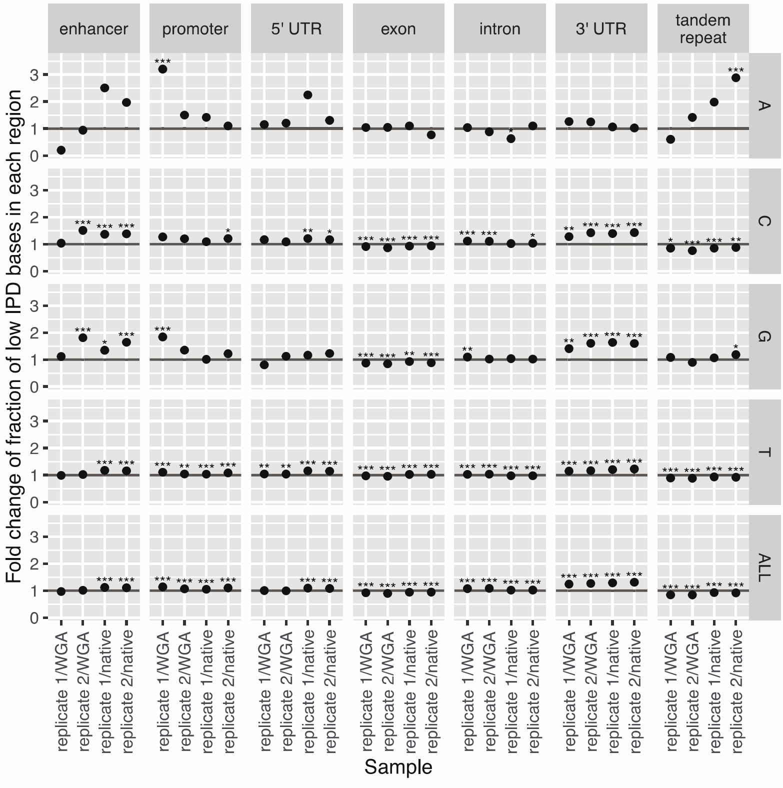

Supplementary Figure 14: Fold changes of the fraction of bases with high (**A**) and low IPDs (**B**) in each genomic region, compared to the fraction in the entire genome. To eliminate the potential effects of DNA methylation on IPD values, our analyses included two WGA samples, replicate 1/WGA and replicate 2/WGA, as well as two native samples (replicate 1/native and replicate 2/native) in the *C. elegans* genome, which are shown from left to right in each column. IPD values are grouped by base in the y-axis, while the x-axis represents the different genomic regions. Dots represent fold changes; stars on dots respectively represent FDR q-values. ***, **, and * indicate $q \leq 0.001$, $q \leq 0.01$, and $q \leq 0.05$ (Benjamini-Hochberg procedure and a binomial test).

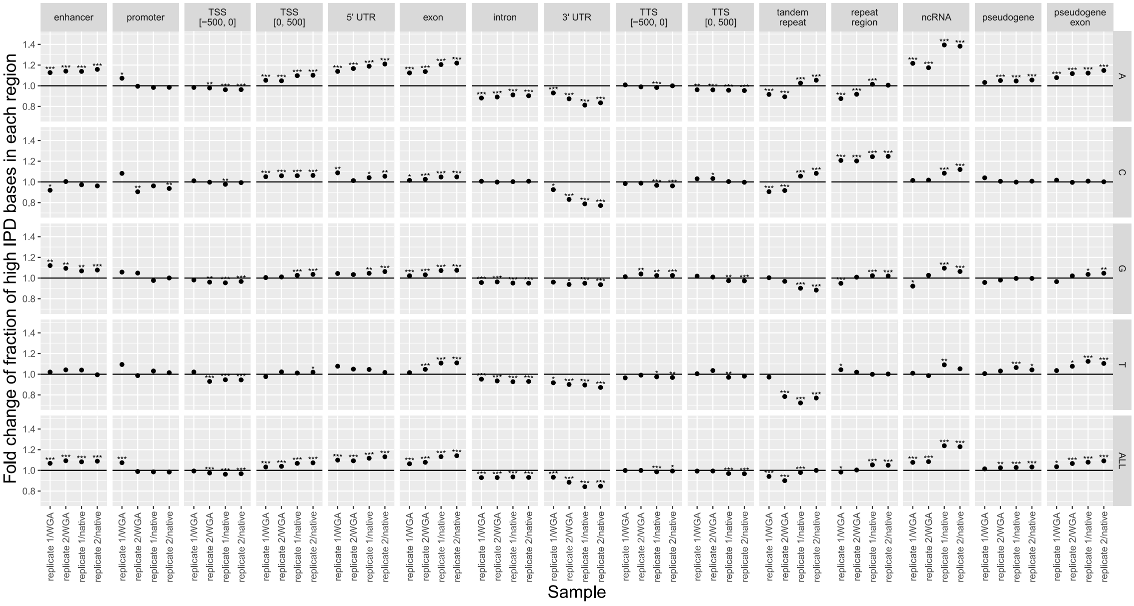

Supplementary Figure 15: Fold change of the fraction of bases with "high" IPD values in each region, compared to the fraction in the genome. We here compare replicate 1/WGA, replicate 2/WGA, replicate 1/native, and replicate 2/native in the *C. elegans* genome, which are displayed from left to right in each column of genomic region. Vertical facets represent types of bases, while horizontal facets represent the genomic region. In each facet, the horizontal axis represents the sample; each dot represents the fold change. Stars on dots represent FDR q-values determined using the Benjamini-Hochberg procedure. (***): $q \leq 0.001$, (**): $q \leq 0.01$, (*): $q \leq 0.05$.

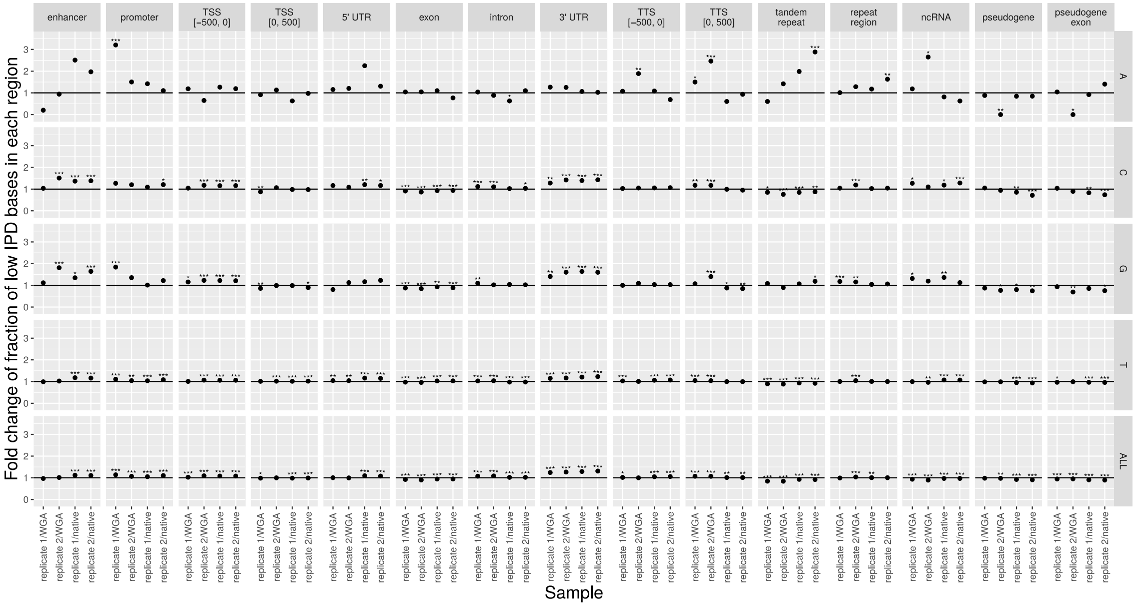

Supplementary Figure 16: This figure is similar to Supplementary Figure 15, but differs in that we focus on bases with "low" IPDs in each region.

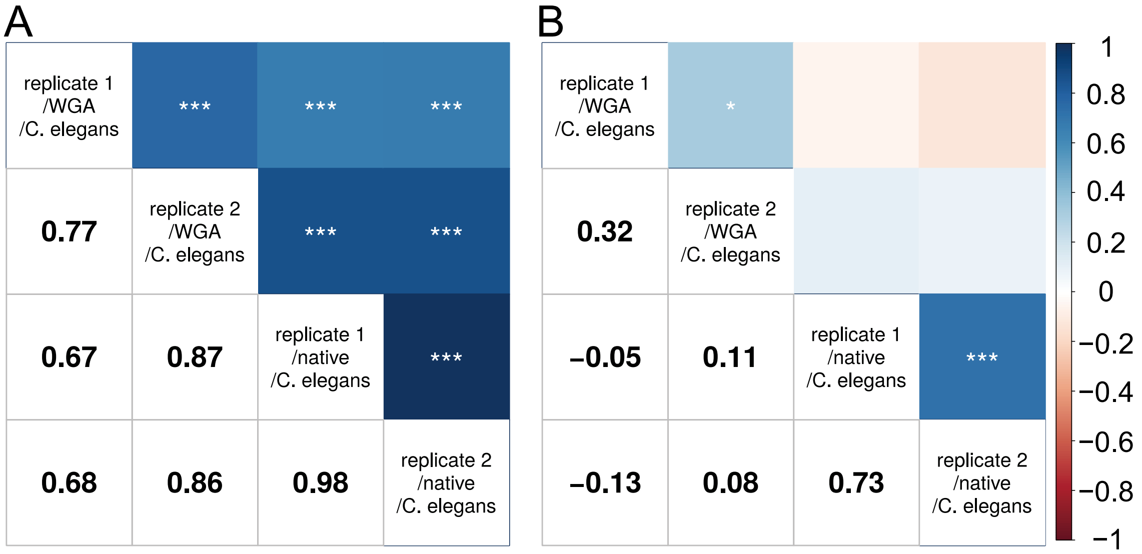

Supplementary Figure 17: Pearson's coefficients showing the relationship between fold changes in the fraction of bases with high (**A**) or low (**B**) IPD in different classes of genomic regions and the fraction in the whole genome, between each pair of samples among the four samples in this study. Fold changes using all bases (denoted ALL) were excluded. Correlation coefficients are shown with colors in upper triangle and with numbers in lower triangle. Stars in upper triangle represent the *p*-value for a coefficient of zero. (***): $p \leq 0.001$, (**): $p \leq 0.01$, (*): $p \leq 0.05$.
